# Identification of Some Glutamic Acid Derivatives with Biological Potential by Computational Methods

**DOI:** 10.3390/molecules28104123

**Published:** 2023-05-16

**Authors:** Octavia-Laura Moldovan, Alexandra Sandulea, Ioana-Andreea Lungu, Șerban Andrei Gâz, Aura Rusu

**Affiliations:** 1 Medicine and Pharmacy Doctoral School, George Emil Palade University of Medicine, Pharmacy, Science and Technology of Targu Mures, 540142 Targu Mures, Romania; ioana-andreea.lungu@umfst.ro; 2 Pharmaceutical and Therapeutic Chemistry Department, Faculty of Pharmacy, George Emil Palade University of Medicine, Pharmacy, Science and Technology of Targu Mures, 540142 Targu Mures, Romania; sandulea.alex@yahoo.com (A.S.); aura.rusu@umfst.ro (A.R.); 3 Organic Chemistry Department, Faculty of Pharmacy, George Emil Palade University of Medicine, Pharmacy, Science and Technology of Targu Mures, 540142 Targu Mures, Romania; andrei.gaz-florea@umfst.ro

**Keywords:** glutamic acid, glutamine, anti-tumour potential, anti-cancer effect, molecular docking, computational methods

## Abstract

Glutamic acid is a non-essential amino acid involved in multiple metabolic pathways. Of high importance is its relationship with glutamine, an essential fuel for cancer cell development. Compounds that can modify glutamine or glutamic acid behaviour in cancer cells have resulted in attractive anticancer therapeutic alternatives. Based on this idea, we theoretically formulated 123 glutamic acid derivatives using Biovia Draw. Suitable candidates for our research were selected among them. For this, online platforms and programs were used to describe specific properties and their behaviour in the human organism. Nine compounds proved to have suitable or easy to optimise properties. The selected compounds showed cytotoxicity against breast adenocarcinoma, lung cancer cell lines, colon carcinoma, and T cells from acute leukaemia. Compound 2Ba5 exhibited the lowest toxicity, and derivative 4Db6 exhibited the most intense bioactivity. Molecular docking studies were also performed. The binding site of the 4Db6 compound in the glutamine synthetase structure was determined, with the D subunit and cluster 1 being the most promising. In conclusion, glutamic acid is an amino acid that can be manipulated very easily. Therefore, molecules derived from its structure have great potential to become innovative drugs, and further research on these will be conducted.

## 1. Introduction

Throughout history, cancer has been a major health problem. It has been shown that there is a positive correlation between cancer incidence and age [1,2,3]. The individual risk of cancer is also influenced by family history, genetic susceptibility or behaviour, and exposure to carcinogenic factors [4]. Furthermore, the Krebs cycle and amino acids are proven to significantly affect cancer metabolism. Thus, interfering with amino acid metabolic pathways is an active area of study in cancer metabolism [5]. 

Amino acids are essential for cancer development because they can function as opportunistic fuel sources for cells [5]. Cancer cells use multiple strategies to obtain amino acids [6]. Higher-grade cancer cells must be able to supply additional metabolites for bioenergy and synthesise the necessary biosynthetic precursors of proteins, nucleic acids, and membrane lipids to grow substantially [7]. In cancer cells, glutamine is the major amino acid that serves as an anaplerosis metabolite and drives the tricarboxylic acid (TCA) cycle to sustain mitochondrial ATP for energy production [5]. Glutamine is the most abundant amino acid in plasma. The majority of circulating glutamine is produced in muscles and, additionally, in the lungs [8]. However, it has been observed that a reduced exogenous supply of glutamine can impair malignant cells’ survival or tumorigenic potential [6]. 

Glutamine is a versatile biosynthetic substrate for carbon and nitrogen atoms to generate important precursors for macromolecule biosynthesis [9]. It is the nitrogen donor for the biosynthesis of purines, pyrimidines, nicotinamide adenine dinucleotide, asparagine, and hexosamines via its terminal amide group. A higher expression of enzymes that mediate nucleotide synthesis from glutamine positively correlates with increased proliferation in tumours [10]. Glutamine also drives the uptake of essential amino acids, helps recycle excessive ammonia and glutamate, and activates the mammalian target of rapamycin (mTOR) that is involved in gene transcription and intracellular signalling [8,9,10]. In this regard, compounds that interfere with glutamine metabolism have shown therapeutic potential in preclinical studies by disrupting these growth-promoting processes [9]. In addition to providing building blocks for cell growth, glutamine metabolism plays a critical role in maintaining cellular redox homeostasis, as glutamate is a precursor for glutathione (GSH) [8,11]. GSH is used to maintain redox homeostasis within the cell and to protect it from oxidative damage [12]. Because excessive free radicals lead to DNA damage, lipid peroxidation, and protein denaturation, tumour cells mitigate the excess of free radicals and maintain redox homeostasis principally by GSH synthesis [10]. In this regard, a process called glutaminolysis, catalysed by mitochondrial glutaminase, plays an essential role in the glutamine conversion to glutamate. Furthermore, it regulates reactive oxygen species homeostasis by providing the precursors glutamate and cysteine for GSH synthesis [13,14]. 

Cancer cells rely on glutaminase activity to maintain a high ratio of glutamate to α-ketoglutarate, which is essential for producing non-essential amino acids. This aspect explains glutamine’s anaplerotic function [10]. Glutamate generates α-ketoglutarate and fuels the TCA cycle through a transamination reaction. In the same way, transaminases, such as aspartate aminotransferase, facilitate the interconversion of aspartic acid. All these biochemical reactions maintain normal metabolism, allowing glutamate to be converted to other amino acids if necessary. Thus, this enzyme is considered to play an essential role in some types of cancer metabolism, such as in pancreatic cancer [5]. 

**Figure 1:**
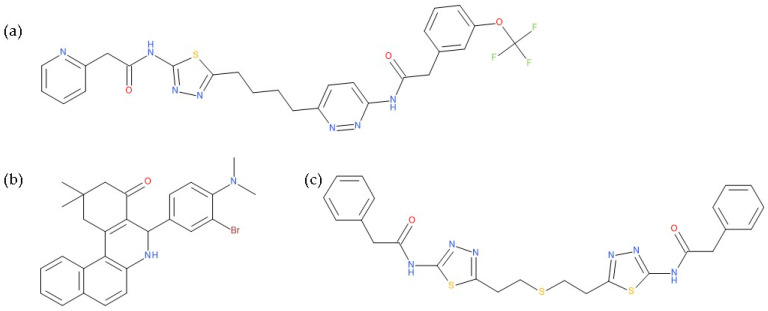
The chemical structures of the glutaminase inhibitor compounds (**a**) CB-839, (**b**) 968, and (**c**) BPTES.

Glutamine synthetase (GS) is another critical enzyme involved in glutamine metabolism is because it converts glutamate to glutamine. This biochemical reaction is essential as glutamine is the body’s non-toxic form of ammonia transport. It has also been found that GS activity is important for the proangiogenic, immunosuppressive, and pro-metastatic function of M2-like macrophages [8]. The term “glutamine addiction” has been used to describe the enhanced usage of glutamine in cancer in an anaplerotic sense [15]. However, the inherent properties of tumour cells differ, as the specific mechanism that a tumour cell chooses is dictated by tumour type, oncogene/tumour suppressor status, tumour site, and stage of tumour development [9]. Some cancer types mainly depend on glutamine metabolism for tumour cell survival and proliferation. For example, pancreas cancer, lung cancer, colon cancer, glioblastoma, acute myeloid leukaemia, ovarian cancer or triple-negative breast cancer, which do not express oestrogen, progesterone receptors or human epidermal growth factor receptor 2, mainly depend on glutamine, in contrast with other types of cancer [8,13,16]. Human liver cancer has also been found to be dependent on extracellular glutamine [13]. Therefore, glutamine uptake and glutaminase activity have been actively investigated as oncological targets [5]. Among the therapeutic strategies, one is targeting glutamine metabolism in tumours [13,17,18]. To date, the best-developed molecule is CB-839 (telaglenastat), which interferes with glutamine metabolism. This molecule is a potent, non-competitive allosteric inhibitor of the mitochondrial enzyme glutaminase and the only one that is currently being used in Phase I clinical trials in cancer patients [9,10,13]. CB-839 shows antiproliferative properties in triple-negative breast cancer by reducing glutamine consumption, glutamate production, and levels of TCA intermediates [13,15]. In addition, it exhibits significant efficacy in lung adenocarcinoma, chondrosarcoma and lymphoma cancer, but many liver cancer cell lines fail to respond to CB-839 treatment [13]. Besides CB-839, the compounds 968 (5-[3-bromo-4-(dimethylamino)phenyl]-2,2-dimethyl-1,3,5,6-tetrahydrobenzo[a]phenanthridin-4-one) and BPTES (Bis-2-(5-phenylacetamido-1,2,4-thiadiazol-2-yl)ethyl sulphide) are other glutaminase inhibitors used in preclinical studies [10,15,19] (Figure 1). BPTES led to GSH depletion, making some lung cancer cells more sensitive to radiation treatment. At the same time, compound 968 blocked oncogenic transformation in fibroblasts and reduced the growth of cancer cells [11,15]. 

**Figure 2:**
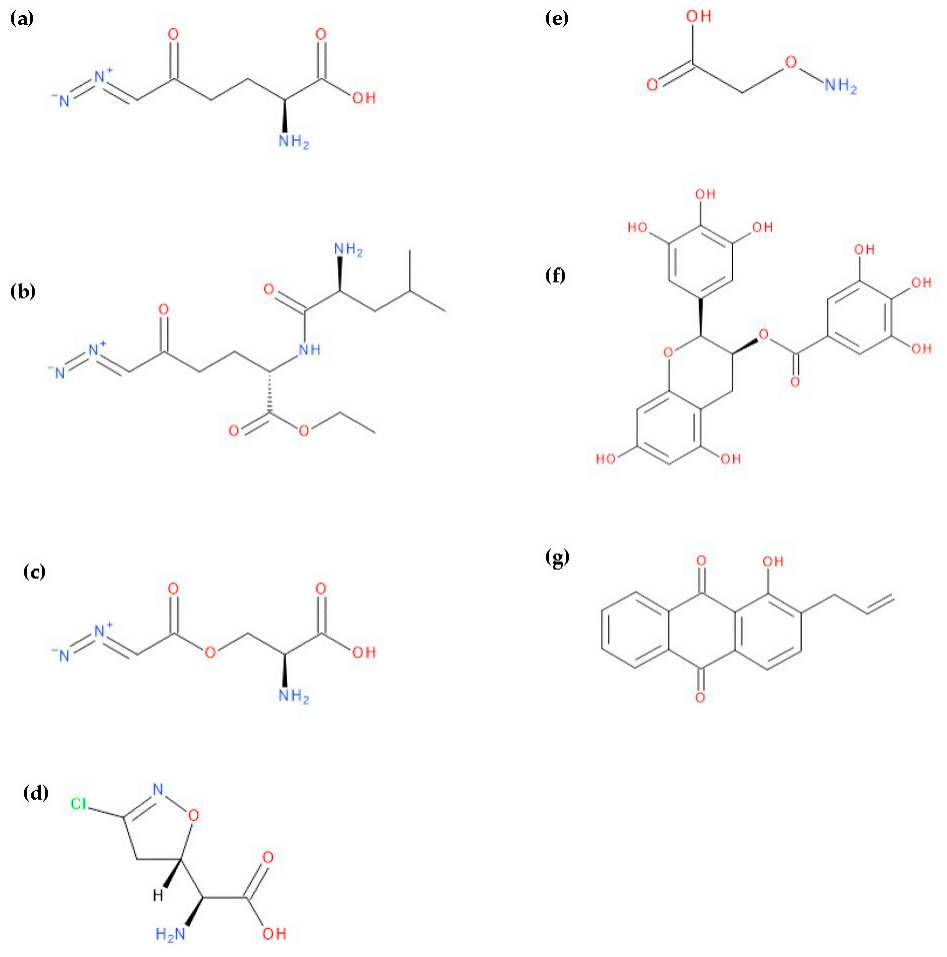
The chemical structures of compounds: (**a**) DON, (**b**) JHU-083, (**c**) azaserine, (**d**) acivicin, (**e**) AOA, (**f**) EGCG, and (**g**) R162.

Other potential therapeutic alternatives are glutamine mimics such as DON (6-diazo-5-oxo-L-norleucine), JHU-083 (ethyl 2-(2-amino-4-methylpentanamido)-DON), azaserine, and acivicin which are limited by their toxicity [10,15,20]. Similarly, the AOA (aminooxyacetic acid) compound, an aminotransferase inhibitor, and L-asparaginase produce glutamine depletion [10]. EGCG (Epigallocatechin gallate) and R162 (2-allyl-1-hydroxy-9,10-anthraquinone) are glutamate dehydrogenase inhibitors that block the transformation of glutamic acid into α-ketoglutarate. For the moment, both of these are considered preclinical compounds [5,10] (Figure 2). 

**Figure 3:**
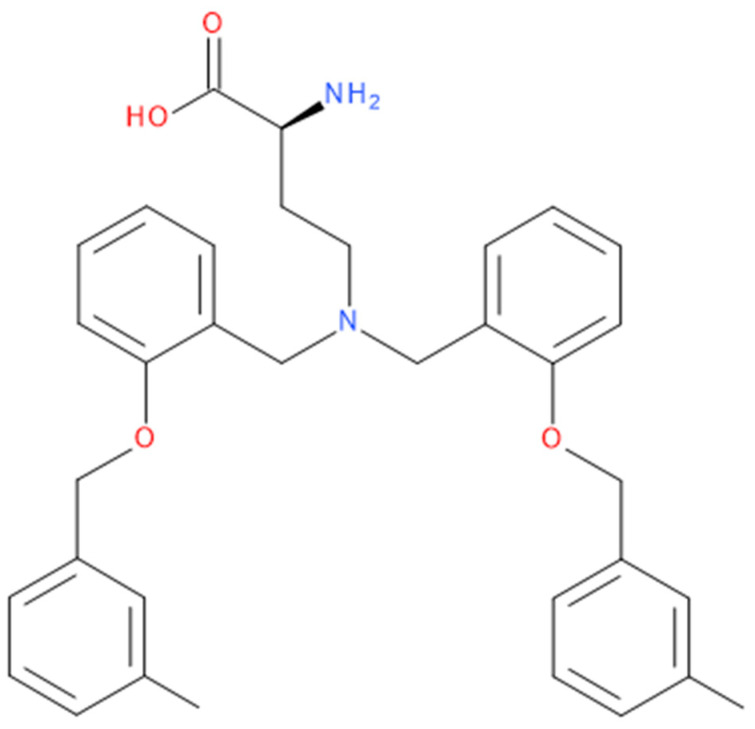
The chemical structure of the ASCT2 inhibitor, V-9302 ((2S)-2-amino-4-[bis[[2-[(3- methylphenyl)methoxy]phenyl]methyl]amino]butanoic acid).

Additional pathways involving amino acid transport suggest effective therapies. Tumour cells achieve high intracellular concentrations of glutamine primarily through the upregulation of glutamine transporters, including ASCT2 (alanine, serine, cysteine transporter 2 or SLC1A5) [5]. Pharmacological blockade of SLC1A5 can be a successful alternative in some types of cancer. V-9302, an SLC1A5 antagonist (Figure 3), elicited a marked anti-tumour response in preclinical tumour models [10,11]. It has blocked glutamine uptake in a broad spectrum of solid tumours (such as colorectal cancer cell lines) and several xenograft tumour models. This blocked glutamine uptake resulted in a profound alteration of tumour cell growth and survival [9,21]. It has been observed that V-9302 was more productive in inducing triple-negative breast cancer cell death in several human and mouse cell culture models [16]. The combination of CB-839 and V-9302 was also successful because of the dual inhibition of glutamine metabolism, resulting in a decrease in GSH levels and a lethal increase in the levels of free radicals. This resulted in severe DNA damage, especially in liver cancer cells [13].

Another therapeutic strategy could be inhibiting glutamate carboxypeptidase II (GCPII). This enzyme hydrolyses N-acetyl-aspartyl-glutamate (NAAG) to glutamate and N-acetyl aspartate. NAAG is a neurotransmitter in the brain and a glutamate provider to GCPII-positive cancers if other sources do not produce enough glutamate. Therefore, inhibitors of GCPII can lead to cancer cell growth suppression by reducing glutamate concentrations [7]. Antagonists of metabotropic glutamate receptors are also promising anti-cancer alternatives without significant side effects. Metabotropic glutamate receptors (mGluRs) are G-protein coupled receptors (GPCRs) categorised into three groups based on their signal transduction pathways and pharmacological profiles. They seem to be more attractive therapeutic targets since they are not directly involved in excitotoxicity but intervene in modulating glutamate activity [22,23].

This article aims to identify new structural analogues of glutamic acid as potential candidates for anti-cancer therapy by computational methods. Several stages were followed: (1) analysis of recently published scientific data regarding the role of glutamate and its derivatives in the development of tumour cells; (2) identification of some new molecules with biological potential, starting with the structure of glutamic acid and the creation of a compound library; (3) conjugation of molecules of natural origin with glutamic acid residues to reduce glutamic acid toxicity and/or potentiate the anti-cancer effect; (4) selection of compounds with biological action and minimal toxicity according to the structural, physicochemical, pharmacokinetic, and pharmaco-toxicological properties determined by in silico methods; (5) evaluation of anti-tumour potential of selected molecules and the identification of possible mechanisms of action; (6) molecular dynamics simulation and molecular docking study to identify the binding site of a ligand molecule (with biological potential) on a known target.

## 2. Results and Discussion

The designed glutamic acid derivatives were classified by classes, groups, and subgroups (Table 1). Each one of the compounds received an ID code composed of the following elements: first digit—class; capital letter—group; small letter—subgroup; last digit—the compound’s number in the subgroup; small letter at the end (if applicable)—a derivative of the lead-compound. The online software and test parameters that were used to obtain and characterise the compounds are mentioned in Table S1 (Supplementary Materials). The structures of all obtained compounds and their computational descriptors are given in Table S2 (Supplementary Materials).

### 2.1. Algorithm for Designing Glutamic Acid Derivatives and Studies Underlying Their Development

The derivatives included in the first two classes were designed based on the specific chemical properties of amino acids resulting from reactions at the carboxyl and amino functional groups. Blocking these essential functional groups in the amino acid’s structure could bring significant changes in terms of its biochemical metabolism; consequently, derivatives with potential pharmaceutical effects are sought [24,25,26,27]. The following classes of compounds comprise structures containing pharmacophores responsible for the anti-cancer effect: thiazole derivatives, 1,3-oxazole derivatives [28,29,30], alkylating agents [28,31,32,33,34,35], inhibitors of histone deacetylase [36,37,38,39], ribonucleotide reductase [40,41,42], glutamate synthetase inhibitors, and mitochondrial transporters of the SLC25A family [43,44,45,46,47,48,49,50,51,52,53,54,55,56,57,58,59,60]. Compounds belonging to these classes have been intensively studied [7,61].

Based on data published about histone deacetylase, compounds from class 4B were designed [39,42,62,63,64,65]. Class 4Cb compounds are based on the structure of Trimidox, an RR inhibitor [40]. Class 4D compounds, inhibitors of GS, and mitochondrial transporters for glutamate are based on Lukasz Berlicki’s (2008) work [66]. The derivative possessing the 4Dd4.m ID code is a metabolite with potential GS inhibitory effect resulting from the hydrolysis of compounds related to tabtoxin (dipeptide) prodrugs: 4Dd4.1, 4Dd4.2 and 4Dd4.3 [67,68,69,70]. Compounds containing sulphur pharmacophores (4Ad1-3, 4Da1-11) are based on the study conducted by Urlich L. (2019) [71].

Based on the information about plant-derived substances with proven anti-cancer effect, we have structurally created compounds of group 5A-E: colchicine derivatives, neferine derivatives, 7-hydroxynuciferine derivatives, lycorine derivatives, 5,6-dehydroglycorine derivatives, and natural compounds conjugated with glutamic acid residues [28,72,73,74,75,76,77,78,79,80,81,82,83,84,85,86,87,88,89]. In addition, the hypothesis that conjugation with a single molecule of glutamic acid could bring benefits compared with the basic compounds of natural origin is being tested through computational studies.

The in silico determination of (1) physicochemical and structural parameters, which implies the determination of heavy atoms (HA), heavy aromatic atoms (HAA), fraction Csp3, rotatable bonds (RB), H-bond acceptors, H-bond donors, molar refractivity (MR), and total polar surface area (TPSA); (2) protonation (acidic pKa, basic pKa, pKa score, isoelectric point (pI), and microspecies); and (3) electric charge (molar polarisability), is detailed in Table S3 (Supplementary Materials). Water solubility was computed using AquaSol [90], Chemicalize [91], and SwissADME [92], and the results are detailed in Table S4 (Supplementary Materials). Lipophilicity and partition coefficients are presented in Table S5 (Supplementary Materials). Toxicity studies were performed in silico by applying the Cramer rules, and the Kroess and Verhaar scheme (Table S6; Supplementary Materials). Other toxicity parameters were also determined, such as carcinogenicity (genotoxic and non-genotoxic) and mutagenicity, skin and eye irritation/corrosion, effect on the reproductive system, biodegradability, and protein and DNA binding alerts, which were evaluated using Toxtree and OSIRIS [93,94]. Results are listed in Tables S7 and S8 (Supplementary Materials).

Pharmacokinetic properties were evaluated for each compound in terms of permeability (gastrointestinal absorption, blood–brain barrier permeability) and interactions with P-gp. In addition, we assessed the enzyme inhibitory effect on some isoforms of cytochrome P450 (Table S9; Supplementary Materials). Based on the previously calculated properties, we evaluated whether these compounds meet the “drug-likeness” criteria according to the Lipinski, Ghose, Veber, Egan, and Muegge rules. The number of rules violated by each molecule is shown in Table S10 (Supplementary Materials), along with the bioavailability score, drug-likeness score, lead-likeness score, and synthetic accessibility score.

Compounds that were too reactive, toxic, or did not have the suitable properties to become lead compounds were removed. Therefore, the screening was performed in several steps according to the rules of Lipinski [91,95,96,97], Veber [95], Ghose [97,98], Egan [99], and Muegge [100] (Table 2), the “overall drug-likeness” score [94,101,102], lead-likeness [103,104], CNSMPO [105], SA [103,106,107,108,109], and by toxicity criteria [93,94,110,111,112,113,114,115,116,117,118,119,120,121,122,123] and pharmacokinetic properties [103,124,125,126,127,128,129,130].

SA is the synthetic accessibility score, which varies from one to ten. It is a parameter used to estimate the ease of synthesising a drug-like molecule: 1 representing being very easy to synthesise and 10 very difficult. This parameter was considered during the abovementioned stages because the subsequent synthesis of the proposed structures will depend heavily on it [92].

### 2.2. The Elimination of Reactive and Toxic Compounds 

The elimination of reactive and toxic compounds was carried out in several steps, as follows:

- Step 1. In the first stage, compounds belonging to at least two toxicity classes are eliminated, as the risk of them causing severe adverse reactions is high.
- Step 2. This step involves the removal of compounds that do not follow Lipinski and Veber’s rules, and which have a CNS MPO score less than 4, as well as compounds with low solubility and/or an inhibitory effect on cytochrome P450 and/or gp-P enzymes.
- Step 3. Compounds with medium toxicity, which fall into Class III (Cramer rules) and are positive for at least one toxicity criterion, are eliminated if the overall drug-likeness score does not exceed 0.90.
- Step 4. Compounds that have violated all Ghose’s rule criteria (four out of four) and belong to Cramer class III or II or overlap with the violation of at least one Muegge rule are eliminated.
- Step 5. Compounds that have violated at least three Ghose criteria and at least two Muegge rules and belong to Cramer class III are eliminated.
- Step 6. Removal of Cramer Class III compounds that violate at least one Ghose and Muegge rule, having an SA score below 2.
- Step 7. Elimination of Class III Cramer compounds that violate at least one Ghose and Muegge rule, regardless of the SA score achieved.
- Step 8. Removal of compounds that violate at least one Ghose and Muegge rule with a low GI absorption value.
- Step 9. Compounds that violate at least one Ghose and Muegge rule with an SA score below 4, regardless of Cramer toxicity class, are eliminated.
- Step 10. Elimination of Cramer Class III compounds that violate at least two Muegge criteria and have an SA score below 3 and/or overall drug-likeness score below 0.5.

Only nine compounds proved to have suitable properties or properties that can be easily optimised, representing 7.3% of the total. These selected compounds are presented in Table 3, along with their geometrical and isomer-conformation properties.

### 2.3. Characterisation of the “Lead” Compounds

The “lead” compounds were characterised by chemical structure, geometric isomers, isomerism, and conformations using the MarvinSketch platform [105] (Table 3). The platform automatically generated the conformations, and their number was limited to ten. The energy was calculated using force field methods, and the conformer with the lowest energy, i.e., having the highest stability, was chosen.

The main pathways of metabolism, bioactivity, action on cancer cells, mechanisms of action and possible adverse effects, and acute toxicity in rodents were further evaluated by in silico methods. For this, we used Toxtree [22] to assess the metabolism of the nine compounds (primary, secondary, tertiary, and quaternary sites of metabolism) and also SmartCyp and SOMP to determine the most reactive atom (involved in interactions with CYP3A4, CYP2D6, and CYP2C9) (Table 4 and Table 5). The algorithm used by the Smartcyp online platform requires a reactivity descriptor (E) and an accessibility descriptor (A). “E” estimates the energy required for a CYP to react at this position, and “A” is the relative topological distance of an atom from the centre of the molecule. The score is calculated for each atom according to the equation Score = E − 8*A − 0.04*SASA (where SASA is the solvent-accessible surface area). A lower score corresponds to an increased probability of being a site of metabolism [131].

The bioactivity of the nine selected compounds was characterised using the following parameters: G protein-coupled receptor ligand, ion channel modulator, kinase inhibitor, nuclear receptor ligand, protease inhibitor, and enzyme inhibitor (Table 6). In addition, the most probable molecular targets and their identification data were determined using the SWISSTarget predictor (Table 7) [133].

Regarding the interpretation of the results from Table 6, a larger score value correlates with a higher probability for the particular molecule to be active. More explicitly, if the bioactivity score is more than 0.0, the compound is considered active; if the score is between −0.5 and 0.0, it exhibits moderate activity; if the bioactivity score is less than −0.5, then it is inactive [134].

The anticarcinogenic effect of the nine compounds was assessed using CLC-Pred software [135], predicting the most probable cell lines for which compounds exhibit cytotoxicity (Table 8). 

Possible mechanisms of action and adverse/toxic effects, lethal doses (LD50) in acute toxicity determined in rodents (intraperitoneal, intravenous, oral, and subcutaneous administration), and the classification of chemical compounds according to the OECD Project were also determined by in silico methods (Table 9, Table 10 and Table 11) [135,136,137,138].

Based on the results of the bioactivity assessment by Molinspiration [134] (Table 6), molecular dynamics and docking studies were performed on compound 4Db6 and the bacterial GS enzyme from *Salmonella typhimurium* (Figure S1; Supplementary Materials) [43,66,139,140,141]. The Protein Data Bank (PDB) code for GS is 1lgr [142,143]. 

The molecular dynamics simulation study was carried out using the UCSF Chimera 1.15 software [144,145]. Before the actual dynamics simulation, the chemical structure was processed according to the protocol established in the literature: hydrogen atoms were inserted, the protonation status corresponding to glutamic acid was used, and Gasteiger partial charges were assigned. The study was performed in water as solvent (SPCBOX, cube size 3 Å) with a density of 1024 g/cm^3^ to simulate physiological conditions. In the neutralisation phase, we added Na/Cl counterions. The next step was the minimisation phase, whereby the system’s energy would tend towards 0.

In the equilibration phase, the temperature was set to 310 K (36.85 ⁰C, approximately physiological temperature) with a gradient of 10 K/ps. In the production phase, the following settings were made: Andersen barostat—pressure 1.0132 bar, relaxation time 1.5; Nose thermostat—emperature 310 K, relaxation time: 0.2. The entire simulation time was set to 100 ns. The energy values resulting from the molecular dynamics simulation for compound 4Db6 are included in Table 12.

Geometry optimisation was performed following the Gaussian model, and we used the standard topology for non-protein molecules. Most biological processes involve, at the atomic scale, the recognition of one molecule by another. Estimation of such interactions at the molecular level is performed by docking methods [146]. In the molecular docking study, the interaction of the 4Db6 derivative with the GS enzyme was evaluated in comparison with phosphinothricin ((2S)-2-amino-4-(hydroxy-methyl-phosphoryl)butanoic acid), whose PDB code is PPQ [67,69,70,142,147]. Phosphinothricin, a GS inhibitor, shows the closest similarity (86.9%) to compound 4Db6, as scored by SwissSimilarity (Score = 0.869) [148]. The comparison was made to identify the most probable binding site in the enzyme structure [149].

**Figure 4:**
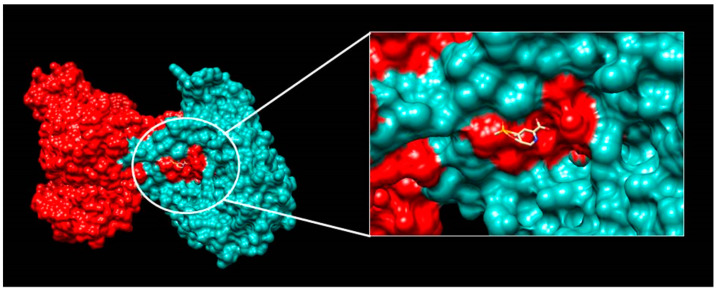
D subunit (in red) and J subunit (blue) of the bacterial GS enzyme and the 4Db6 compound docked at the most probable site estimated by PatchDock [136,153,154]; viewed with UCSF Chimera 1.15 [144,145].

The study was conducted using SwissDock [150,151,152], PatchDock [136,153,154], and AutoDockVina 1.1.2 [151,155]. In a study evaluating a crystalline structure of GS inhibited by phosphinothricin, the inhibitor molecule preferentially binds to the enzyme in the D subunit’s active site. Phosphinothricin occupies the glutamate pocket and stabilises the Glu327 residue in a position that prevents glutamate from entering the active site [149]. This crystal structure (PDB code: 1FPY) was observed using the Mol* Viewer web app of RCSB PDB [142,156]. The preference for the D subunit was also confirmed by results obtained using the PatchDock app, which estimated the most probable binding site for the 4Db6 compound [136,153,154]. The top 10 best solutions are shown in Table 13. Figure 4 illustrates the first best result generated.

However, the selected derivative does not bind to the active site. Thus, these derivatives will probably not show inhibitory activity towards the enzyme. Molecular docking was performed using SwissDock [134,150,152] and AutoDockVina 1.1.2 [151,155] to increase the accuracy of the study.

For PPQ, SwissDock found 257 conformations. The most probable binding site was chosen according to the conformation with the lowest energy, having ΔG = −10.43 kcal/mol and a FullFitness value of −2192.23 kcal/mol [150,152,157]. The FullFitness parameter for a cluster is calculated using the average of 30% of the most favourable energies of its elements to lower the risk of inhibition of the entire cluster by some complexes. This energy is represented by the sum of the system’s total energy and a solvation term [158]. For example, for compound 4Db6, SwissDock found 160 conformations. By comparing the PPQ binding site with the sites of the 160 conformations, we consider that clusters 1, 6 and 33 could bind to the same site in a relatively similar way (Table 14).

**Figure 5:**
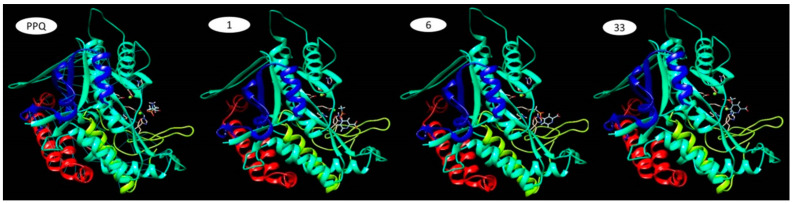
Ligand (PPQ and 4Db6 compound conformers)–receptor (active subunit of GS enzyme) complexes: GS–PPQ; GS–cluster1; GS–cluster6; GS–cluster33. Visualised with UCSF Chimera 1.15 [144,145].

The inhibition constant (Ki) was calculated using the following formula: Ki = e^((ΔG × 1000)/(R × T)), where e = 2.7182, R = 1.98719 cal/(mol × K) (Regnault constant) and T = 298.15 K = 25 °C [159]. It can be seen that cluster 1 shows the lowest energy according to the ΔG value, but Ki and the maximum FullFitness value belong to complex 33. Visualisation and processing of the results obtained in the molecular docking study (Figure 5) were performed using UCSF Chimera 1.15 [144,145]. The grid sizes used in SwissDock for cluster 1 are (x, y, z) = (15.5, 15.5, 20.5) with centre coordinates (x, y, z) = (−98, 13.711, −87.161).

**Figure 6:**
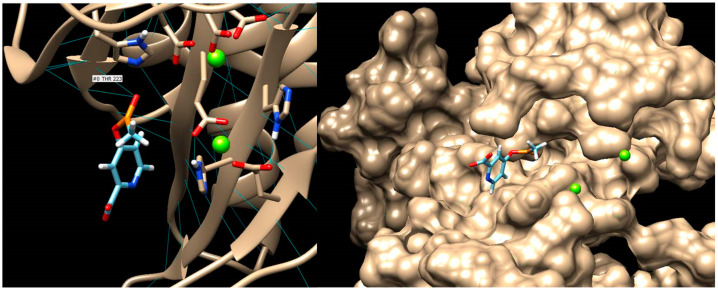
Hydrogen bonds made between the ligand molecule (4Db6 compound) and the threonine residue of the receptor molecule. Visualisation of the ligand inserted into the “binding pocket” [144,145,150,151,152,155,157].

To perform molecular docking using AutoDock Vina (a new version of the Webina online platform), the exhaustiveness of the search was set to 8 and the maximum energy difference to 3 kcal/mol. The space in which the test took place is represented by the volume of a cube (having the following dimensions: width = 20.4346, length = 27.864, height = 18.3759), and whose centre is defined by the coordinates x = −4.86256, y = −15.0503, z = −67.7222) [160]. Preparation for docking involves the insertion of hydrogen atoms on the chemical structure of both the ligand and the receptor molecule and the removal of the solvent. The protonation state corresponding to histidine was used, and Gasteiger partial charges were assigned (Figure 6).

The molecular docking results performed with AutoDock Vina are shown in Table 15, and the corresponding figures are presented in Figure S2 (Supplementary Materials). We chose to work further with model no.1 due to its low free energy (−6.3 kcal/mol) and root-mean-square deviation (RMSD) values that were below 2 Å. The 2 Å limit is often used as a criterion for predicting the correct binding site. The RMSD for two structures, a and b, of an identical molecule can be defined as follows:

RMSD_ab_ = max(RMSD′_ab_, RMSD′_ba_)

(1) $${\mathrm{RMSD}\prime }_{\mathrm{ab}}=\sqrt{\frac{1}{N}\underset{i}{\sum }\begin{array}{c} min\\ j \end{array}r_{\mathrm{ij}\prime }^{2}}$$

where r_ij_ represents the interatomic distance and the sum is over all N HA in structure a; the minimum is over all atoms in structure b with the same element type as the atom in structure a. RMSD is a measure of the distance between experimental and predicted structures that takes into account symmetry, partial symmetry (e.g., within a rotating branch), and near-symmetry [160,161,162,163,164].

The main residues in the D subunit of the GS enzyme involved in interactions (within 1.49–2.81 Å) with the 4Db6 ligand are THR-223 (2 bonds) and GLU-129. Hydrogen bond connections play a key role in determining protein–ligand interactions [160,165]. In addition, the first conformation shows four active torsions: between C4 and P8, CA6 and C7, P8 and C9, and P8 and O11 [160].

## 3. Materials and Methods

Several series of analogous compounds (123 derivatives) have been theoretically designed based on the structure of glutamic acid to build a compound library of glutamic acid derivatives. From simple structure groups to more complex molecules, the chemical structures of the compounds were designed using BIOVIA Draw 21.1. [166]. The number of 123 compounds was reached after analysing the structure of glutamic acid to make as many specific structural modifications as possible. The classes of compounds and the structural changes made to the fundamental molecule were selected following the information found in the scientific literature. Our purpose was initially to design as many structural derivatives as possible because, after characterising and selecting these compounds based on well-established steps, we would be left with as many derivatives with optimal properties as possible to study further.

We also used the same software to generate the computational descriptors. To select suitable candidates for our purpose, we evaluated some properties of the molecules and their behaviour in the human organism. Physico-chemical characterisation of the desired compounds was carried out using SwissADME [92,103] and MarvinSketch [105]. Water solubility was tested using AquaSol [90], Chemicalize [91] and SwissADME [167,168]. Lipophilicity was analysed using SwissADME to determine the partition coefficients [169,170,171,172,173,174]. Toxicity was assessed using Toxtree [93] by applying the Cramer rules and the Kroess and Verhaar scheme, and GUSAR [175] was used to evaluate the acute toxicity in rodents.

Pharmacokinetic properties were analysed in terms of permeability and interactions with P-glycoprotein (P-gp) and some isoforms of cytochrome P450 using the SwissADME program. In addition, we evaluated the “drug-likeness” criteria according to Lipinski, Ghose, Veber, Egan, and Muegge rules using MarvinSketch, Chemicalize and DruLiTo [97].

The metabolism of the compounds was assessed using Toxtree, SmartCyp [131], and SOMP [132] and the bioactivity was evaluated using Molinspiration [134] and SWISSTarget prediction [133] (to predict the most probable molecular targets). The anticarcinogenic effect was assessed with the CLC-Pred software (Version 2.0) [135], which estimates in silico the cytotoxic effect based on the structural formula; the mechanism of action and adverse/toxic effects were tested using PASSonline [137]. 

Molecular docking was performed using SwissDock [150], PatchDock Beta 1.3. [153,176], AutoDockVina 1.1.2. [155], and UCSF Chimera 1.15 [177]; the similarity between compounds was evaluated using SwissSimilarity [178]. We assessed the irritant/corrosive effect on the skin and eyes, the effect on the reproductive system, biodegradability, and protein and DNA binding alerts using Toxtree and OSIRIS Property Explorer [94].

Considering all the computed properties and their biological potential, “lead” compounds were selected.

We also attempted to validate our experimental procedures using positive and negative controls. Therefore, we chose methionine sulfoximine and phosphinothricin as positive controls for their proven activity of inhibiting glutamine synthetase [66,149]. As a negative control, we initially thought of glutamic acid, being the parent molecule for our derivatives [179]. However, it was interesting to observe that, according to the CLC-Pred software, it can show cytotoxic activity on four cell lines [135]. Therefore, in the end, we chose ampicillin as the negative control, which, according to the software, does not show cytotoxicity in any cancer cell line. All compounds were characterized using the previously described platforms and programs, passing through the same steps as the designed glutamic acid derivatives. Molecular docking was assessed using the ProteinsPlus online platform [180]. The results are presented in Tables S13–S18 and Figure S3 (Supplementary Materials).

To increase the accuracy of the study, molecular docking was carried out using several programs since they provided us with different information. PatchDock/ProteinPlus indicated the most probable binding sites in the protein’s structure, calculated the surface area available for ligand binding, and generated the grid-box coordinates. Autodock Vina used these data and refined them, generating the values of ligand affinity for the target molecule and the distance from the RMSD lower bound and RMSD upper bound. It also showed the active torsions between atoms. Finally, SwissDock generated additional information, such as deltaG values and FullFitness, which were used to calculate the inhibition constant Ki.

## 4. Conclusions

Glutamic acid is an amino acid that can be manipulated very easily, and molecules derived from its structure have great potential to become innovative drugs. Of the 123 new GLA derivatives, 9 molecules proved to have biological potential, but more studies and optimisation are needed. The selected compounds show cytotoxicity against breast adenocarcinoma, lung cancer cell lines, colon carcinoma, and T cells from acute leukaemia. Compound 2Ba5 exhibited the lowest toxicity, while derivative 4Db6 exhibited the most intense bioactivity and could act like an ion channel modulator, protease inhibitor or enzyme inhibitor. A molecular docking study determined the binding site of the 4Db6 compound in the GS structure, D subunit, and found cluster 1 to be the most promising, having the lowest free energy value. Since compounds 5Aa1–5Ea3 were eliminated due to their increased toxicity, it is most probable that a single glutamic acid residue bound to the parent molecule cannot reduce the side effects or increase its biological activity. The toxicity of these compounds did not change significantly compared with the parent molecules, except for 7-hydroxynuciferine derivatives, which showed a higher risk of irritation, negative effects on the reproductive system, genotoxic carcinogenicity, tumorigenesis, and a higher risk of mutagenicity compared with 7-hydroxynuciferine. On the other hand, GLA-lycorine and GLA-dehydrolycorine complexes were less irritating to the skin than lycorine and dehydrolycorine, according to data provided by Toxtree and OSIRIS (Table S12; Supplementary Material). Further studies can be performed using these plant-derived molecules combined with more glutamic acid residues or poly-L glutamic acid to obtain more favourable results.

Based on the results provided by Molinspiration and CLC-Pred, further studies can be performed on other enzymes, ion channels, or proteases specific to the colon HCT-116 carcinoma cell line to simulate an interaction with the tumour itself. By marking isotopes at carbon 9 (bonded to the phosphorus atom) in the structure of 4Db6, the molecule can be analysed as a radiopharmaceutical compound (radioligand) as a potential candidate for anti-cancer therapy.

## Figures and Tables

**Table 1:** Classification of derivatives by classes, groups, and subgroups.

Class		Group		Subgroup	
1	Compounds resulting from reactions at the carboxyl group	A	Esters	a	-
		B	Amides	a	-
		C	Acid chlorides	a	-
		D	Anhydrides	a	-
2	Compounds resulting from reactions at the amino group	A	Amides	a	-
		B	Alkylated glutamic acid derivatives	a	Azotyperites
		C	Alcohols resulting from diazotisation	a	-
3	Heterocyclic derivatives	A	Thiazole derivatives	a	Simple
				b	With cyclic anhydride
		B	1,3 Oxazole derivatives	a	Simple
				b	With cyclic anhydride
4	Other derivatives and their potential mechanism of action	A	Alkylating agents	a	Azotyperites
				b	Nitrosoureas
				c	Methylhydrazine
				d	Alkyl sulphonates
				e	Platinum complexes
		B	Histone deacetylase inhibitors	a	-
		C	Ribonucleotide reductase inhibitors	a	Hydroxyurea derivatives
				b	Cyclic compounds (based on the structure of Trimidox)
		D	Inhibitors of glutamate synthetase and/or SLC25A mitochondrial transporters	a	Methionine–sulfoximine analogues
				b	Phosphinothricin analogues
				c	Biphosphonates
				d	Various inhibitors starting from different structures: - d1. 2-Amino-4-hydroxy aminobutyric acid - d2. Alanosine - d3. Oxetine - d4. Tabtoxin and its metabolite (m)
5	Natural substances with proven anti-cancer effects (Table S11; Supplementary Materials) conjugated with glutamic acid molecules	A	Colchicine derivatives	a	Spindle inhibitors
		B	Neferine derivatives	a	-
		C	7-Hydroxycinuciferine derivatives	a	-
		D	Lycorine derivatives	a	-
		E	Derivatives of 5,6-dehydrolycorine	a	-

**Table 2:** The characteristics of Lipinski, Ghose, Veber, Egan, and Muegge drug-likeness rules according to SwissAdme [92].

Drug-Likeness Rules				
Lipinski	Ghose	Veber	Egan	Muegge
MW ≤ 500 Da MlogP ≤ 4.15 N or O ≤ 10 NH or OH ≤ 5	160 ≤ MW ≤ 480 Da −0.4 ≤ WlogP ≤ 5.6 40 ≤ MR ≤ 130 20 ≤ atoms ≤ 70	RB ≤ 10 TPSA ≤ 140	WlogP ≤ 5.88 TPSA ≤ 131.6	200 ≤ MW ≤ 600 Da −2 ≤ XlogP ≤ 5 TPSA ≤ 150 No. of rings ≤ 7 No. of carbon atoms > 4 No. of heteroatoms > 1 No. of RB ≤ 15 H-bond acceptors ≤ 10 H-bond donors ≤ 5

**Table 3:** Structures of the nine “lead” compounds and their geometrical and isomer-conformation properties [105].

No.	ID Code	Chemical Structure	Geometric Isomers		Isomerism		Conformations
			Asymmetric Atoms	Chiral Centres	Tautomers	Stereoisomers	Emin (kcal/mol)
1	1Aa7	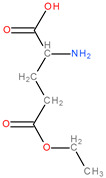	1	1	4	2	10.66
2	1Aa8	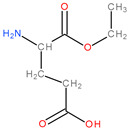	1	1	2	2	10.79
3	2Ba2	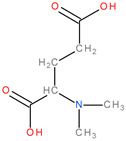	1	1	18	2	12.11
4	2Ba5	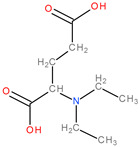	1	1	4	2	26.45
5	2Ba6	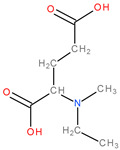	1	1	4	2	25.4
6	3Aa3	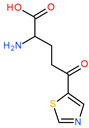	1	1	16	2	31.59
7	3Aa5	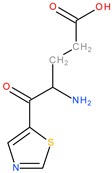	1	1	16	2	31.56
8	4Da11	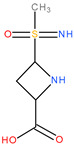	3	3	46	8	73.63
9	4Db6	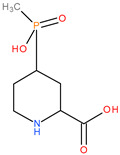	2	2	30	4	62.49

**Table 4:** Compound metabolism assessed using Toxtree [93].

No.	ID Code	Primary Sites of Metabolism	Secondary Sites of Metabolism	Tertiary Sites of Metabolism	Quaternary Sites of Metabolism
1	1Aa7	N-dealkylation	Amine hydroxylation	Aliphatic hydroxylation	O-dealkylation
2	1Aa8	N-dealkylation	Amine hydroxylation	Aliphatic hydroxylation	O-dealkylation
3	2Ba2	N-dealkylation	N-oxidation	N-dealkylation	Aliphatic hydroxylation
4	2Ba5	N-dealkylation	N-dealkylation	N-oxidation	Aliphatic hydroxylation
5	2Ba6	N-dealkylation	None	N-dealkylation	N-oxidation
6	3Aa3	N-dealkylation	Amine hydroxylation	Aromatic hydroxylation	Aliphatic hydroxylation
7	3Aa5	N-dealkylation	Amine hydroxylation	Aliphatic hydroxylation	Aromatic hydroxylation
8	4Da11	N-dealkylation	None	Amine hydroxylation	Aliphatic hydroxylation
9	4Db6	N-dealkylation	None	Amine hydroxylation	Aliphatic hydroxylation

**Table 5:** Compound metabolism assessed using SmartCyp [131] and SOMP [132].

No.	ID Code	3A4		2D6		2C9	
		The Most Reactive Atom	Score	The Most Reactive Atom	Score	The Most Reactive Atom	Score
1	1Aa7	C8	34.7	C1	93.7	C2	86.3
2	1Aa8	C6	36.7	C1	107.1	C2	86.4
3	2Ba2	C1	30.9	C1	85.8	C1	50.7
4	2Ba5	C2	33.2	C2	93.7	C2	57.8
5	2Ba6	C4	32.2	C4	92.7	C4	56.8
6	3Aa3	C2	34.7	C10	74.6	C10	67.9
7	3Aa5	C2	36.8	C13	88	C13	67.9
8	4Da11	C7	35.3	N3	85.8	N3	64.1
9	4Db6	C7	34.5	C7	100.1	C7	75.2

**Table 6:** Bioactivity assessed using Molinspiration [134].

No.	ID Code	GPCR Ligand	Ion Channel Modulator	Kinase Inhibitor	Nuclear Receptor ligand	Protease Inhibitor	Enzyme Inhibitor
1	1Aa7	−0.42	0.17	−1.01	−0.86	−0.2	0.09
2	1Aa8	−0.41	0.13	−1	−0.84	−0.21	0.09
3	2Ba2	−0.11	0.18	−1.01	−0.9	−0.28	0.19
4	2Ba5	−0.02	0.11	−0.89	−0.55	−0.15	0.14
5	2Ba6	−0.02	0.15	−0.96	−0.68	−0.24	0.11
6	3Aa3	−0.1	0.23 *	−0.38	−0.94	0.27 *	0.7 **
7	3Aa5	−0.21	0	−0.28	−0.67	0.33 *	0.43 *
8	4Da11	−0.69	−0.26	−1.36	−0.93	−0.44	0.27 *
9	4Db6	0.12	0.83 **	−0.65	−1.06	0.67 **	0.87 **

* values above 0.2. ** values above 0.5.

**Table 7:** Bioactivity assessed using the SWISSTarget predictor (most probable molecular targets and their identification data) [133].

No.	ID Code	Target	Common Name	Uniprot ID	Target Class	Probability
1	1Aa7	Kynureninase	KYNU	Q16719	Enzyme	0.141787
2	1Aa8	Aminopeptidase A	ENPEP	Q07075	Protease	0.125076
		Kynurenine 3-monooxygenase	KMO	O15229	Oxidoreductase	0.125076
		Glutamate receptor ionotropic, AMPA 1	GRIA1	P42261	Ligand-gated ion channel	0.125076
3	2Ba2	Metabotropic glutamate receptor 3	GRM3	Q14832	Family C G protein-coupled receptor	0.150098
		Metabotropic glutamate receptor 6	GRM6	O15303	Family C G protein-coupled receptor	0.150098
		Metabotropic glutamate receptor 2	GRM2	Q14416	Family C G protein-coupled receptor	0.150098
4	2Ba5	Glutamate receptor ionotropic kainate 1	GRIK1	P39086	Ligand-gated ion channel	0.031227
		Glutamate receptor ionotropic AMPA 1	GRIA1	P42261	Ligand-gated ion channel	0.031227
		Adenosine A3 receptor	ADORA3	P0DMS8	Family A G protein-coupled receptor	0.031227
5	2Ba6	Glutamate receptor ionotropic kainate 1	GRIK1	P39086	Ligand-gated ion channel	0.08057
		Glutamate receptor ionotropic AMPA 1	GRIA1	P42261	Ligand-gated ion channel	0.08057
		Adenosine A3 receptor	ADORA3	P0DMS8	Family A G protein-coupled receptor	0.08057
6	3Aa3	Kynurenine 3-monooxygenase	KMO	O15229	Oxidoreductase	0.04147
		Kynureninase	KYNU	Q16719	Enzyme	0.04147
7	3Aa5	Caspase-3	CASP3	P42574	Protease	0.031227
		Lysine-specific demethylase 2A	KDM2A	Q9Y2K7	Eraser	0.031227
		Histone lysine demethylase PHF8	PHF8	Q9UPP1	Eraser	0.031227
8	4Da11	Fructose-1,6-bisphosphatase	FBP1	P09467	Enzyme	0.053518
		G protein-coupled receptor 44	PTGDR2	Q9Y5Y4	Family A G protein-coupled receptor	0.053518
9	4Db6	Glutamate receptor ionotropic kainate 1	GRIK1	P39086	Ligand-gated ion channel	0.08057
		Glutamate receptor ionotropic AMPA 1	GRIA1	P42261	Ligand-gated ion channel	0.08057
		Glutamate receptor ionotropic kainate 5	GRIK5	Q16478	Ligand-gated ion channel	0.08057

**Table 8:** Anticarcinogenic effect: most probable cell lines for which compounds exhibit cytotoxicity. Probability “to be active” (Pa) > Probability “to be inactive” (Pi) [135,136].

No.	ID Code	Pa	Pi	Cell Line	Cell Line (Full Name)	Tissue	Tumour Type
1	1Aa7	0.694	0.004	NCI-H1299	Non-small cell lung carcinoma	Lung	Carcinoma
2	1Aa8	0.541	0.004	NCI-H1299	Non-small cell lung carcinoma	Lung	Carcinoma
3	2Ba2	0.458	0.023	MDA-MB-453	Breast adenocarcinoma	Breast	Adenocarcinoma
4	2Ba5	0.451	0.008	Jurkat	Acute leukaemia T-cells	Blood	Leukaemia
5	2Ba6	0.438	0.039	MDA-MB-453	Breast adenocarcinoma	Breast	Adenocarcinoma
6	3Aa3	0.717	0.004	DMS-114	Lung carcinoma	Lung	Carcinoma
		0.527	0.005	RKO	Colon carcinoma	Colon	Carcinoma
7	3Aa5	0.728	0.004	DMS-114	Lung carcinoma	Lung	Carcinoma
		0.543	0.005	RKO	Colon carcinoma	Colon	Carcinoma
8	4Da11	0.595	0.01	DMS-114	Lung carcinoma	Lung	Carcinoma
9	4Db6	0.657	0.012	HCT-116	Colon carcinoma	Colon	Carcinoma

**Table 9:** Mechanisms of action and adverse/toxic effects (Pa > Pi) [137].

No.	ID Code	Mechanism of Action			Toxic Effects		
		Pa	Pi	Activity	Pa	Pi	Activity
1	1Aa7	0.965	0.001	Arginine 2-monooxygenase inhibitor	0.982	0.004	Respiratory toxicity
		0.962	0.002	Protein-disulphide reductase (GSH) inhibitor	0.952	0.004	Euphoria
		0.961	0.002	Methylenetetrahydrofolate reductase (NADPH) inhibitor	0.904	0.008	Weakness
		0.952	0.001	Levanase inhibitor	0.892	0.007	Pure red cell aplasia
		0.951	0.002	Acylcarnitine hydrolase inhibitor	0.885	0.007	Muscle weakness
2	1Aa8	0.969	0.001	Protein-disulphide reductase (GSH) inhibitor	0.976	0.005	Toxic, respiratory failure
		0.961	0.002	Methylenetetrahydrofolate reductase (NADPH) inhibitor	0.932	0.005	Euphoria
		0.956	0.001	Arginine 2-monooxygenase inhibitor	0.900	0.004	Apnoea
		0.953	0.001	Levanase inhibitor	0.900	0.008	Weakness
		0.949	0.001	Aspartate kinase inhibitor	0.871	0.009	Neurotoxic
3	2Ba2	0.956	0.001	Methylamine-glutamate N-methyltransferase inhibitor	0.925	0.006	Euphoria
		0.952	0.002	Acylcarnitine hydrolase inhibitor	0.919	0.015	Toxic, respiratory failure
		0.915	0.003	NADPH peroxidase inhibitor	0.870	0.011	Pure red cell aplasia
		0.906	0.004	Anaphylatoxin receptor antagonist	0.860	0.003	Skin irritation, corrosive
		0.906	0.006	Methylenetetrahydrofolate reductase (NADPH) inhibitor	0.851	0.019	Shivering
4	2Ba5	0.945	0.002	Acylcarnitine hydrolase inhibitor	0.958	0.009	Toxic, respiratory failure
		0.941	0.001	Methylamine-glutamate N-methyltransferase inhibitor	0.935	0.005	Euphoria
		0.920	0.002	Dimethylargininase inhibitor	0.920	0.004	Pure red cell aplasia
		0.909	0.002	Aminoacylase inhibitor	0.901	0.006	Shivering
		0.905	0.004	Gluconate 2-dehydrogenase (acceptor) inhibitor	0.888	0.003	Skin irritation, corrosive
5	2Ba6	0.946	0.002	Acylcarnitine hydrolase inhibitor	0.962	0.009	Toxic, respiratory failure
		0.943	0.001	Methylamine-glutamate N-methyltransferase inhibitor	0.954	0.004	Euphoria
		0.900	0.001	Flavin-containing monooxygenase inhibitor	0.918	0.002	Skin irritation, corrosive
		0.889	0.007	Phobic disorders treatment	0.894	0.007	Pure red cell aplasia
		0.884	0.003	Dimethylargininase inhibitor	0.876	0.006	Postural (orthostatic) hypotension
6	3Aa3	0.866	0.003	Glutamine-phenylpyruvate transaminase inhibitor	0.766	0.020	Respiratory failure
		0.853	0.005	Monodehydroascorbate reductase (NADH) inhibitor	0.731	0.035	Ulcer, aphthous
		0.800	0.009	Arginine 2-monooxygenase inhibitor	0.686	0.009	Anaemia, sideroblastic
		0.803	0.018	Methylenetetrahydrofolate reductase (NADPH) inhibitor	0.707	0.041	Pure red cell aplasia
		0.793	0.013	NADPH peroxidase inhibitor	0.667	0.033	Stomatitis
7	3Aa5	0.797	0.014	Acylcarnitine hydrolase inhibitor	0.764	0.022	Stomatitis
		0.787	0.005	Glutamine-phenylpyruvate transaminase inhibitor	0.719	0.026	Respiratory failure
		0.794	0.019	Methylenetetrahydrofolate reductase (NADPH) inhibitor	0.702	0.020	Asthma
		0.734	0.002	Pyrimidine-deoxynucleoside 2′-dioxygenase inhibitor	0.689	0.015	Respiratory impairment
		0.736	0.021	NADPH peroxidase inhibitor	0.655	0.020	Haematuria
8	4Da11	0.932	0.004	Angiogenesis inhibitor	0.496	0.074	Haematemesis
		0.930	0.004	Anti-inflammatory	0.439	0.038	Thrombocytopoiesis inhibitor
		0.923	0.004	Glutamate-5-semialdehyde dehydrogenase inhibitor	0.436	0.078	Interstitial nephritis
		0.869	0.001	CDK1/cyclin B inhibitor	0.463	0.109	Occult bleeding
		0.865	0.002	Macular degeneration treatment	0.450	0.105	Nephritis
9	4Db6	0.957	0.002	Glutamate-5-semialdehyde dehydrogenase inhibitor	0.651	0.023	Ototoxicity
		0.952	0.000	Sphingosine 1-phosphate receptor 5 antagonist	0.520	0.069	Bronchoconstriction
		0.793	0.002	GABA C receptor antagonist	0.343	0.158	Sneezing
		0.782	0.003	Ornithine cyclodeaminase inhibitor	0.280	0.097	Demyelination
		0.701	0.003	Bone formation stimulant	0.319	0.159	Fibrosis, interstitial

**Table 10:** Acute toxicity in rodents when administered intraperitoneally, intravenously, orally, and subcutaneously: LD50 in mg/kg [138].

No.	ID Code	Rat IP LD50 (mg/kg)	Rat IV LD50 (mg/kg)	Rat Oral LD50 (mg/kg)	Rat SC LD50 (mg/kg)
1	1Aa7	2593.000 in AD	1256.000 in AD	5859.000 in AD	6254.000 in AD
2	1Aa8	3059.000 in AD	1268.000 in AD	4228.000 in AD	4014.000 in AD
3	2Ba2	1069.000 in AD	1017.000 in AD	1978.000 in AD	1027.000 in AD
4	2Ba5	436.000 in AD	865.000 in AD	1861.000 in AD	1026.000 out of AD
5	2Ba6	375.200 in AD	613.100 in AD	1198.000 in AD	505.500 in AD
6	3Aa3	418.900 in AD	643.600 in AD	3172.000 in AD	2290.000 in AD
7	3Aa5	585.600 in AD	464.800 in AD	2623.000 out of AD	1923.000 in AD
8	4Da11	551.700 out of AD	580.800 in AD	3362.000 in AD	298.500 in AD
9	4Db6	298.100 out of AD	180.400 in AD	1456.000 out of AD	76.460 in AD

**Table 11:** Acute toxicity in rodents. Classification of Chemicals according to the OECD Project [138].

No.	ID Code	Rat IP LD50 Classification	Rat IV LD50 Classification	Rat Oral LD50 Classification	Rat SC LD50 Classification
1	1Aa7	Non-Toxic in AD	Non-Toxic in AD	Non-Toxic in AD	Non-Toxic in AD
2	1Aa8	Non-Toxic in AD	Non-Toxic in AD	Class 5 in AD	Non-Toxic in AD
3	2Ba2	Class 5 in AD	Non-Toxic in AD	Class 4 in AD	Class 5 in AD
4	2Ba5	Class 4 in AD	Non-Toxic in AD	Class 4 in AD	Class 5 out of AD
5	2Ba6	Class 4 in AD	Class 5 in AD	Class 4 in AD	Class 4 in AD
6	3Aa3	Class 4 in AD	Class 5 in AD	Class 5 in AD	Class 5 in AD
7	3Aa5	Class 5 in AD	Class 5 in AD	Class 5 out of AD	Class 5 in AD
8	4Da11	Class 5 out of AD	Class 5 in AD	Class 5 in AD	Class 4 in AD
9	4Db6	Class 4 out of AD	Class 4 in AD	Class 4 out of AD	Class 3 in AD

**Table 12:** Molecular dynamics simulation results for compound 4Db6 [144].

Step	Time (fs)	Potential Energy (J)	Kinetic Energy (J)
0	0.0	341.630730	86.279577
100	0.1	335.578989	92.292502
200	0.2	351.037095	77.385248
300	0.3	333.800802	94.478719
400	0.4	353.520040	74.902008
500	0.5	363.225563	65.233746
600	0.6	365.055252	63.321001
700	0.7	359.244207	69.127817
800	0.8	333.010201	95.326086
900	0.9	336.650457	91.597157
1000	1	362.614614	65.517278

**Table 13:** Molecular docking results for the 4Db6 compound using PatchDock [136,153,154].

No.	Score	Interface Area	Coordinates
1	2900	318.4	−1.34; −0.09; 1.38; −23.80; −22.56; −39.82
2	2858	318.3	1.02; 0.08; 0.88; −48.08; 21.92; −56.70
3	2834	310.4	−1.95; 0.16; −1.70; −74.21; −8.23; −46.64
4	2830	306	1.32; 0.01; −2.83; −44.00; −28.47; −52.58
5	2814	318.5	−1.33; −0.27; 1.59; −27.20; −37.47; −60.51
6	2792	316.6	−1.14; −0.17; −1.48; −66.81; 35.58; −67.43
7	2792	308.1	−1.78; −0.03; 2.84; −41.83; 28.83; −45.53
8	2790	301.5	−1.64; 0.44; 1.33; −71.19; −15.76; −81.43
9	2786	310.8	2.09; −0.02; −1.89; −16.57; 19.50; −51.15
10	2786	296.7	−2.08; 0.27; −1.76; −57.26; −20.74; −68.35

**Table 14:** Energetic values of the most probable ligand (4Db6 compound)–receptor complexes [144,150,152,157].

Cluster	ΔG (kcal/mol)	FullFitness (kcal/mol)	Ki
1	−8.1	−2139.9	11.264 × 10^−7^
6	−7.6	−2137.1	22.904 × 10^−7^
33	−6.8	−2126.6	94.047 × 10^−7^

**Table 15:** Molecular docking results for the 4Db6 compound obtained using AutoDock Vina. Run time: 28.3 s.

Mode	Affinity (kcal/mol)	Dist. from RMSD L. B	Dist. from RMSD U. B
1	−6.3	0	0
2	−6	1.805	4.092
3	−5.7	2.296	2.819
4	−5.3	4.405	5.497
5	−5.3	9.763	11.591
6	−5.3	2.674	3.792
7	−5.3	3.029	5.193
8	−5.2	2.142	2.893
9	−5.1	2.042	2.793

## Data Availability

Not applicable.

## Supplementary Materials

The following supporting information can be downloaded at: https://www.mdpi.com/article/10.3390/molecules28104123/s1, Table S1: Programs and tested parameters; Table S2: Chemical structures, ID codes, and computational descriptors of glutamic acid derivatives obtained with Biovia Draw; Table S3: Structural and physicochemical properties: protonation and electric charge; Table S4: Water solubility; Table S5: Lipophilicity—partition coefficients; Table S6: Toxicity—I: Cramer rules, Kroess and Verhaar scheme; Table S7: Toxicity—II. Carcinogenic (genotoxic and non-genotoxic) and mutagenic effects evaluated using two different apps (Toxtree and OSIRIS); Table S8: Toxicity—III. Irritant/corrosive effect on the skin and eyes, effect on the reproductive system, biodegradability, and protein and DNA binding alerts, as assessed using Toxtree and OSIRIS; Table S9: Permeability and interactions with P-gp. Enzyme inhibitory effect on isoforms of cytochrome P450; Table S10: The number of broken rules, according to Lipinski, Ghose, Veber, Egan, and Muegge and the bioavailability score, the drug-likeness score, the lead-likeness score and the synthetic accessibility score; Figure S1: Homododecameric structure of the bacterial GS enzyme and D subunit; Figure S2: Molecular docking results visualised using UCSF Chimera and AutoDock Vina (Webina); Table S11: Chemical structures of colchicine, neferine, 7-hydroxynuciferine, lycorine, and 5,6-dehydrolycorine; Table S12: Toxicity comparison of vegetal compounds and their complexes with glutamic acid; Table S13: Characterization of phosphinothricin, methionine sulfoximine, glutamic acid, and ampicillin; Table S14: Molecular dynamics simulation results for phosphinothricin, methionine sulfoximine, glutamic acid, and ampicillin; Table S15: Molecular docking results for phosphinothricin, methionine sulfoximine, glutamic acid, and ampicillin; Figure S3: Molecular docking results. Interaction with glutamine synthetase of (a) hosphinothricin, (b) methionine sulfoximine, (c) glutamic acid, and (d) ampicillin; Table S16: Grid sizes used in Swissdock and energetic values of the most probable ligand–receptor complexes for phosphinothricin, methionine sulfoximine, glutamic acid, and ampicillin; Table S17: Molecular docking results obtained using AutoDock Vina for phosphinothricin, methionine sulfoximine, glutamic acid, and ampicillin; Table S18: Grid sizes used in AutoDock Vina for phosphinothricin, methionine sulfoximine, glutamic acid and ampicillin.

## Abbreviations

ADME	Absorption, Distribution, Metabolism and Excretion
HAA	Heavy aromatic atoms
BBB	Blood–Brain Barrier
BD	Bioavailability
CLC-Pred	Cell Line Cytotoxicity Predictor
CNS MPO	Central Nervous System Multiparameter Optimisation
ESOL	Estimating Aqueous Solubility Directly from Molecular Structure
GS	Glutamine synthetase
GSH	Glutathione
GPCR	G-protein coupled receptor
GPL	General Public License
GUSAR	General Unrestricted Structure–Activity Relationships
HA	Heavy atoms
HLB	Hydrophilic Lipophilic Balance
Ki	Inhibition constant
LD50	Lethal dose 50
MR	Molar refractivity
PDB	Protein Data Bank
P-gp	P-glycoprotein
pI	Isoelectric point
QSAR	Quantitative Structure–Activity Relationships
QSPR	Quantitative Structure–Property Relationships
RB	Rotatable bonds
SA	Synthetic accessibility score
SLC25	The solute carrier family 25
SN1	Nucleophilic substitution type 1
SN2	Aliphatic nucleophilic substitution type 2
SOMP	Site of Metabolism Prediction
TPSA	Total polar surface area
TTC	Threshold of Toxicological Concern
