# Supplementary material for: Identification of Some Glutamic Acid Derivatives with Biological Potential by Computational Methods
Source: Molecules. 2023 May 16;28(10):4123. doi: 10.3390/molecules28104123 (PMC10224408; doi:10.3390/molecules28104123)
Supplement: Supplementary file 1 [file molecules-28-04123-s001.zip › molecules-2321460-supplementary.pdf]

IDENTIFICATION OF SOME GLUTAMIC ACID DERIVATIVES WITH BIOLOGICAL POTENTIAL BY COMPUTATIONAL METHODS

OCTAVIA-LAURA MOLDOVAN[1,†,*], ALEXANDRA SANDULEA[2,†], IOANA-ANDREEA LUNGU[1], ȘERBAN ANDREI GÂZ [3] AND AURA RUSU[2]

[1] *Medicine and Pharmacy Doctoral School, George Emil Palade University of Medicine, Pharmacy, Science and Technology of Targu Mures, 540142, Targu Mures, Romania*
E-mail: octavia.moldovan@umfst.ro , ioana-andreea.lungu@umfst.ro

[2] *Pharmaceutical and Therapeutic Chemistry Department, Faculty of Pharmacy, George Emil Palade University of Medicine, Pharmacy, Science and Technology of Targu Mures, 540142, Targu Mures, Romania*
E-mail: sandulea.alex@yahoo.com , aura.rusu@umfst.ro

[3] *Organic Chemistry Department, George Emil Palade University of Medicine, Pharmacy, Science and Technology of Targu Mures, 540142, Targu Mures, Romania*
E-mail: andrei.gaz-florea@umfst.ro

[†] *These authors share the first authorship.*

* *Corresponding author email: octavia.moldovan@umfst.ro*

SUPPLEMENTARY MATERIALS

Table S1. Programs and tested parameters.

No.	Program name	Tested parameters	References
1	BIOVIA Draw	With the help of this application, the chemical structures were created, and the computational descriptors were generated:<br>- IUPAC name<br>-Molecular weight and formula<br>-InChI String and InChI Key<br>-SMILES	[1]
2	MarvinSketch	-Protonation: pKa, microspecies, pI<br>-Partition coefficients: logP, logD, HLB<br>-Electric charge: polarizability<br>-Isomery: tautomers, stereoisomers<br>-Conformation: conformers<br>-Geometry: number of asymmetric atoms, polar surface area<br>-Other: hydrogen donors-acceptors, CNS MPO score	[2]
3	AquaSol	logS	[3]
4	Chemicalize	-Lipinski's rule<br>-Solubility class	[4]
5	Toxtree	-Cramer rule, Kroes TTC, Verhaar scheme<br>-Carcinogenesis (genotoxic-non-genotoxic) and mutagenesis<br>- *In vitro* mutagenesis (Ames test)<br>-Skin irritation<br>-Biodegradability<br>-Metabolism mediated by cytochrome P450<br>-Protein and DNA binding alerts	[5]
6	OSIRIS Property Explorer	- Toxicity risk: mutagenesis, tumorigenesis, irritating effect, adverse effects on the reproductive system<br>-Drug-likeness<br>-Overall drug-likeness score	[6]
7	DruLiTo	-Lipinski's rule<br>-BBB rule	[7]
8	SMARTCyp	Metabolism by isoforms 3A4, 2D6 and 2C9	[8]
9	Molinspiration	Bioactivity:<br>-GPCR ligand<br>-Ion channel modulator<br>-Kinase inhibitor	[9]

No.	Program name	Tested parameters	References
		-Ligand on nuclear receptors<br>-Protease inhibitor<br>-Enzyme inhibitor	
10	SwissADME	-HA, HAA<br>-Csp3 fraction<br>-RB<br>-Acceptor and donor hydrogen bonds<br>-MR<br>-TPSA<br>-iLOGP, XLOGP4, WLOGP, MLOGP, Silicos-IT LogP<br>-Consensus LogP<br>-ESOL LogS<br>-ESOL Solubility (mg/ml and mol/L)<br>-ESOL Class<br>-Ali Log S<br>-Ali Solubility (mg/ml and mol/L)<br>-Ali Class<br>-Silicos-IT Log Sw<br>-Silicos-IT Solubility (mg/ml and mol/L)<br>-Silicos-IT Class<br>-GI Absorption<br>-BBB Permeability<br>-P-gp Substrate<br>-CYP1A2, CYP2C19, CYP2C9 , CYP2D6, CYP3A4 inhibitor<br>-log Kp (permeability)<br>-Lipinski, Ghose, Veber, Egan, Muegge<br>-BD Score, Leadlikeness, SA	[10,11]
11	SwissTarget Prediction	Molecular targets and the probability of binding the compound to that target	[12]
12	SwissDock	Saving the target receptor structures in PDB format, necessary for molecular docking	[13]
13	CLC-Pred	The type of cancerous tissue and cell line for which the compound could have a cytotoxic effect, proved that Pa (probability of „being active”) is higher than Pi (probability of „being inactive”).	[14]
14	GUSAR	Acute toxicity in rodents: LD50 values (log10 (mg/kg)) for oral, intravenous, intraperitoneal and subcutaneous administration.	[15]
15	SOMP	No.of the atom most likely to be metabolized by CYP1A2, CYP2C9, CYP2C19, CYP2D6 and CYP3A4, according to the value of the DeltaP parameter	[16]
16	PASSonline	-Mechanisms of action	[17]

No.	Program name	Tested parameters	References
		-Adverse/toxic effects<br>(condition: Pa>Pi)	
17	AutoDock Vina 1.1.2	Molecular docking (the program was used as an extension within Chimera software)	[18]
18	UCSF Chimera 1.15	-Molecular docking<br>-Molecular dynamics simulation	[19]
19	PatchDock Beta 1.3	The most likely sites of interaction for a given receptor molecule, presented as coordinates. The data obtained were later used in the Chimera program.	[20,21]
20	SwissSimilarity	Compounds that show structural similarity to glutamic acid derivatives, along with the similarity score	[22]

Table S2. Chemical structures, ID codes and computational descriptors of glutamic acid derivatives, obtained with BioviaDraw.

No.	ID code	Structural formula	SMILES	Molecular weight	Molecular formula	IUPAC name	InChI String	InChI Key
1	1Aa1	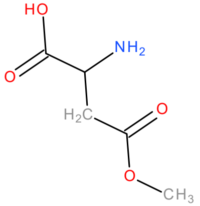	COC(=O)CC(N)C(O)=O	147.13	$C_5H_9NO_4$	2-amino-4-methoxy-4-oxobutanoic acid	InChI=1S/C5H9NO4/c1-10-4(7)2-3(6)5(8)9/h3H,2,6H2,1H3,(H,8,9)	SBRYFUVVWOMLLP-UHFFFAOYSA-N
2	1Aa2	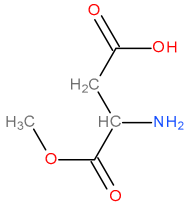	COC(=O)C(N)CC(O)=O	147.13	$C_5H_9NO_4$	3-amino-4-methoxy-4-oxobutanoic acid	InChI=1S/C5H9NO4/c1-10-5(9)3(6)2-4(7)8/h3H,2,6H2,1H3,(H,7,8)	SWWBMHIMADRNIK-UHFFFAOYSA-N
3	1Aa3	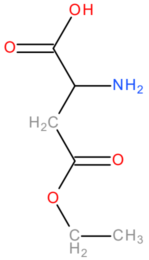	CCOC(=O)CC(N)C(O)=O	161.157	$C_6H_{11}NO_4$	2-amino-4-ethoxy-4-oxobutanoic acid	InChI=1S/C6H11NO4/c1-2-11-5(8)3-4(7)6(9)10/h4H,2-3,7H2,1H3,(H,9,10)	KALSWOYBXAHEKF-UHFFFAOYSA-N
4	1Aa4	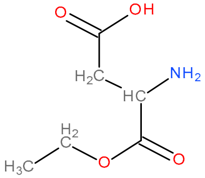	CCOC(=O)C(N)CC(O)=O	161.157	$C_6H_{11}NO_4$	3-amino-4-ethoxy-4-oxobutanoic acid	InChI=1S/C6H11NO4/c1-2-11-6(10)4(7)3-5(8)9/h4H,2-3,7H2,1H3,(H,8,9)	WXFCDLWCQIARFW-UHFFFAOYSA-N

No.	ID code	Structural formula	SMILES	Molecular weight	Molecular formula	IUPAC name	InChI String	InChI Key
5	1Aa5	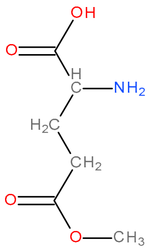	COC(=O)CCC(N)C(O)=O	161.157	$C_6H_{11}NO_4$	2-amino-5-methoxy-5-oxopentanoic acid	InChI=1S/C6H11NO4/c1-11-5(8)3-2-4(7)6(9)10/h4H,2-3,7H2,1H3,(H,9,10)	ZGEYCCHDTIDZAE-UHFFFAOYSA-N
6	1Aa6	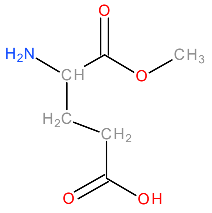	COC(=O)C(N)CCC(O)=O	162.157	$C_6H_{11}NO_5$	4-amino-5-methoxy-5-oxopentanoic acid	InChI=1S/C6H11NO4/c1-11-6(10)4(7)2-3-5(8)9/h4H,2-3,7H2,1H3,(H,8,9)	SEWIYICDCVPBEW-UHFFFAOYSA-N
7	1Aa7	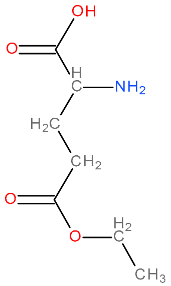	CCOC(=O)CCC(N)C(O)=O	175.184	$C_7H_{13}NO_4$	2-amino-5-ethoxy-5-oxopentanoic acid	InChI=1S/C7H13NO4/c1-2-12-6(9)4-3-5(8)7(10)11/h5H,2-4,8H2,1H3,(H,10,11)	XMQUEQJCYRFIQS-UHFFFAOYSA-N
8	1Aa8	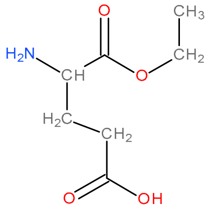	CCOC(=O)C(N)CCC(O)=O	175.184	$C_7H_{13}NO_4$	4-amino-5-ethoxy-5-oxopentanoic acid	InChI=1S/C7H13NO4/c1-2-12-7(11)5(8)3-4-6(9)10/h5H,2-4,8H2,1H3,(H,9,10)	SYQNQPHXKWWZFS-UHFFFAOYSA-N

No.	ID code	Structural formula	SMILES	Molecular weight	Molecular formula	IUPAC name	InChI String	InChI Key
9	1Ba1	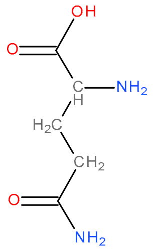	NC(CCC(N)=O)C(O)=O	146.146	$C_5H_{10}N_2O_3$	2-amino-4-carbamoylbutanoic acid	InChI=1S/C5H10N2O3/c6-3(5(9)10)1-2-4(7)8/h3H,1-2,6H2,(H2,7,8)(H,9,10)	ZDXPYRJPNDTMRX-UHFFFAOYSA-N
10	1Ba2	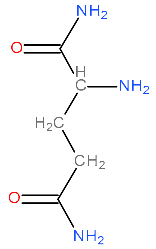	NC(CCC(N)=O)C(N)=O	145.162	$C_5H_{11}N_3O_2$	2-aminopentanediamide	InChI=1S/C5H11N3O2/c6-3(5(8)10)1-2-4(7)9/h3H,1-2,6H2,(H2,7,9)(H2,8,10)	LCGISIDBXHGCDW-UHFFFAOYSA-N
11	1Ba3	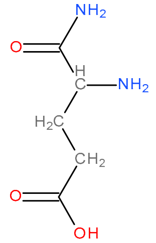	NC(CCC(O)=O)C(N)=O	146.146	$C_5H_{10}N_2O_3$	4-amino-4-carbamoylbutanoic acid	InChI=1S/C5H10N2O3/c6-3(5(7)10)1-2-4(8)9/h3H,1-2,6H2,(H2,7,10)(H,8,9)	AEFLONBTGZFSGQ-UHFFFAOYSA-N
12	1Ca1	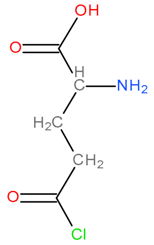	NC(CCC(Cl)=O)C(O)=O	165.57	$C_5H_8ClNO_3$	2-amino-5-chloro-5-oxopentanoic acid	InChI=1S/C5H8ClNO3/c6-4(8)2-1-3(7)5(9)10/h3H,1-2,7H2,(H,9,10)	FTDIKXWDDQYEGB-UHFFFAOYSA-N

No.	ID code	Structural formula	SMILES	Molecular weight	Molecular formula	IUPAC name	InChI String	InChI Key
13	1Ca2	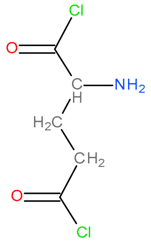	NC(CCC(Cl)=O)C(Cl)=O	184.02	$C_5H_7Cl_2NO_2$	2-aminopentanedioyl dichloride	InChI=1S/C5H7Cl2NO2/c6-4(9)2-1-3(8)5(7)10/h3H,1-2,8H2	BGQUGWDFOLIPAH-UHFFFAOYSA-N
14	1Ca3	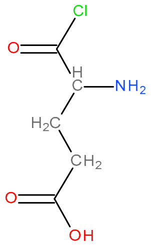	NC(CCC(O)=O)C(Cl)=O	165.57	$C_5H_8ClNO_3$	4-amino-5-chloro-5-oxopentanoic acid	InChI=1S/C5H8ClNO3/c6-5(10)3(7)1-2-4(8)9/h3H,1-2,7H2,(H,8,9)	MNCCGMMMOFFNMM-UHFFFAOYSA-N
15	1Da1	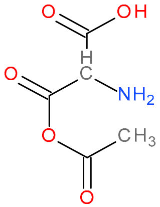	CC(=O)OC(=O)C(N)C(O)=O	161.113	$C_5H_7NO_5$	3-(acetyloxy)-2-amino-3-oxopropanoic acid	InChI=1S/C5H7NO5/c1-2(7)11-5(10)3(6)4(8)9/h3H,6H2,1H3,(H,8,9)	MSDXYRKPRIIYJT-UHFFFAOYSA-N
16	1Da2	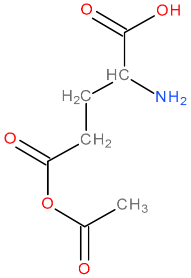	CC(=O)OC(=O)CCC(N)C(O)=O	189.167	$C_7H_{11}NO_5$	5-(acetyloxy)-2-amino-5-oxopentanoic acid	InChI=1S/C7H11NO5/c1-4(9)13-6(10)3-2-5(8)7(11)12/h5H,2-3,8H2,1H3,(H,11,12)	FBCSEFYXPQJLFW-UHFFFAOYSA-N

No.	ID code	Structural formula	SMILES	Molecular weight	Molecular formula	IUPAC name	InChI String	InChI Key
17	1Da3	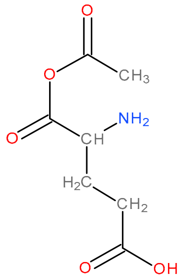	CC(=O)OC(=O)C(N)CCC(O)=O	189.167	$C_7H_{11}NO_5$	5-(acetyloxy)-4-amino-5-oxopentanoic acid	InChI=1S/C7H11NO5/c1-4(9)13-7(12)5(8)2-3-6(10)11/h5H,2-3,8H2,1H3,(H,10,11)	PUTYUEPGBGUQNX-UHFFFAOYSA-N
18	1Da4	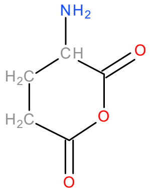	NC1CCC(=O)OC1=O	129.115	$C_5H_7NO_3$	3-aminooxane-2,6-dione	InChI=1S/C5H7NO3/c6-3-1-2-4(7)9-5(3)8/h3H,1-2,6H2	RFWFOJDAIRDAPK-UHFFFAOYSA-N
19	2Aa1	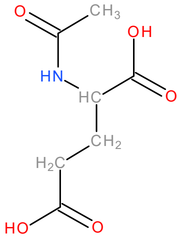	CC(=O)NC(CCC(O)=O)C(O)=O	189.167	$C_7H_{11}NO_5$	2-acetamidopentanedioic acid	InChI=1S/C7H11NO5/c1-4(9)8-5(7(12)13)2-3-6(10)11/h5H,2-3H2,1H3,(H,8,9)(H,10,11)(H,12,13)	RFMMMVDNIPUKGG-UHFFFAOYSA-N
20	2Ba1	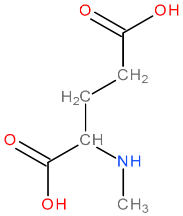	CNC(CCC(O)=O)C(O)=O	161.157	$C_6H_{11}NO_4$	2-(methylamino)pentanedioic acid	InChI=1S/C6H11NO4/c1-7-4(6(10)11)2-3-5(8)9/h4,7H,2-3H2,1H3,(H,8,9)(H,10,11)	MOSFIJXAXDLOML-UHFFFAOYSA-N

No.	ID code	Structural formula	SMILES	Molecular weight	Molecular formula	IUPAC name	InChI String	InChI Key
21	2Ba2	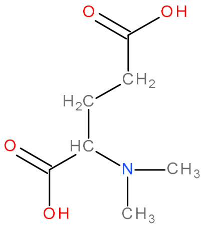	CN(C)C(CCC(O)=O)C(O)=O	175.184	$C_7H_{13}NO_4$	2-(dimethylamino)pentanedioic acid	InChI=1S/C7H13NO4/c1-8(2)5(7(11)12)3-4-6(9)10/h5H,3-4H2,1-2H3,(H,9,10)(H,11,12)	JCHFXGIXHDRBFU-UHFFFAOYSA-N
22	2Ba3	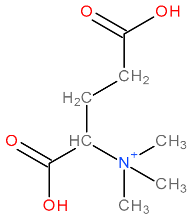	C[N+](C)(C)C(CCC(O)=O)C(O)=O	190.218	$C_8H_{16}NO_4$	(1,3-dicarboxypropyl)trimethylazanium	InChI=1S/C8H15NO4/c1-9(2,3)6(8(12)13)4-5-7(10)11/h6H,4-5H2,1-3H3,(H-,10,11,12,13)/p+1	WTIGXLNNRAGPIV-UHFFFAOYSA-O
23	2Ba4	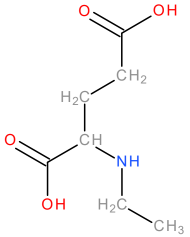	CCNC(CCC(O)=O)C(O)=O	175.184	$C_7H_{13}NO_4$	2-(ethylamino)pentanedioic acid	InChI=1S/C7H13NO4/c1-2-8-5(7(11)12)3-4-6(9)10/h5,8H,2-4H2,1H3,(H,9,10)(H,11,12)	BIAZEQPYCCADLD-UHFFFAOYSA-N
24	2Ba5	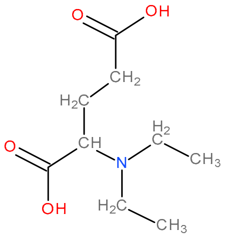	CCN(CC)C(CCC(O)=O)C(O)=O	203.238	$C_9H_{17}NO_4$	2-(diethylamino)pentanedioic acid	InChI=1S/C9H17NO4/c1-3-10(4-2)7(9(13)14)5-6-8(11)12/h7H,3-6H2,1-2H3,(H,11,12)(H,13,14)	NXWYAHLLQFLYDY-UHFFFAOYSA-N

No.	ID code	Structural formula	SMILES	Molecular weight	Molecular formula	IUPAC name	InChI String	InChI Key
25	2Ba6	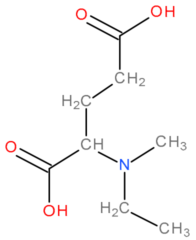	CCN(C)C(CCC(O)=O)C(O)=O	189.211	$C_8H_{15}NO_4$	2-[ethyl(methyl)amino]pentanedioic acid	InChI=1S/C8H15NO4/c1-3-9(2)6(8(12)13)4-5-7(10)11/h6H,3-5H2,1-2H3,(H,10,11)(H,12,13)	RTJZKUDFMSLYMP-UHFFFAOYSA-N
26	2Ba6a	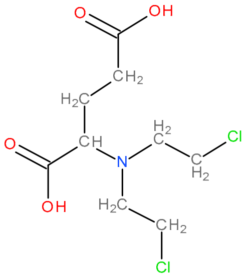	OC(=O)CCC(N(CCCl)CCCl)C(O)=O	272.12	$C_9H_{15}Cl_2NO_4$	2-[bis(2-chloroethyl)amino]pentanedioic acid	InChI=1S/C9H15Cl2NO4/c10-3-5-12(6-4-11)7(9(15)16)1-2-8(13)14/h7H,1-6H2,(H,13,14)(H,15,16)	GPSOFGZZMYRTRE-UHFFFAOYSA-N
27	2Ca1	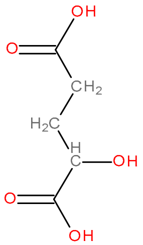	OC(CCC(O)=O)C(O)=O	148.114	$C_5H_8O_5$	2-hydroxypentanedioic acid	InChI=1S/C5H8O5/c6-3(5(9)10)1-2-4(7)8/h3,6H,1-2H2,(H,7,8)(H,9,10)	HWXBTNAVRSUOJR-UHFFFAOYSA-N
28	3Aa1	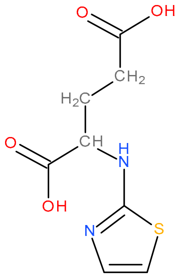	OC(=O)CCC(NC1=NC=CS1)C(O)=O	230.24	$C_8H_{10}N_2O_4S$	2-[(1,3-thiazol-2-yl)amino]pentanedioic acid	InChI=1S/C8H10N2O4S/c11-6(12)2-1-5(7(13)14)10-8-9-3-4-15-8/h3-5H,1-2H2,(H,9,10)(H,11,12)(H,13,14)	COUCFTITYQVLOF-UHFFFAOYSA-N

No.	ID code	Structural formula	SMILES	Molecular weight	Molecular formula	IUPAC name	InChI String	InChI Key
29	3Aa2	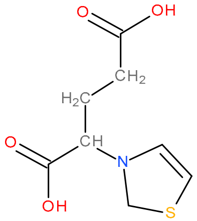	OC(=O)CCC(N1CSC=C1)C(O)=O	217.24	$C_8H_{11}NO_4S$	2-(2,3-dihydro-1,3-thiazol-3-yl)pentanedioic acid	InChI=1S/C8H11NO4S/c10-7(11)2-1-6(8(12)13)9-3-4-14-5-9/h3-4,6H,1-2,5H2,(H,10,11)(H,12,13)	FTQFPUBQARGJBF-UHFFFAOYSA-N
30	3Aa3	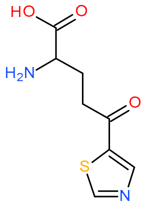	NC(CCC(=O)C1=CN=CS1)C(O)=O	214.24	$C_8H_{10}N_2O_3S$	2-amino-5-oxo-5-(1,3-thiazol-5-yl)pentanoic acid	InChI=1S/C8H10N2O3S/c9-5(8(12)13)1-2-6(11)7-3-10-4-14-7/h3-5H,1-2,9H2,(H,12,13)	PTRRYBHQTDDNOS-UHFFFAOYSA-N
31	3Aa4	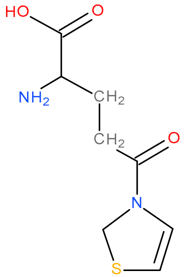	NC(CCC(=O)N1CSC=C1)C(O)=O	216.26	$C_8H_{12}N_2O_3S$	2-amino-5-(2,3-dihydro-1,3-thiazol-3-yl)-5-oxopentanoic acid	InChI=1S/C8H12N2O3S/c9-6(8(12)13)1-2-7(11)10-3-4-14-5-10/h3-4,6H,1-2,5,9H2,(H,12,13)	LLBOCRFUIYTIBD-UHFFFAOYSA-N
32	3Aa5	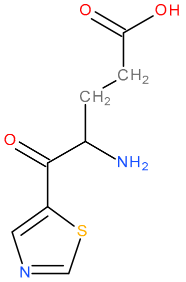	NC(CCC(O)=O)C(=O)C1=CN=CS1	214.24	$C_8H_{10}N_2O_3S$	4-amino-5-oxo-5-(1,3-thiazol-5-yl)pentanoic acid	InChI=1S/C8H10N2O3S/c9-5(1-2-7(11)12)8(13)6-3-10-4-14-6/h3-5H,1-2,9H2,(H,11,12)	WVQHOIOTBADZQN-UHFFFAOYSA-N

No.	ID code	Structural formula	SMILES	Molecular weight	Molecular formula	IUPAC name	InChI String	InChI Key
33	3Aa6	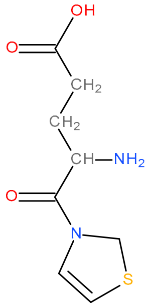	NC(CCC(O)=O)C(=O)N1CSC=C1	216.26	$C_8H_{12}N_2O_3S$	4-amino-5-(2,3-dihydro-1,3-thiazol-3-yl)-5-oxopentanoic acid	InChI=1S/C8H12N2O3S/c9-6(1-2-7(11)12)8(13)10-3-4-14-5-10/h3-4,6H,1-2,5,9H2,(H,11,12)	DREMUKVMUYKPSG-UHFFFAOYSA-N
34	3Aa7	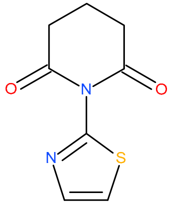	O=C1CCCC(=O)N1C1=NC=CS1	196.22	$C_8H_8N_2O_2S$	1-(1,3-thiazol-2-yl)piperidine-2,6-dione	InChI=1S/C8H8N2O2S/c11-6-2-1-3-7(12)10(6)8-9-4-5-13-8/h4-5H,1-3H2	KLUUFWQUMITGBV-UHFFFAOYSA-N
35	3Aa8	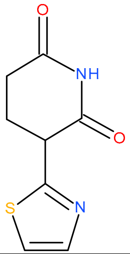	O=C1CCC(C2=NC=CS2)C(=O)N1	196.22	$C_8H_8N_2O_2S$	3-(1,3-thiazol-2-yl)piperidine-2,6-dione	InChI=1S/C8H8N2O2S/c11-6-2-1-5(7(12)10-6)8-9-3-4-13-8/h3-5H,1-2H2,(H,10,11,12)	YXZQPNDUEFTSFL-UHFFFAOYSA-N
36	3Aa9	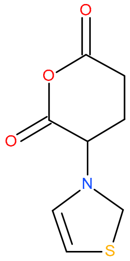	O=C1CCC(N2CSC=C2)C(=O)O1	199.22	$C_8H_9NO_3S$	3-(2,3-dihydro-1,3-thiazol-3-yl)oxane-2,6-dione	InChI=1S/C8H9NO3S/c10-7-2-1-6(8(11)12-7)9-3-4-13-5-9/h3-4,6H,1-2,5H2	QZCBBFBGLCBESD-UHFFFAOYSA-N

No.	ID code	Structural formula	SMILES	Molecular weight	Molecular formula	IUPAC name	InChI String	InChI Key
37	3Ab10	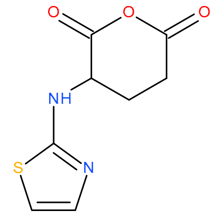	O=C1CCC(NC2=NC=CS2)C(=O)O1	212.22	$C_8H_8N_2O_3S$	3-[(1,3-thiazol-2-yl)amino]oxane-2,6-dione	InChI=1S/C8H8N2O3S/c11-6-2-1-5(7(12)13-6)10-8-9-3-4-14-8/h3-5H,1-2H2,(H,9,10)	LYMKTCGBZJZMJT-UHFFFAOYSA-N
38	3Ba1	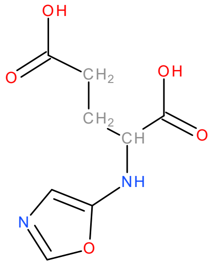	OC(=O)CCC(NC1=CN=CO1)C(O)=O	214.177	$C_8H_{10}N_2O_5$	2-[(1,3-oxazol-5-yl)amino]pentanedioic acid	InChI=1S/C8H10N2O5/c11-7(12)2-1-5(8(13)14)10-6-3-9-4-15-6/h3-5,10H,1-2H2,(H,11,12)(H,13,14)	VYBRWYKIDGAHKK-UHFFFAOYSA-N
39	3Ba2	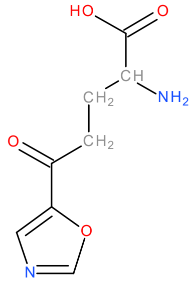	NC(CCC(=O)C1=CN=CO1)C(O)=O	198.178	$C_8H_{10}N_2O_4$	2-amino-5-(1,3-oxazol-5-yl)-5-oxopentanoic acid	InChI=1S/C8H10N2O4/c9-5(8(12)13)1-2-6(11)7-3-10-4-14-7/h3-5H,1-2,9H2,(H,12,13)	QPERUFXSMXFFRT-UHFFFAOYSA-N
40	3Ba3	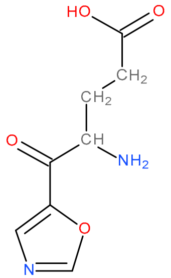	NC(CCC(O)=O)C(=O)C1=CN=CO1	198.178	$C_8H_{10}N_2O_4$	4-amino-5-(1,3-oxazol-5-yl)-5-oxopentanoic acid	InChI=1S/C8H10N2O4/c9-5(1-2-7(11)12)8(13)6-3-10-4-14-6/h3-5H,1-2,9H2,(H,11,12)	PQAQFMNDADADSD-UHFFFAOYSA-N

No.	ID code	Structural formula	SMILES	Molecular weight	Molecular formula	IUPAC name	InChI String	InChI Key
41	3Ba4	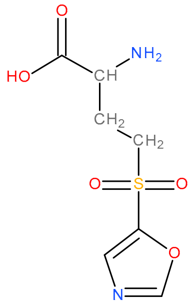	NC(CCS(=O)(=O)C1=CN=CO1)C(O)=O	234.23	$C_7H_{10}N_2O_5S$	2-amino-4-(1,3-oxazole-5-sulfonyl)butanoic acid	InChI=1S/C7H10N2O5S/c8-5(7(10)11)1-2-15(12,13)6-3-9-4-14-6/h3-5H,1-2,8H2,(H,10,11)	QGLSYLKIZWMURH-UHFFFAOYSA-N
42	3Ba5	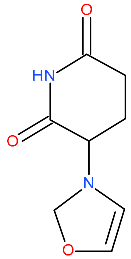	O=C1CCC(N2COC=C2)C(=O)N1	182.179	$C_8H_{10}N_2O_3$	3-(2,3-dihydro-1,3-oxazol-3-yl)piperidine-2,6-dione	InChI=1S/C8H10N2O3/c11-7-2-1-6(8(12)9-7)10-3-4-13-5-10/h3-4,6H,1-2,5H2,(H,9,11,12)	RMYQRMNPXUEMHX-UHFFFAOYSA-N
43	3Ba6	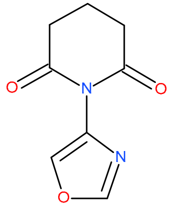	O=C1CCCC(=O)N1C1=COC=N1	180.163	$C_8H_8N_2O_3$	1-(1,3-oxazol-4-yl)piperidine-2,6-dione	InChI=1S/C8H8N2O3/c11-7-2-1-3-8(12)10(7)6-4-13-5-9-6/h4-5H,1-3H2	KRFQKBUQYJNZAK-UHFFFAOYSA-N
44	3Bb7	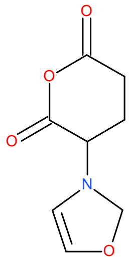	O=C1CCC(N2COC=C2)C(=O)O1	183.163	$C_8H_9NO_4$	3-(2,3-dihydro-1,3-oxazol-3-yl)oxane-2,6-dione	InChI=1S/C8H9NO4/c10-7-2-1-6(8(11)13-7)9-3-4-12-5-9/h3-4,6H,1-2,5H2	LEXIVLNRNLGLFK-UHFFFAOYSA-N

No.	ID code	Structural formula	SMILES	Molecular weight	Molecular formula	IUPAC name	InChI String	InChI Key
45	3Bb8	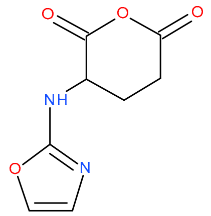	O=C1CCC(NC2=NC=CO2)C(=O)O1	196.162	$C_8H_8N_2O_4$	3-[(1,3-oxazol-2-yl)amino]oxane-2,6-dione	InChI=1S/C8H8N2O4/c11-6-2-1-5(7(12)14-6)10-8-9-3-4-13-8/h3-5H,1-2H2,(H,9,10)	NNJKXRQMIMZLLF-UHFFFAOYSA-N
46	4Aa1	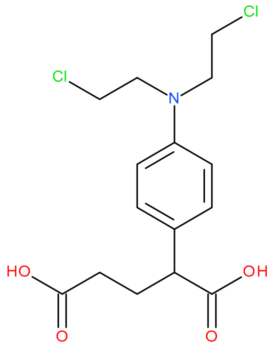	OC(=O)CCC(C(O)=O)C1=CC=C(C=C1)N(CCCl)CCCl	348.22	$C_{15}H_{19}Cl_2NO_4$	2-{4-[bis(2-chloroethyl)amino]phenyl}pentanedioic acid	InChI=1S/C15H19Cl2NO4/c16-7-9-18(10-8-17)12-3-1-11(2-4-12)13(15(21)22)5-6-14(19)20/h1-4,13H,5-10H2,(H,19,20)(H,21,22)	MOSFIJXAXDLOML-UHFFFAOYSA-N
47	4Aa2	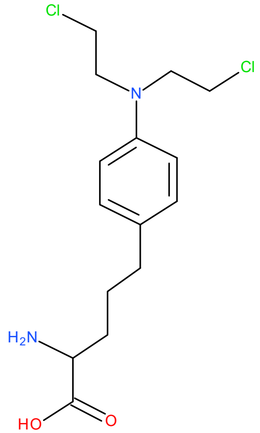	NC(CCCC1=CC=C(C=C1)N(CCCl)CCCl)C(O)=O	333.25	$C_{15}H_{22}Cl_2N_2O_2$	2-amino-5-{4-[bis(2-chloroethyl)amino]phenyl}pentanoic acid	InChI=1S/C15H22Cl2N2O2/c16-8-10-19(11-9-17)13-6-4-12(5-7-13)2-1-3-14(18)15(20)21/h4-7,14H,1-3,8-11,18H2,(H,20,21)	YSSSVODKUJTKAF-UHFFFAOYSA-N

No.	ID code	Structural formula	SMILES	Molecular weight	Molecular formula	IUPAC name	InChI String	InChI Key
48	4Aa3	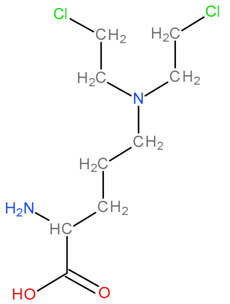	NC(CCCN(CCCl)CCCl)C(O)=O	257.16	$C_9H_{18}Cl_2N_2O_2$	2-amino-5-[bis(2-chloroethyl)amino]pentanoic acid	InChI=1S/C9H18Cl2N2O2/c10-3-6-13(7-4-11)5-1-2-8(12)9(14)15/h8H,1-7,12H2,(H,14,15)	IEDWQWUZMVJOSQ-UHFFFAOYSA-N
49	4Ab1	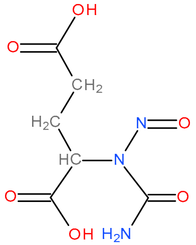	NC(=O)N(N=O)C(CCC(O)=O)C(O)=O	219.153	$C_6H_9N_3O_6$	2-[carbamoyl(nitroso)amino]pentanedioic acid	InChI=1S/C6H9N3O6/c7-6(14)9(8-15)3(5(12)13)1-2-4(10)11/h3H,1-2H2,(H2,7,14)(H,10,11)(H,12,13)	DZPDAZBHGBSWTO-UHFFFAOYSA-N
50	4Ab2	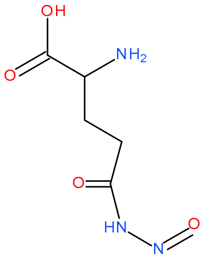	NC(CCC(=O)NN=O)C(O)=O	175.144	$C_5H_9N_3O_4$	2-amino-4-(N'-oxohydrazinecarbonyl)butanoic acid	InChI=1S/C5H9N3O4/c6-3(5(10)11)1-2-4(9)7-8-12/h3H,1-2,6H2,(H,10,11)(H,7,9,12)	CDUHCSDROKGGAX-UHFFFAOYSA-N
51	4Ac1	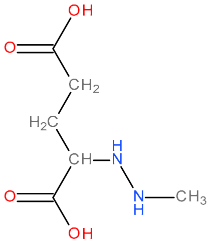	CNNC(CCC(O)=O)C(O)=O	176.172	$C_6H_{12}N_2O_4$	2-(2-methylhydrazin-1-yl)pentanedioic acid	InChI=1S/C6H12N2O4/c1-7-8-4(6(11)12)2-3-5(9)10/h4,7-8H,2-3H2,1H3,(H,9,10)(H,11,12)	HGWHOHGOLUXKQT-UHFFFAOYSA-N

No.	ID code	Structural formula	SMILES	Molecular weight	Molecular formula	IUPAC name	InChI String	InChI Key
52	4Ad1	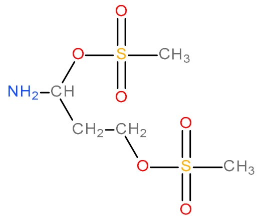	CS(=O)(=O)OCCC(N)OS(C)(=O)=O	247.28	$C_5H_{13}NO_6S_2$	1-amino-3-(methanesulfonyloxy)propyl methanesulfonate	InChI=1S/C5H13NO6S2/c1-13(7,8)11-4-3-5(6)12-14(2,9)10/h5H,3-4,6H2,1-2H3	NPHGXBQFTVQTFH-UHFFFAOYSA-N
53	4Ad2	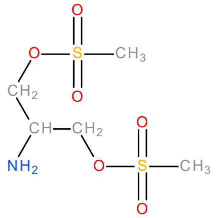	CS(=O)(=O)OCC(N)COS(C)(=O)=O	247.28	$C_5H_{13}NO_6S_2$	2-amino-3-(methanesulfonyloxy)propyl methanesulfonate	InChI=1S/C5H13NO6S2/c1-13(7,8)11-3-5(6)4-12-14(2,9)10/h5H,3-4,6H2,1-2H3	OJIHJLWBACHVEK-UHFFFAOYSA-N
54	4Ad3	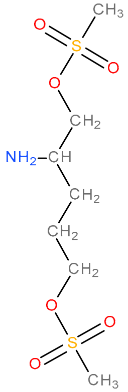	CS(=O)(=O)OCCCC(N)COS(C)(=O)=O	275.33	$C_7H_{17}NO_6S_2$	2-amino-5-(methanesulfonyloxy)pentyl methanesulfonate	InChI=1S/C7H17NO6S2/c1-15(9,10)13-5-3-4-7(8)6-14-16(2,11)12/h7H,3-6,8H2,1-2H3	LXWPOHSRFRSTMX-UHFFFAOYSA-N
55	4Ae1	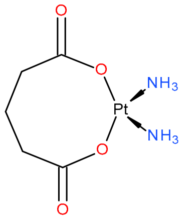	N.N.O=C1CCCC(=O)O[Pt]O1	359.245	$C_5H_{12}N_2O_4Pt$	2,2-diimino-1,3-dioxa-2-platinacyclooctane-4,8-dione	InChI=1S/C5H8O4.2H2N.Pt/c6-4(7)2-1-3-5(8)9;;;/h1-3H2,(H,6,7)(H,8,9);2*1H2;/q;2*-1;+4/p-2	LZNMYKZLJCAVHB-UHFFFAOYSA-L

No.	ID code	Structural formula	SMILES	Molecular weight	Molecular formula	IUPAC name	InChI String	InChI Key
56	4Ae2	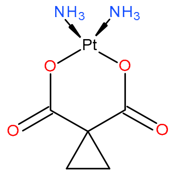	N.N.O=C1O[Pt]OC(=O)C11CC1	357.229	$C_5H_{10}N_2O_4Pt$	6,6-diimino-5,7-dioxa-6-platinaspiro[2.5]octane-4,8-dione	InChI=1S/C5H6O4.2H2N.Pt/c6-3(7)5(1-2-5)4(8)9;;;/h1-2H2,(H,6,7)(H,8,9);2*1H2;/q;2*-1;+4/p-2	BRHJVUWBZGXLHE-UHFFFAOYSA-L
57	4Ba1	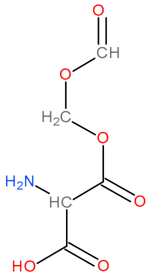	NC(C(O)=O)C(=O)OCOC=O	177.112	$C_5H_7NO_6$	2-amino-3-[(formyloxy)methoxy]-3-oxopropanoic acid	InChI=1S/C5H7NO6/c6-3(4(8)9)5(10)12-2-11-1-7/h1,3H,2,6H2,(H,8,9)	AJBXFUNGAPVXCW-UHFFFAOYSA-N
58	4Ba2	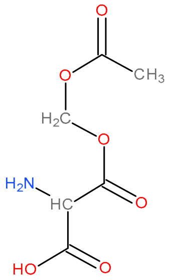	CC(=O)OCOC(=O)C(N)C(O)=O	191.139	$C_6H_9NO_6$	3-[(acetyloxy)methoxy]-2-amino-3-oxopropanoic acid	InChI=1S/C6H9NO6/c1-3(8)12-2-13-6(11)4(7)5(9)10/h4H,2,7H2,1H3,(H,9,10)	VMQNAFGZNCKKLS-UHFFFAOYSA-N
59	4Ba3	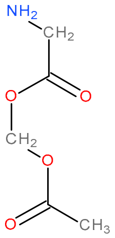	CC(=O)OCOC(=O)CN	147.13	$C_5H_9NO_4$	(acetyloxy)methyl 2-aminoacetate	InChI=1S/C5H9NO4/c1-4(7)9-3-10-5(8)2-6/h2-3,6H2,1H3	MGRXHFJZHMVAKB-UHFFFAOYSA-N

No.	ID code	Structural formula	SMILES	Molecular weight	Molecular formula	IUPAC name	InChI String	InChI Key
60	4Ba4	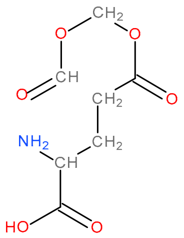	NC(CCC(=O)OCOC=O)C(O)=O	205.166	$C_7H_{11}NO_6$	2-amino-5-[(formyloxy)methoxy]-5-oxopentanoic acid	InChI=1S/C7H11NO6/c8-5(7(11)12)1-2-6(10)14-4-13-3-9/h3,5H,1-2,4,8H2,(H,11,12)	KFGASKCYGLPOQZ-UHFFFAOYSA-N
61	4Ba5	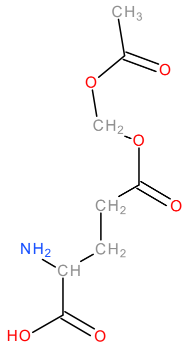	CC(=O)OCOC(=O)CCC(N)C(O)=O	219.193	$C_8H_{13}NO_6$	5-[(acetyloxy)methoxy]-2-amino-5-oxopentanoic acid	InChI=1S/C8H13NO6/c1-5(10)14-4-15-7(11)3-2-6(9)8(12)13/h6H,2-4,9H2,1H3,(H,12,13)	JSZGUAKGOBNWIO-UHFFFAOYSA-N
62	4Ba6	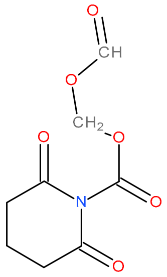	O=COCOC(=O)N1C(=O)CCCC1=O	215.161	$C_8H_9NO_6$	(formyloxy)methyl 2,6-dioxopiperidine-1-carboxylate	InChI=1S/C8H9NO6/c10-4-14-5-15-8(13)9-6(11)2-1-3-7(9)12/h4H,1-3,5H2	XKBWEHPUMQOETN-UHFFFAOYSA-N

No.	ID code	Structural formula	SMILES	Molecular weight	Molecular formula	IUPAC name	InChI String	InChI Key
63	4Ba7	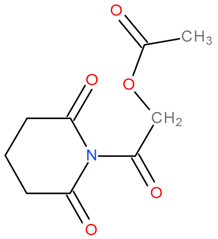	CC(=O)OCC(=O)N1C(=O)CCCC1=O	213.189	$C_9H_{11}NO_5$	2-(2,6-dioxopiperidin-1-yl)-2-oxoethyl acetate	InChI=1S/C9H11NO5/c1-6(11)15-5-9(14)10-7(12)3-2-4-8(10)13/h2-5H2,1H3	INUOZQWSZWUSJW-UHFFFAOYSA-N
64	4Ca1	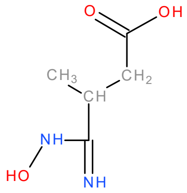	CC(CC(O)=O)C(=N)NO	146.146	$C_5H_{10}N_2O_3$	3-(N-hydroxycarbamimidoyl)-3-methylpropanoic acid	InChI=1S/C5H10N2O3/c1-3(2-4(8)9)5(6)7-10/h3,10H,2H2,1H3,(H2,6,7)(H,8,9)	OOQGEIDHPCFVDR-UHFFFAOYSA-N
65	4Ca2	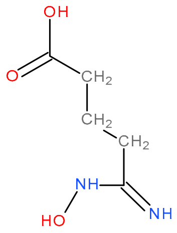	ONC(=N)CCCC(O)=O	146.146	$C_5H_{10}N_2O_3$	4-(N-hydroxycarbamimidoyl)butanoic acid	InChI=1S/C5H10N2O3/c6-4(7-10)2-1-3-5(8)9/h10H,1-3H2,(H2,6,7)(H,8,9)	QAJMVQKBIQNJFH-UHFFFAOYSA-N
66	4Ca3	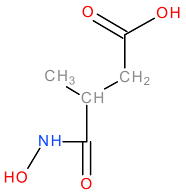	CC(CC(O)=O)C(=O)NO	147.13	$C_5H_9NO_4$	3-(hydroxycarbamoyl)-3-methylpropanoic acid	InChI=1S/C5H9NO4/c1-3(2-4(7)8)5(9)6-10/h3,10H,2H2,1H3,(H,6,9)(H,7,8)	MEFOEISBUCQCBX-UHFFFAOYSA-N

No.	ID code	Structural formula	SMILES	Molecular weight	Molecular formula	IUPAC name	InChI String	InChI Key
67	4Ca4	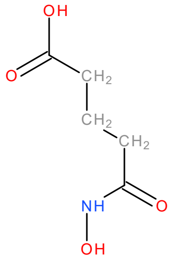	ONC(=O)CCCC(O)=O	147.13	$C_5H_9NO_4$	4-(hydroxycarbamoyl)butanoic acid	InChI=1S/C5H9NO4/c7-4(6-10)2-1-3-5(8)9/h10H,1-3H2,(H,6,7)(H,8,9)	WNMOIVFBJXRXSY-UHFFFAOYSA-N
68	4Cb1	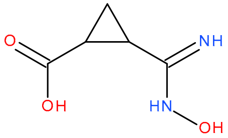	ONC(=N)C1CC1C(O)=O	144.13	$C_5H_8N_2O_3$	2-(N-hydroxycarbamimidoyl)cyclopropane-1-carboxylic acid	InChI=1S/C5H8N2O3/c6-4(7-10)2-1-3(2)5(8)9/h2-3,10H,1H2,(H2,6,7)(H,8,9)	XGIFGDFXFLPXID-UHFFFAOYSA-N
69	4Cb2	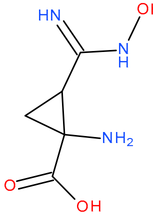	NC1(CC1C(=N)NO)C(O)=O	159.145	$C_5H_9N_3O_3$	1-amino-2-(N-hydroxycarbamimidoyl)cyclopropane-1-carboxylic acid	InChI=1S/C5H9N3O3/c6-3(8-11)2-1-5(2,7)4(9)10/h2,11H,1,7H2,(H2,6,8)(H,9,10)	SIXAHSQNNNGKON-UHFFFAOYSA-N
70	4Cb3	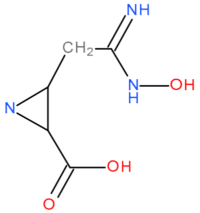	ONC(=N)CC1NC1C(O)=O	159.145	$C_5H_9N_3O_3$	3-[(N-hydroxycarbamimidoyl)methyl]aziridine-2-carboxylic acid	InChI=1S/C5H9N3O3/c6-3(8-11)1-2-4(7-2)5(9)10/h2,4,7,11H,1H2,(H2,6,8)(H,9,10)	DQJFFVUIGCSRIY-UHFFFAOYSA-N
71	4Cb4	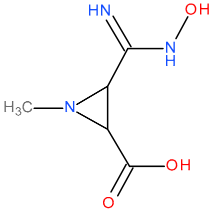	CN1C(C1C(=N)NO)C(O)=O	159.145	$C_5H_9N_3O_3$	3-(N-hydroxycarbamimidoyl)-1-methylaziridine-2-carboxylic acid	InChI=1S/C5H9N3O3/c1-8-2(4(6)7-11)3(8)5(9)10/h2-3,11H,1H3,(H2,6,7)(H,9,10)	GMVNMHGBMYOXJZ-UHFFFAOYSA-N

No.	ID code	Structural formula	SMILES	Molecular weight	Molecular formula	IUPAC name	InChI String	InChI Key
72	4Cb5	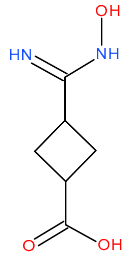	ONC(=N)C1CC(C1)C(O)=O	158.157	$C_6H_{10}N_2O_3$	3-(N-hydroxycarbamimidoyl)cyclobutane-1-carboxylic acid	InChI=1S/C6H10N2O3/c7-5(8-11)3-1-4(2-3)6(9)10/h3-4,11H,1-2H2,(H2,7,8)(H,9,10)	VIAKIMZYMCMRRE-UHFFFAOYSA-N
73	4Cb6	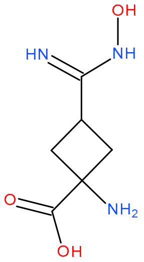	NC1(CC(C1)C(=N)NO)C(O)=O	173.172	$C_6H_{11}N_3O_3$	1-amino-3-(N-hydroxycarbamimidoyl)cyclobutane-1-carboxylic acid	InChI=1S/C6H11N3O3/c7-4(9-12)3-1-6(8,2-3)5(10)11/h3,12H,1-2,8H2,(H2,7,9)(H,10,11)	SRKMBICPXQFNTE-UHFFFAOYSA-N
74	4Cb7	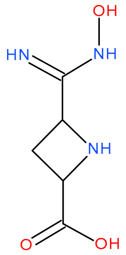	ONC(=N)C1CC(N1)C(O)=O	159.145	$C_5H_9N_3O_3$	4-(N-hydroxycarbamimidoyl)azetidine-2-carboxylic acid	InChI=1S/C5H9N3O3/c6-4(8-11)2-1-3(7-2)5(9)10/h2-3,7,11H,1H2,(H2,6,8)(H,9,10)	CRSVVFUTSFLRBT-UHFFFAOYSA-N
75	4Da1	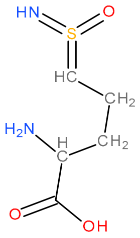	NC(CCC=S(=N)=O)C(O)=O	178.21	$C_5H_{10}N_2O_3S$	2-amino-5-[imino(oxo)-lambda6-sulfanylidene]pentanoic acid	InChI=1S/C5H10N2O3S/c6-4(5(8)9)2-1-3-11(7)10/h3-4,7H,1-2,6H2,(H,8,9)	XBPIIDUKNLDDHE-UHFFFAOYSA-N

No.	ID code	Structural formula	SMILES	Molecular weight	Molecular formula	IUPAC name	InChI String	InChI Key
76	4Da2	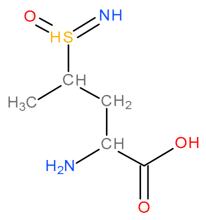	CC(CC(N)C(O)=O)S(=N)=O	180.22	$C_5H_{12}N_2O_3S$	2-amino-4-[imino(oxo)-lambda6-sulfanyl]pentanoic acid	InChI=1S/C5H12N2O3S/c1-3(11(7)10)2-4(6)5(8)9/h3-4,7,11H,2,6H2,1H3,(H,8,9)	QOOMKLVWZFMOLV-UHFFFAOYSA-N
77	4Da3	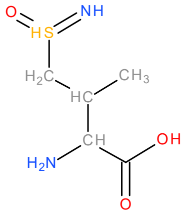	CC(CS(=N)=O)C(N)C(O)=O	180.22	$C_5H_{12}N_2O_3S$	2-amino-4-[imino(oxo)-lambda6-sulfanyl]-3-methylbutanoic acid	InChI=1S/C5H12N2O3S/c1-3(2-11(7)10)4(6)5(8)9/h3-4,7,11H,2,6H2,1H3,(H,8,9)	FDQFGEXLUNEELH-UHFFFAOYSA-N
78	4Da4	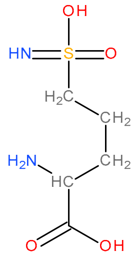	NC(CCCS(O)(=N)=O)C(O)=O	196.22	$C_5H_{12}N_2O_4S$	2-amino-5-[hydroxy(imino)oxo-lambda6-sulfanyl]pentanoic acid	InChI=1S/C5H12N2O4S/c6-4(5(8)9)2-1-3-12(7,10)11/h4H,1-3,6H2,(H,8,9)(H2,7,10,11)	PRADIGYGFWSNIK-UHFFFAOYSA-N
79	4Da5	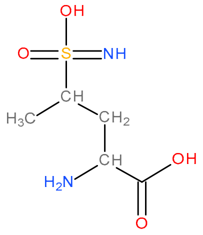	CC(CC(N)C(O)=O)S(O)(=N)=O	196.22	$C_5H_{12}N_2O_4S$	2-amino-4-[hydroxy(imino)oxo-lambda6-sulfanyl]pentanoic acid	InChI=1S/C5H12N2O4S/c1-3(12(7,10)11)2-4(6)5(8)9/h3-4H,2,6H2,1H3,(H,8,9)(H2,7,10,11)	OYPMNZNTRUDKOL-UHFFFAOYSA-N

No.	ID code	Structural formula	SMILES	Molecular weight	Molecular formula	IUPAC name	InChI String	InChI Key
80	4Da6	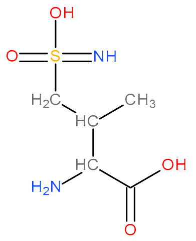	CC(CS(O)(=N)=O)C(N)C(O)=O	196.22	$C_5H_{12}N_2O_4S$	2-amino-4-[hydroxy(imino)oxo-lambda6-sulfanyl]-3-methylbutanoic acid	InChI=1S/C5H12N2O4S/c1-3(2-12(7,10)11)4(6)5(8)9/h3-4H,2,6H2,1H3,(H,8,9)(H2,7,10,11)	HPOATHZDVDMTSL-UHFFFAOYSA-N
81	4Da7	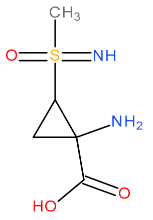	CS(=N)(=O)C1CC1(N)C(O)=O	178.21	$C_5H_{10}N_2O_3S$	1-amino-2-[imino(methyl)oxo-lambda6-sulfanyl]cyclopropane-1-carboxylic acid	InChI=1S/C5H10N2O3S/c1-11(7,10)3-2-5(3,6)4(8)9/h3,7H,2,6H2,1H3,(H,8,9)	YJGMPCSCQDUFMV-UHFFFAOYSA-N
82	4Da8	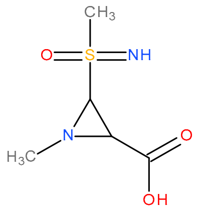	CN1C(C1S(C)(=N)=O)C(O)=O	178.21	$C_5H_{10}N_2O_3S$	3-[imino(methyl)oxo-lambda6-sulfanyl]-1-methylaziridine-2-carboxylic acid	InChI=1S/C5H10N2O3S/c1-7-3(5(8)9)4(7)11(2,6)10/h3-4,6H,1-2H3,(H,8,9)	RINRQUVMNZBFRE-UHFFFAOYSA-N
83	4Da9	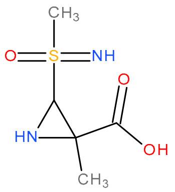	CC1(NC1S(C)(=N)=O)C(O)=O	178.21	$C_5H_{10}N_2O_3S$	3-[imino(methyl)oxo-lambda6-sulfanyl]-2-methylaziridine-2-carboxylic acid	InChI=1S/C5H10N2O3S/c1-5(4(8)9)3(7-5)11(2,6)10/h3,6-7H,1-2H3,(H,8,9)	JWUGTMIVGSCWEJ-UHFFFAOYSA-N

No.	ID code	Structural formula	SMILES	Molecular weight	Molecular formula	IUPAC name	InChI String	InChI Key
84	4Da10	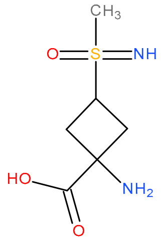	CS(=N)(=O)C1CC(N)(C1)C(O)=O	192.23	$C_6H_{12}N_2O_3S$	1-amino-3-[imino(methyl)oxo-lambda6-sulfanyl]cyclobutane-1-carboxylic acid	InChI=1S/C6H12N2O3S/c1-12(8,11)4-2-6(7,3-4)5(9)10/h4,8H,2-3,7H2,1H3,(H,9,10)	SYTQOEQFJJBMFZ-UHFFFAOYSA-N
85	4Da11	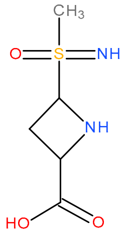	CS(=N)(=O)C1CC(N1)C(O)=O	178.21	$C_5H_{10}N_2O_3S$	4-[imino(methyl)oxo-lambda6-sulfanyl]azetidine-2-carboxylic acid	InChI=1S/C5H10N2O3S/c1-11(6,10)4-2-3(7-4)5(8)9/h3-4,6-7H,2H2,1H3,(H,8,9)	RFJWDYGLNRPCQW-UHFFFAOYSA-N
86	4Db1	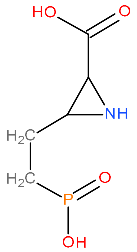	OC(=O)C1NC1CCP(O)=O	179.112	$C_5H_{10}NO_4P$	3-[2-(hydroxyphosphonoyl)ethyl]aziridine-2-carboxylic acid	InChI=1S/C5H10NO4P/c7-5(8)4-3(6-4)1-2-11(9)10/h3-4,6,11H,1-2H2,(H,7,8)(H,9,10)	ROIDKHCLEYTORA-UHFFFAOYSA-N
87	4Db2	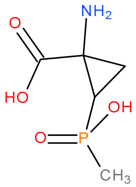	CP(O)(=O)C1CC1(N)C(O)=O	179.112	$C_5H_{10}NO_4P$	1-amino-2-[hydroxy(methyl)phosphoryl]cyclopropane-1-carboxylic acid	InChI=1S/C5H10NO4P/c1-11(9,10)3-2-5(3,6)4(7)8/h3H,2,6H2,1H3,(H,7,8)(H,9,10)	BVORIWUDXJXNKQ-UHFFFAOYSA-N

No.	ID code	Structural formula	SMILES	Molecular weight	Molecular formula	IUPAC name	InChI String	InChI Key
88	4Db3	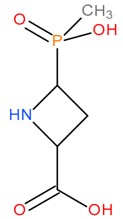	CP(O)(=O)C1CC(N1)C(O)=O	179.112	$C_5H_{10}NO_4P$	4-[hydroxy(methyl)phosphoryl]azetidine-2-carboxylic acid	InChI=1S/C5H10NO4P/c1-11(9,10)4-2-3(6-4)5(7)8/h3-4,6H,2H2,1H3,(H,7,8)(H,9,10)	IHPLWMFUNRLHTF-UHFFFAOYSA-N
89	4Db4	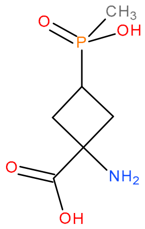	CP(O)(=O)C1CC(N)(C1)C(O)=O	193.139	$C_6H_{12}NO_4P$	1-amino-3-[hydroxy(methyl)phosphoryl]cyclobutane-1-carboxylic acid	InChI=1S/C6H12NO4P/c1-12(10,11)4-2-6(7,3-4)5(8)9/h4H,2-3,7H2,1H3,(H,8,9)(H,10,11)	YFDAPDBYAJGKPM-UHFFFAOYSA-N
90	4Db5	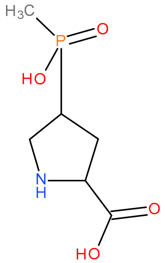	CP(O)(=O)C1CNC(C1)C(O)=O	193.139	$C_6H_{12}NO_4P$	4-[hydroxy(methyl)phosphoryl]pyrrolidine-2-carboxylic acid	InChI=1S/C6H12NO4P/c1-12(10,11)4-2-5(6(8)9)7-3-4/h4-5,7H,2-3H2,1H3,(H,8,9)(H,10,11)	KINRKWHPXWCNQE-UHFFFAOYSA-N
91	4Db6	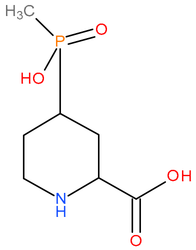	CP(O)(=O)C1CCNC(C1)C(O)=O	207.166	$C_7H_{14}NO_4P$	4-[hydroxy(methyl)phosphoryl]piperidine-2-carboxylic acid	InChI=1S/C7H14NO4P/c1-13(11,12)5-2-3-8-6(4-5)7(9)10/h5-6,8H,2-4H2,1H3,(H,9,10)(H,11,12)	WESMCDQHSFEJMX-UHFFFAOYSA-N

No.	ID code	Structural formula	SMILES	Molecular weight	Molecular formula	IUPAC name	InChI String	InChI Key
92	4Dc1	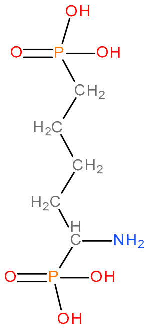	NC(CCCCP(O)(O)=O)P(O)(O)=O	247.124	$C_5H_{15}NO_6P_2$	(1-amino-5-phosphonopentyl)phosphonic acid	InChI=1S/C5H15NO6P2/c6-5(14(10,11)12)3-1-2-4-13(7,8)9/h5H,1-4,6H2,(H2,7,8,9)(H2,10,11,12)	CUIROQLTUJTNCG-UHFFFAOYSA-N
93	4Dc2	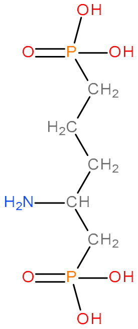	NC(CCCP(O)(O)=O)CP(O)(O)=O	247.124	$C_5H_{15}NO_6P_2$	(2-amino-5-phosphonopentyl)phosphonic acid	InChI=1S/C5H15NO6P2/c6-5(4-14(10,11)12)2-1-3-13(7,8)9/h5H,1-4,6H2,(H2,7,8,9)(H2,10,11,12)	XMSAHCRTGNRVOH-UHFFFAOYSA-N
94	4Dc3	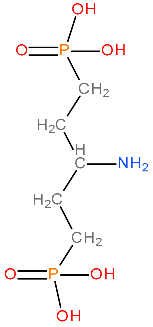	NC(CCP(O)(O)=O)CCP(O)(O)=O	247.124	$C_5H_{15}NO_6P_2$	(3-amino-5-phosphonopentyl)phosphonic acid	InChI=1S/C5H15NO6P2/c6-5(1-3-13(7,8)9)2-4-14(10,11)12/h5H,1-4,6H2,(H2,7,8,9)(H2,10,11,12)	BJHPZCJWPNIHBQ-UHFFFAOYSA-N

No.	ID code	Structural formula	SMILES	Molecular weight	Molecular formula	IUPAC name	InChI String	InChI Key
95	4Dc4	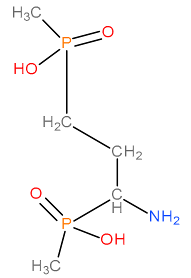	CP(O)(=O)CCC(N)P(C)(O)=O	215.126	$C_5H_{15}NO_4P_2$	{1-amino-3-[hydroxy(methyl)phosphoryl]propyl}(methyl)phosphinic acid	InChI=1S/C5H15NO4P2/c1-11(7,8)4-3-5(6)12(2,9)10/h5H,3-4,6H2,1-2H3,(H,7,8)(H,9,10)	YKAMTHKVSPQIQX-UHFFFAOYSA-N
96	4Dc5	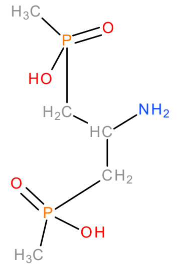	CP(O)(=O)CC(N)CP(C)(O)=O	215.126	$C_5H_{15}NO_4P_2$	{2-amino-3-[hydroxy(methyl)phosphoryl]propyl}(methyl)phosphinic acid	InChI=1S/C5H15NO4P2/c1-11(7,8)3-5(6)4-12(2,9)10/h5H,3-4,6H2,1-2H3,(H,7,8)(H,9,10)	RNWTUXIXSQISBS-UHFFFAOYSA-N
97	4Dc6	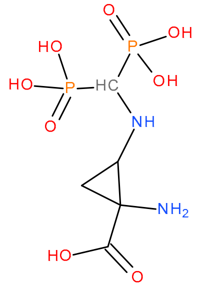	NC1(CC1NC(P(O)(O)=O)P(O)(O)=O)C(O)=O	290.105	$C_5H_{12}N_2O_8P_2$	1-amino-2-[(diphosphonomethyl)amino]cyclopropane-1-carboxylic acid	InChI=1S/C5H12N2O8P2/c6-5(3(8)9)1-2(5)7-4(16(10,11)12)17(13,14)15/h2,4,7H,1,6H2,(H,8,9)(H2,10,11,12)(H2,13,14,15)	QWCNXXAJYFJSFF-UHFFFAOYSA-N

No.	ID code	Structural formula	SMILES	Molecular weight	Molecular formula	IUPAC name	InChI String	InChI Key
98	4Dc7	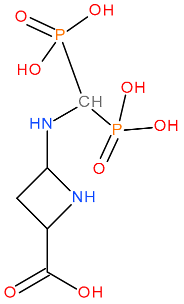	OC(=O)C1CC(NC(P(O)(O)=O)P(O)(O)=O)N1	290.105	$C_5H_{12}N_2O_8P_2$	4-[(diphosphonomethyl)amino]azetidine-2-carboxylic acid	InChI=1S/C5H12N2O8P2/c8-4(9)2-1-3(6-2)7-5(16(10,11)12)17(13,14)15/h2-3,5-7H,1H2,(H,8,9)(H2,10,11,12)(H2,13,14,15)	XGTGQLRSZGCOKU-UHFFFAOYSA-N
99	4Dc8	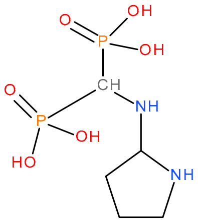	OP(O)(=O)C(NC1CCCN1)P(O)(O)=O	260.123	$C_5H_{14}N_2O_6P_2$	{phosphono[(pyrrolidin-2-yl)amino]methyl}phosphonic acid	InChI=1S/C5H14N2O6P2/c8-14(9,10)5(15(11,12)13)7-4-2-1-3-6-4/h4-7H,1-3H2,(H2,8,9,10)(H2,11,12,13)	ZGEZSLDXXJKKQC-UHFFFAOYSA-N
100	4Dd1.1	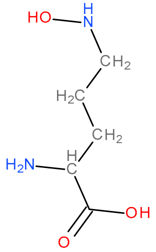	NC(CCCNO)C(O)=O	148.162	$C_5H_{12}N_2O_3$	2-amino-5-(hydroxyamino)pentanoic acid	InChI=1S/C5H12N2O3/c6-4(5(8)9)2-1-3-7-10/h4,7,10H,1-3,6H2,(H,8,9)	OZMJDTPATROLQC-UHFFFAOYSA-N
101	4Dd1.2	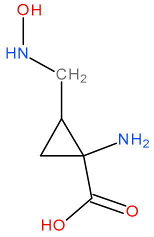	NC1(CC1CNO)C(O)=O	146.146	$C_5H_{10}N_2O_3$	1-amino-2-[(hydroxyamino)methyl]cyclopropane-1-carboxylic acid	InChI=1S/C5H10N2O3/c6-5(4(8)9)1-3(5)2-7-10/h3,7,10H,1-2,6H2,(H,8,9)	GIOGRIZQWPZFMM-UHFFFAOYSA-N

No.	ID code	Structural formula	SMILES	Molecular weight	Molecular formula	IUPAC name	InChI String	InChI Key
102	4Dd1 .3	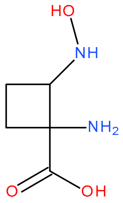	NC1(CCC1NO)C(O)=O	146.146	$C_5H_{10}N_2O_3$	1-amino-2-(hydroxyamino)cyclobutane-1-carboxylic acid	InChI=1S/C5H10N2O3/c6-5(4(8)9)2-1-3(5)7-10/h3,7,10H,1-2,6H2,(H,8,9)	FFPOXTKEZNXVCT-UHFFFAOYSA-N
103	4Dd1 .4	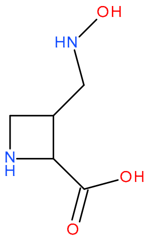	ONCC1CNC1C(O)=O	146.146	$C_5H_{10}N_2O_3$	3-[(hydroxyamino)methyl]azetidine-2-carboxylic acid	InChI=1S/C5H10N2O3/c8-5(9)4-3(1-6-4)2-7-10/h3-4,6-7,10H,1-2H2,(H,8,9)	LNGNICJYHSCSNX-UHFFFAOYSA-N
104	4Dd2 .1	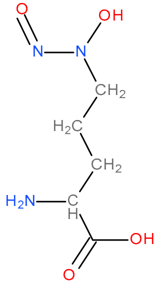	NC(CCCN(O)N=O)C(O)=O	177.16	$C_5H_{11}N_3O_4$	2-amino-5-[hydroxy(nitroso)amino]pentanoic acid	InChI=1S/C5H11N3O4/c6-4(5(9)10)2-1-3-8(12)7-11/h4,12H,1-3,6H2,(H,9,10)	YEKMBVYYHZMGJV-UHFFFAOYSA-N
105	4Dd3 .1	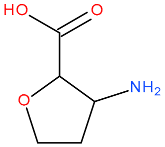	NC1CCOC1C(O)=O	131.131	$C_5H_9NO_3$	3-aminooxolane-2-carboxylic acid	InChI=1S/C5H9NO3/c6-3-1-2-9-4(3)5(7)8/h3-4H,1-2,6H2,(H,7,8)	OVGHASDQKHPORW-UHFFFAOYSA-N

No.	ID code	Structural formula	SMILES	Molecular weight	Molecular formula	IUPAC name	InChI String	InChI Key
106	4Dd4 .1	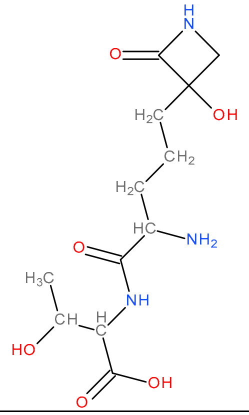	CC(O)C(NC(=O)C(N)CCCC1(O)CNC1=O)C(O)=O	303.315	$C_{12}H_{21}N_3O_6$	2-[2-amino-5-(3-hydroxy-2-oxoazetidin-3-yl)pentanamido]-3-hydroxybutanoic acid	InChI=1S/C12H21N3O6/c1-6(16)8(10(18)19)15-9(17)7(13)3-2-4-12(21)5-14-11(12)20/h6-8,16,21H,2-5,13H2,1H3,(H,14,20)(H,15,17)(H,18,19)	VJZZMLXYRMWFOO-UHFFFAOYSA-N
107	4Dd4 .2	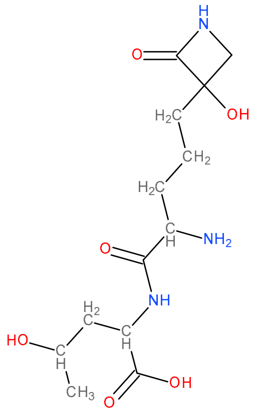	CC(O)CC(NC(=O)C(N)CCCC1(O)CNC1=O)C(O)=O	317.342	$C_{13}H_{23}N_3O_6$	2-[2-amino-5-(3-hydroxy-2-oxoazetidin-3-yl)pentanamido]-4-hydroxypentanoic acid	InChI=1S/C13H23N3O6/c1-7(17)5-9(11(19)20)16-10(18)8(14)3-2-4-13(22)6-15-12(13)21/h7-9,17,22H,2-6,14H2,1H3,(H,15,21)(H,16,18)(H,19,20)	YFYPDXFFNZRMTQ-UHFFFAOYSA-N

No.	ID code	Structural formula	SMILES	Molecular weight	Molecular formula	IUPAC name	InChI String	InChI Key
108	4Dd4.3	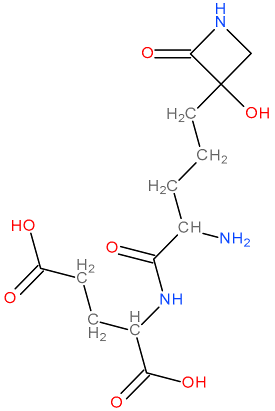	NC(CCCC1(O)CNC1=O)C(=O)NC(CCC(O)=O)C(O)=O	331.325	$C_{13}H_{21}N_3O_7$	2-[2-amino-5-(3-hydroxy-2-oxoazetidin-3-yl)pentanamido]pentanedioic acid	InChI=1S/C13H21N3O7/c14-7(2-1-5-13(23)6-15-12(13)22)10(19)16-8(11(20)21)3-4-9(17)18/h7-8,23H,1-6,14H2,(H,15,22)(H,16,19)(H,17,18)(H,20,21)	ATUQAECWGSNLCN-UHFFFAOYSA-N
109	4Dd4.m	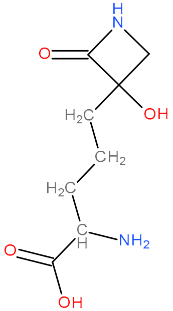	NC(CCCC1(O)CNC1=O)C(O)=O	202.21	$C_8H_{14}N_2O_4$	2-amino-5-(3-hydroxy-2-oxoazetidin-3-yl)pentanoic acid	InChI=1S/C8H14N2O4/c9-5(6(11)12)2-1-3-8(14)4-10-7(8)13/h5,14H,1-4,9H2,(H,10,13)(H,11,12)	XAKGKLREWVVPDH-UHFFFAOYSA-N

No.	ID code	Structural formula	SMILES	Molecular weight	Molecular formula	IUPAC name	InChI String	InChI Key
110	5Aa1	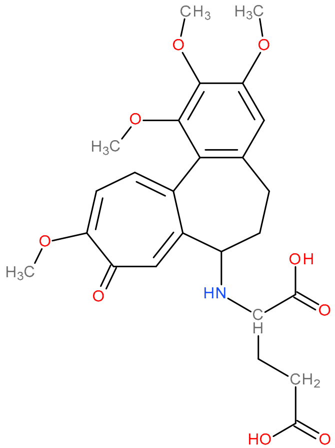	COC1=CC2=C(C(OC)=C1OC)C1=CC=C(OC)C(=O)C=C1C(CC2)NC(CCC(O)=O)C(O)=O	487.505	$C_{25}H_{29}NO_9$	2-({3,4,5,14-tetramethoxy-13-oxotricyclo[9,5,0,02,7]hexadeca-1(16),2(7),3,5,11,14-hexaen-10-yl}amino)pentanedioic acid	InChI=1S/C25H29NO9/c1-32-19-9-6-14-15(12-18(19)27)16(26-17(25(30)31)8-10-21(28)29)7-5-13-11-20(33-2)23(34-3)24(35-4)22(13)14/h6,9,11-12,16-17,26H,5,7-8,10H2,1-4H3,(H,28,29)(H,30,31)	WAMPULPOXAGNFE-UHFFFAOYSA-N
111	5Aa2	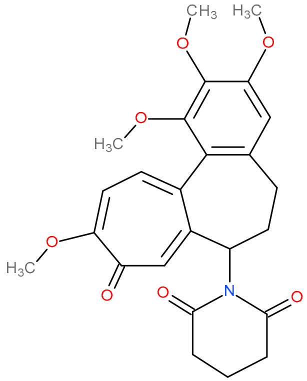	COC1=CC2=C(C(OC)=C1OC)C1=CC=C(OC)C(=O)C=C1C(CC2)N1C(=O)CCCC1=O	453.491	$C_{25}H_{27}NO_7$	1-{3,4,5,14-tetramethoxy-13-oxotricyclo[9,5,0,02,7]hexadeca-1(16),2(7),3,5,11,14-hexaen-10-yl}piperidine-2,6-dione	InChI=1S/C25H27NO7/c1-30-19-11-9-15-16(13-18(19)27)17(26-21(28)6-5-7-22(26)29)10-8-14-12-20(31-2)24(32-3)25(33-4)23(14)15/h9,11-13,17H,5-8,10H2,1-4H3	BUDQJDDSCSSRCD-UHFFFAOYSA-N

No.	ID code	Structural formula	SMILES	Molecular weight	Molecular formula	IUPAC name	InChI String	InChI Key
112	5Aa3	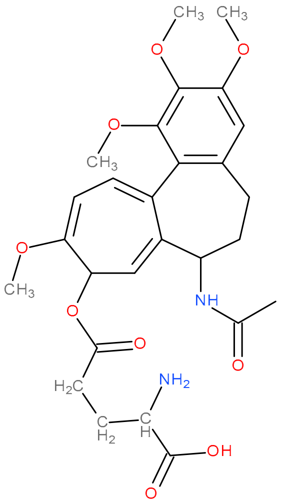	COC1=CC2=C(C(OC)=C1OC)C1=CC=C(OC)C(OC(=O)CCC(N)C(O)=O)C=C1C(CC2)NC(C)=O	530.574	$C_{27}H_{34}N_2O_9$	2-amino-5-({10-acetamido-3,4,5,14-tetramethoxytricyclo[9,5,0,02,7]hexadeca-1(16),2(7),3,5,11,14-hexaen-13-yl}oxy)-5-oxopentanoic acid	InChI=1S/C27H34N2O9/c1-14(30)29-19-9-6-15-12-22(35-3)25(36-4)26(37-5)24(15)16-7-10-20(34-2)21(13-17(16)19)38-23(31)11-8-18(28)27(32)33/h7,10,12-13,18-19,21H,6,8-9,11,28H2,1-5H3,(H,29,30)(H,32,33)	JQCAXMXOZZZSRB-UHFFFAOYSA-N
113	5Aa4	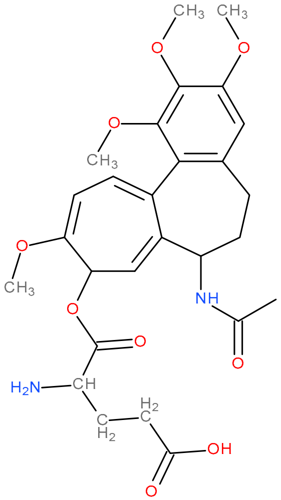	COC1=CC2=C(C(OC)=C1OC)C1=CC=C(OC)C(OC(=O)C(N)CCC(O)=O)C=C1C(CC2)NC(C)=O	530.574	$C_{27}H_{34}N_2O_9$	4-amino-5-({10-acetamido-3,4,5,14-tetramethoxytricyclo[9,5,0,02,7]hexadeca-1(16),2(7),3,5,11,14-hexaen-13-yl}oxy)-5-oxopentanoic acid	InChI=1S/C27H34N2O9/c1-14(30)29-19-9-6-15-12-22(35-3)25(36-4)26(37-5)24(15)16-7-10-20(34-2)21(13-17(16)19)38-27(33)18(28)8-11-23(31)32/h7,10,12-13,18-19,21H,6,8-9,11,28H2,1-5H3,(H,29,30)(H,31,32)	KGMTWHJUHSZYKQ-UHFFFAOYSA-N

No.	ID code	Structural formula	SMILES	Molecular weight	Molecular formula	IUPAC name	InChI String	InChI Key
114	5Ba1	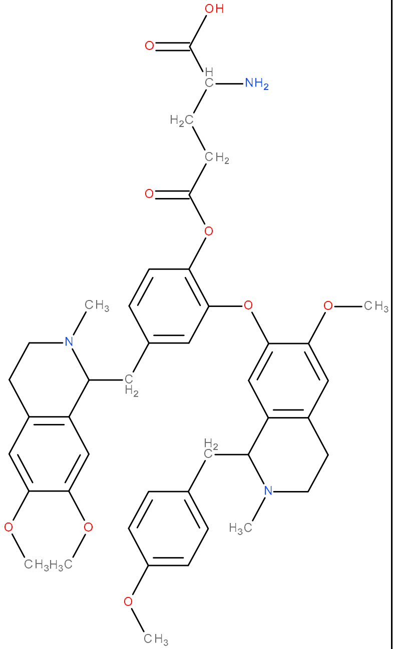	COC1=CC=C(CC2N(C)CCC3=C2C=C(OC2=C(OC(=O)CCC(N)C(O)=O)C=CC(CC4N(C)CCC5=C4C=C(OC)C(OC)=C5)=C2)C(OC)=C3)C=C1	753.893	$C_{43}H_{51}N_3O_9$	2-amino-5-{4-[(6,7-dimethoxy-2-methyl-1,2,3,4-tetrahydroisoquinolin-1-yl)methyl]-2-({6-methoxy-1-[(4-methoxyphenyl)methyl]-2-methyl-1,2,3,4-tetrahydroisoquinolin-7-yl}oxy)phenoxy}-5-oxopentanoic acid	InChI=1S/C43H51N3O9/c1-45-17-16-29-23-38(52-5)41(25-32(29)34(45)19-26-7-10-30(50-3)11-8-26)54-40-21-27(9-13-36(40)55-42(47)14-12-33(44)43(48)49)20-35-31-24-39(53-6)37(51-4)22-28(31)15-18-46(35)2/h7-11,13,21-25,33-35H,12,14-20,44H2,1-6H3,(H,48,49)	YAJQPCYSVBBDNV-UHFFFAOYSA-N

No.	ID code	Structural formula	SMILES	Molecular weight	Molecular formula	IUPAC name	InChI String	InChI Key
115	5Ba2	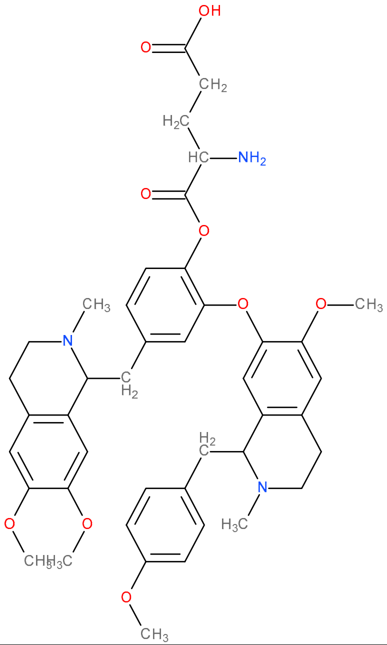	COC1=CC=C(CC2N(C)CCC3=C2C=C(OC2=C(OC(=O)C(N)CCC(O)=O)C=CC(CC4N(C)CCC5=C4C=C(OC)C(OC)=C5)=C2)C(OC)=C3)C=C1	753.893	$C_{43}H_{51}N_3O_9$	4-amino-5-{4-[(6,7-dimethoxy-2-methyl-1,2,3,4-tetrahydroisoquinolin-1-yl)methyl]-2-({6-methoxy-1-[(4-methoxyphenyl)methyl]-2-methyl-1,2,3,4-tetrahydroisoquinolin-7-yl}oxy)phenoxy}-5-oxopentanoic acid	InChI=1S/C43H51N3O9/c1-45-17-16-29-23-38(52-5)41(25-32(29)34(45)19-26-7-10-30(50-3)11-8-26)54-40-21-27(9-13-36(40)55-43(49)33(44)12-14-42(47)48)20-35-31-24-39(53-6)37(51-4)22-28(31)15-18-46(35)2/h7-11,13,21-25,33-35H,12,14-20,44H2,1-6H3,(H,47,48)	RJYSGIDJTWWGOJ-UHFFFAOYSA-N
116	5Ca1	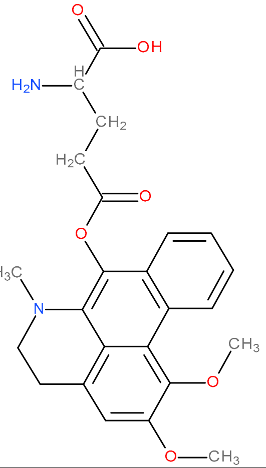	COC1=CC2=C3C(N(C)CC2)=C(OC(=O)CCC(N)C(O)=O)C2=C(C=CC=C2)C3=C1OC	438.48	$C_{24}H_{26}N_2O_6$	2-amino-5-({15,16-dimethoxy-10-methyl-10-azatetracyclo[7,7,1,02,7,013,17]heptadeca-1(16),2(7),3,5,8,13(17),14-heptaen-8-yl}oxy)-5-oxopentanoic acid	InChI=1S/C24H26N2O6/c1-26-11-10-13-12-17(30-2)23(31-3)20-14-6-4-5-7-15(14)22(21(26)19(13)20)32-18(27)9-8-16(25)24(28)29/h4-7,12,16H,8-11,25H2,1-3H3,(H,28,29)	PZJSTBHWUQTLGU-UHFFFAOYSA-N

No.	ID code	Structural formula	SMILES	Molecular weight	Molecular formula	IUPAC name	InChI String	InChI Key
117	5Ca2	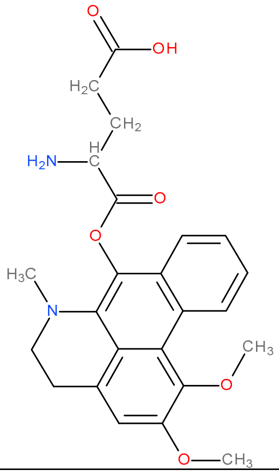	COC1=CC2=C3C(N(C)CC2)=C(OC(=O)C(N)CCC(O)=O)C2=C(C=CC=C2)C3=C1OC	438.48	$C_{24}H_{26}N_2O_6$	4-amino-5-({15,16-dimethoxy-10-methyl-10-azatetracyclo[7,7,1,02,7,013,17]heptadeca-1(16),2(7),3,5,8,13(17),14-heptaen-8-yl}oxy)-5-oxopentanoic acid	InChI=1S/C24H26N2O6/c1-26-11-10-13-12-17(30-2)23(31-3)20-14-6-4-5-7-15(14)22(21(26)19(13)20)32-24(29)16(25)8-9-18(27)28/h4-7,12,16H,8-11,25H2,1-3H3,(H,27,28)	JTIAHINYCCSWPN-UHFFFAOYSA-N
118	5Da1	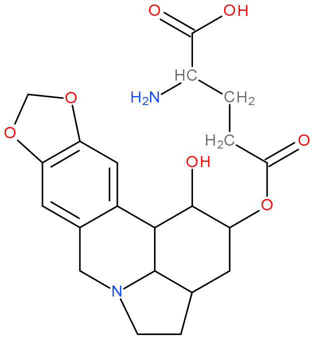	NC(CCC(=O)OC1CC2CCN3CC4=CC5=C(OCO5)C=C4C(C23)C1O)C(O)=O	418.446	$C_{21}H_{26}N_2O_7$	2-amino-5-({18-hydroxy-5,7-dioxa-12-azapentacyclo[10,6,1,02,10,04,8,015,19]nonadeca-2,4(8),9-trien-17-yl}oxy)-5-oxopentanoic acid	InChI=1S/C21H26N2O7/c22-13(21(26)27)1-2-17(24)30-16-5-10-3-4-23-8-11-6-14-15(29-9-28-14)7-12(11)18(19(10)23)20(16)25/h6-7,10,13,16,18-20,25H,1-5,8-9,22H2,(H,26,27)	ATUFDEQMZSWIBL-UHFFFAOYSA-N

No.	ID code	Structural formula	SMILES	Molecular weight	Molecular formula	IUPAC name	InChI String	InChI Key
119	5Da2	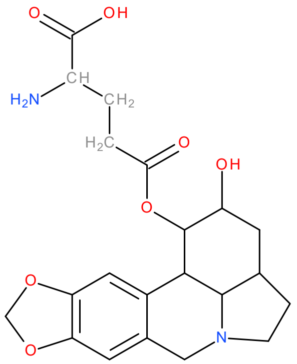	NC(CCC(=O)OC1C(O)CC2CCN3CC4=CC5=C(OCO5)C=C4C1C23)C(O)=O	418.446	$C_{21}H_{26}N_2O_7$	2-amino-5-({17-hydroxy-5,7-dioxa-12-azapentacyclo[10,6,1,02,10,04,8,015,19]nonadeca-2,4(8),9-trien-18-yl}oxy)-5-oxopentanoic acid	InChI=1S/C21H26N2O7/c22-13(21(26)27)1-2-17(25)30-20-14(24)5-10-3-4-23-8-11-6-15-16(29-9-28-15)7-12(11)18(20)19(10)23/h6-7,10,13-14,18-20,24H,1-5,8-9,22H2,(H,26,27)	NRUIPOVJHZPOOM-UHFFFAOYSA-N
120	5Da3	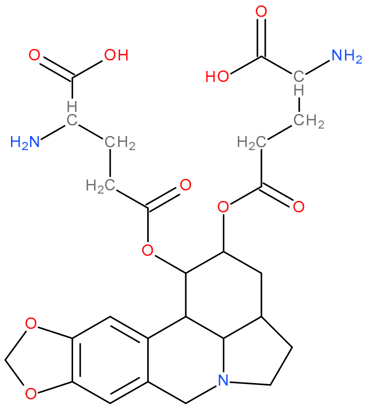	NC(CCC(=O)OC1CC2CCN3CC4=CC5=C(OCO5)C=C4C(C23)C1OC(=O)CCC(N)C(O)=O)C(O)=O	547.561	$C_{26}H_{33}N_3O_{10}$	2-amino-5-({17-[(4-amino-4-carboxybutanoyl)oxy]-5,7-dioxa-12-azapentacyclo[10,6,1,02,10,04,8,015,19]nonadeca-2,4(8),9-trien-18-yl}oxy)-5-oxopentanoic acid	InChI=1S/C26H33N3O10/c27-15(25(32)33)1-3-20(30)38-19-7-12-5-6-29-10-13-8-17-18(37-11-36-17)9-14(13)22(23(12)29)24(19)39-21(31)4-2-16(28)26(34)35/h8-9,12,15-16,19,22-24H,1-7,10-11,27-28H2,(H,32,33)(H,34,35)	XKANBAKCPFUGAT-UHFFFAOYSA-N
121	5Ea1	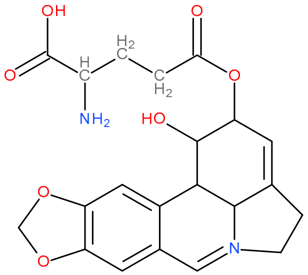	NC(CCC(=O)OC1C=C2CC[N+]3=CC4=CC5=C(OCO5)C=C4C(C23)C1O)C(O)=O	415.422	$C_{21}H_{23}N_2O_7$	17-[(4-amino-4-carboxybutanoyl)oxy]-18-hydroxy-5,7-dioxa-$12\lambda^5$-azapentacyclo[10,6,1,$0^{2,10}$,$0^{4,8}$,$0^{15,19}$]nonadeca-2,4(8),9,11,15-pentaen-12-ylium	InChI=1S/C21H22N2O7/c22-13(21(26)27)1-2-17(24)30-16-5-10-3-4-23-8-11-6-14-15(29-9-28-14)7-12(11)18(19(10)23)20(16)25/h5-8,13,16,18-20,25H,1-4,9,22H2/p+1	KFJAFKSOQSGACZ-UHFFFAOYSA-O

No.	ID code	Structural formula	SMILES	Molecular weight	Molecular formula	IUPAC name	InChI String	InChI Key
122	5Ea2	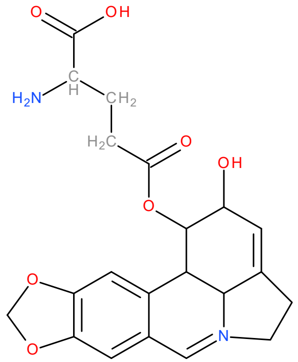	NC(CCC(=O)OC1C(O)C=C2CC[N+]3=CC4=CC5=C(OCO5)C=C4C1C23)C(O)=O	415.422	$C_{21}H_{23}N_2O_7$	18-[(4-amino-4-carboxybutanoyl)oxy]-17-hydroxy-5,7-dioxa-$12\lambda^{5}$-azapentacyclo[10,6,1,$0^{2,10}$,$0^{4,8}$,$0^{15,19}$]nonadeca-2,4(8),9,11,15-pentaen-12-ylium	InChI=1S/C21H22N2O7/c22-13(21(26)27)1-2-17(25)30-20-14(24)5-10-3-4-23-8-11-6-15-16(29-9-28-15)7-12(11)18(20)19(10)23/h5-8,13-14,18-20,24H,1-4,9,22H2/p+1	KYHZOCWPALHCTN-UHFFFAOYSA-O
123	5Ea3	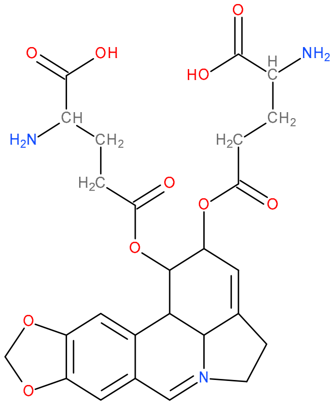	NC(CCC(=O)OC1C=C2CC[N+]3=CC4=CC5=C(OCO5)C=C4C(C23)C1OC(=O)CCC(N)C(O)=O)C(O)=O	544.537	$C_{26}H_{30}N_3O_{10}$	17,18-bis[(4-amino-4-carboxybutanoyl)oxy]-5,7-dioxa-12lambda5-azapentacyclo[10,6,1,02,10,04,8,015,19]nonadeca-2,4(8),9,11,15-pentaen-12-ylium	InChI=1S/C26H29N3O10/c27-15(25(32)33)1-3-20(30)38-19-7-12-5-6-29-10-13-8-17-18(37-11-36-17)9-14(13)22(23(12)29)24(19)39-21(31)4-2-16(28)26(34)35/h7-10,15-16,19,22-24H,1-6,11,27-28H2,(H-,32,33,34,35)/p+1	AKUOHJURJWGRBL-UHFFFAOYSA-O

Table S3. Structural and physicochemical properties. Protonation and electric charge.

ID code	Structural and physicochemical properties								Protonation					Electric charge
	HA	HAA	Fraction Csp3	RB	H-bond acceptors	H-bond donors	MR	TPSA	Acidic pKa	Basic pKa	pKa score	pI	Microspecies	Molar polarizability
1Aa1	10	0	0.6	4	5	2	31.91	89.62	1.87	8.6	8.6	5.23	3	12.92
1Aa2	10	0	0.6	4	5	2	31.91	89.62	3.48	7.48	7.48	5.47	3	12.92
1Aa3	11	0	0.67	5	5	2	36.72	89.62	2.01	8.6	8.6	5.31	3	14.73
1Aa4	11	0	0.67	5	5	2	36.72	89.62	3.61	7.48	7.48	5.55	3	14.73
1Aa5	11	0	0.67	5	5	2	36.72	89.62	2.02	9.51	9.51	5.77	3	14.73
1Aa6	11	0	0.67	5	5	2	36.72	89.62	3.93	7.41	7.41	5.56	3	14.73
1Aa7	12	0	0.71	6	5	2	41.53	89.62	2.13	9.51	9.51	5.83	3	16.54
1Aa8	12	0	0.71	6	5	2	41.53	89.62	4.04	7.41	7.41	5.64	3	16.54
1Ba1	10	0	0.6	4	4	3	33.54	106.41	2.15	9.31	9.31	5.73	4	13.32
1Ba2	10	0	0.6	4	3	3	34.67	112.2	-1.63	8.23	8.23	12	3	13.96
1Ba3	10	0	0.6	4	4	3	33.54	106.41	4.06	15.94	8.46	6.12	4	13.32
1Ca1	10	0	0.6	4	4	2	35.62	80.39	1.46	9.11	9.11	5.29	3	14.11
1Ca2	10	0	0.6	4	3	1	38.85	60.16	-	6.41	6.41	-	2	15.54
1Ca3	10	0	0.6	4	4	2	35.62	80.39	3.36	6.83	6.83	5.01	3	14.11
1Da1	11	0	0.4	4	6	2	32.11	106.69	1.4	6.51	6.51	3.96	3	13.11
1Da2	13	0	0.57	6	6	2	41.73	106.69	1.79	9.51	9.51	5.64	3	16.71
1Da3	13	0	0.57	6	6	2	41.73	106.69	3.71	7.39	7.39	5.5	3	16.71
1Da4	9	0	0.6	0	4	1	28.23	69.39	-	7.37	7.37	-	2	11.68
2Aa1	13	0	0.57	6	5	3	42.31	103.7	-1.8	15.41	-1.8	0.78	6	16.13
2Ba1	11	0	0.67	5	5	3	37.3	86.63	1.58	10.62	10.62	2.73	5	14.49
2Ba2	12	0	0.71	5	5	2	42.2	77.84	1.4	10.27	10.27	2.71	5	16.31
2Ba3	13	0	0.75	5	4	2	46.49	74.6	3.01	4.19	0	-	4	18.08
2Ba4	12	0	0.71	6	5	3	42.11	86.63	1.69	10.71	10.71	2.86	5	16.31
2Ba5	14	0	0.78	7	5	2	51.82	77.84	1.58	10.83	10.83	2.92	5	19.94

ID code	Structural and physicochemical properties								Protonation					Electric charge
	HA	HAA	Fraction Csp3	RB	H-bond acceptors	H-bond donors	MR	TPSA	Acidic pKa	Basic pKa	pKa score	pI	Microspecies	Molar polarizability
2Ba6	13	0	0.75	6	5	2	47.01	77.84	1,5	10.55	10.55	2.83	5	18.12
2Ba6a	16	0	0.78	9	5	2	61.41	77.84	1,4	7.38	7.38	2.75	5	23.78
2Ca1	10	0	0.6	4	5	3	30.85	94.83	3,28	14.29	-3.81	-	5	11.9
3Aa1	15	5	0.38	6	5	3	54.19	127.76	2,92	14	5.47	3.14	9	19.75
3Aa2	14	0	0.5	5	4	2	55.93	103.14	4,5	2.01	2.01	3.75	8	19.88
3Aa3	14	5	0.38	5	5	2	51.21	121.52	0,73	15.4	9.11	5.43	6	19.57
3Aa4	14	0	0.5	5	4	2	57.06	108.93	2,07	9.31	9.31	5.68	3	20.89
3Aa5	14	5	0.38	5	5	2	51.21	121.52	3,45	7.36	7.36	5.37	5	19.56
3Aa6	14	0	0.5	5	4	2	57.06	108.93	3,98	8.43	8.43	6.16	3	20.89
3Aa7	13	5	0.38	1	3	0	51.96	78.51	-	-0.7	-0.7	-	2	17.98
3Aa8	13	5	0.38	1	3	1	51.31	87.3	11,7	2.26	2.26	6.98	3	17.98
3Aa9	13	0	0.5	1	3	0	51.76	71.91	-	3.61	3.61	-	2	18.9
3Ab10	14	5	0.38	2	4	1	50.02	96.53	15,72	2.33	2.33	9.02	3	18.74
3Ba1	15	5	0.38	6	6	3	48.58	112.66	3,47	13.77	1.68	2.51	9	18
3Ba2	14	5	0.38	5	6	2	45.6	106.42	-0,71	15.08	9.11	5.36	6	17.79
3Ba3	14	5	0.38	5	6	2	45.6	106.42	-0,94	7.26	7.26	5.35	4	17.78
3Ba4	15	5	0.43	5	7	2	48.5	131.87	1,15	8.69	8.69	4.93	4	20.14
3Ba5	13	0	0.5	1	3	1	50.88	58.64	-	11.61	3.12	7.38	2	16.98
3Ba6	13	5	0.38	1	4	0	46.35	63.41	-	-	-2.46	-	1	16.2
3Bb7	13	0	0.5	1	4	0	45.25	55.84	-	-	2.23	-	-	16.58
3Bb8	14	5	0.38	2	5	1	44.41	81.43	-0,01	13.52	-0.01	6.75	3	16.97
4Aa1	22	6	0.47	10	4	2	87.59	77.84	3,94	1.67	1.67	2.74	8	32.85
4Aa2	21	6	0.53	10	3	2	88.53	66.56	1,5	9.53	9.53	6.07	5	33.68
4Aa3	15	0	0.89	9	4	2	62.34	66.56	2,41	9.53	9.53	8.11	5	24.62
4Ab1	15	0	0.5	7	7	3	45.43	150.36	3,02	14.36	-5.57	-	5	16.76

ID code	Structural and physicochemical properties								Protonation					Electric charge
	HA	HAA	Fraction Csp3	RB	H-bond acceptors	H-bond donors	MR	TPSA	Acidic pKa	Basic pKa	pKa score	pI	Microspecies	Molar polarizability
4Ab2	12	0	0.6	6	6	3	38.37	121.85	1.65	11.05	9.3	5.48	5	14.53
4Ac1	12	0	0.67	6	6	4	40.1	98.66	-0.69	9.07	9.07	2.02	11	15.84
4Ad1	14	0	1	6	7	1	48.95	129.52	_	5.15	5.15	_	2	21.5
4Ad2	14	0	1	6	7	1	48.95	129.52	_	8.5	8.5	_	2	21.5
4Ad3	16	0	1	8	7	1	58.57	129.52	_	9.47	9.47	_	2	25.11
4Ae1	12	0	0.6	0	6	2	36.06	59.08	0.25	3.55	3.55	_	3	22.12
4Ae2	12	0	0.6	0	6	2	33.69	59.08	0.24	3.55	3.55	_	3	21.47
4Ba1	12	0	0.4	6	7	2	33.59	115.92	1.27	6.52	6.52	3.91	3	13.9
4Ba2	13	0	0.5	6	7	2	38.01	115.92	1.42	6.52	6.52	3.97	3	15.69
4Ba3	10	0	0.6	5	5	1	31.43	78.62	_	7.09	7.09	_	2	13.15
4Ba4	14	0	0.57	8	7	2	43.2	115.92	1.66	9.51	9.51	5.57	3	17.48
4Ba5	15	0	0.62	8	7	2	47.62	115.92	1.54	9.51	9.51	5.52	3	19.29
4Ba6	15	0	0.5	5	6	0	49.01	89.98	_	_	-6.89	_	_	17.88
4Ba7	15	0	0.56	4	5	0	51.96	80.75	_	14.91	-7.02	_	2	18.91
4Ca1	10	0	0.6	4	4	4	35.04	93.41	3.78	5.97	10.06	7.9	5	13.09
4Ca2	10	0	0.6	5	4	4	35.04	93.41	4	5.97	10.06	8.08	5	13.09
4Ca3	10	0	0.6	4	4	3	31.65	86.63	3.95	0.34	-5.48	_	7	12.51
4Ca4	10	0	0.6	5	4	3	31.65	86.63	4	0.43	-5.46	_	7	12.51
4Cb1	10	0	0.6	3	4	4	32.93	93.41	3.47	11.39	9.92	7.57	5	12.4
4Cb2	11	0	0.6	3	5	5	35.67	119.43	1.59	27.64	9.52	8.45	8	13.92
4Cb3	11	0	0.6	4	5	5	39.65	115.35	2.48	11.38	9.65	7.39	9	13.92
4Cb4	11	0	0.6	3	5	4	39.74	96.42	3.6	10.05	8.15	6.95	5	13.92
4Cb5	11	0	0.67	3	4	4	37.74	93.41	3.84	11.54	9.98	7.84	5	14.2
4Cb6	12	0	0.67	3	5	5	40.48	119.43	1.94	11.5	9.96	10.2	7	15.73
4Cb7	11	0	0.6	3	5	5	39.65	105.44	2.32	10.38	8.56	9.37	9	13.92

ID code	Structural and physicochemical properties								Protonation					Electric charge
	HA	HAA	Fraction Csp3	RB	H-bond acceptors	H-bond donors	MR	TPSA	Acidic pKa	Basic pKa	pKa score	pI	Microspecies	Molar polarizability
4Da1	11	0	0.6	4	5	3	42.5	136.33	1.44	9.48	9.48	6.9	5	16.44
4Da2	11	0	0.8	4	5	3	41.24	136.33	1.89	9.32	9.32	5.88	7	16.92
4Da3	11	0	0.8	4	5	3	41.24	136.33	1.89	9.39	9.39	5.92	7	16.92
4Da4	12	0	0.8	5	6	4	43.87	132.85	-1.94	9.53	9.53	4.12	5	17.45
4Da5	12	0	0.8	4	6	4	43.87	132.85	-1.97	9.51	9.51	4.11	5	17.45
4Da6	12	0	0.8	4	6	4	43.87	132.85	-1.96	9.58	9.58	4.12	5	17.45
4Da7	11	0	0.8	2	5	3	40.22	112.62	1.52	7.72	7.72	5	5	16.22
4Da8	11	0	0.8	2	5	2	44.29	89.61	3.53	2.92	2.92	3.18	4	16.22
4Da9	11	0	0.8	2	5	3	44.23	108.54	3.51	2.9	2.9	3.15	8	16.22
4Da10	12	0	0.83	2	5	3	45.03	112.62	1.77	9.1	9.1	6.06	5	18.03
4Da11	11	0	0.8	2	5	3	44.19	98.63	2.5	5.87	5.87	4.68	7	16.22
4Db1	11	0	0.8	4	5	3	42.69	120.01	2.13	6.41	6.41	1.76	6	15.22
4Db2	11	0	0.8	2	5	3	38.38	110.43	1.49	9.58	9.58	1.97	5	15.22
4Db3	11	0	0.8	2	5	3	42.35	96.44	-0.33	9.24	9.24	0.8	4	15.22
4Db4	12	0	0.83	2	5	3	43.18	110.43	1.75	9.58	9.58	2.17	5	17.02
4Db5	12	0	0.83	2	5	3	47.16	96.44	1.25	12	12	2.52	5	17.02
4Db6	13	0	0.86	2	5	3	51.96	96.44	1.62	10.42	10.42	2.01	5	18.83
4Dc1	14	0	1	6	7	5	50.83	160.7	-0.25	10.24	10.24	1.26	11	20.24
4Dc2	14	0	1	6	7	5	50.83	160.7	1.34	10.71	10.71	1.63	11	20.24
4Dc3	14	0	1	6	7	5	50.83	160.7	1.49	10.49	10.49	1.66	8	20.24
4Dc4	12	0	1	4	5	3	48.51	120.24	0.02	9.94	9.94	1.36	5	19.13
4Dc5	12	0	1	4	5	3	48.51	120.24	1.39	10.79	10.79	1.72	4	19.13
4Dc6	17	0	0.8	5	10	7	53.33	210.03	-0.01	28.81	8.81	0.75	17	21.87
4Dc7	17	0	0.8	5	10	7	57.3	196.04	0.93	7.03	7.03	1.31	16	21.87
4Dc8	15	0	1	4	8	6	55.53	158.74	0.94	9.23	9.23	2.54	11	21.07

ID code	Structural and physicochemical properties								Protonation					Electric charge
	HA	HAA	Fraction Csp3	RB	H-bond acceptors	H-bond donors	MR	TPSA	Acidic pKa	Basic pKa	pKa score	pI	Microspecies	Molar polarizability
4Dd1.1	10	0	0.8	5	5	4	34.16	95.58	2.35	9.53	9.53	7.29	5	14.21
4Dd1.2	10	0	0.8	3	5	4	32.08	95.58	2.02	8.76	8.76	6.21	5	13.51
4Dd1.3	10	0	0.8	2	5	4	32.08	95.58	1.35	15.63	8.47	5.46	6	13.51
4Dd1.4	10	0	0.8	3	5	4	36.05	81.59	1.6	9.97	9.97	6.5	5	13.51
4Dd2.1	12	0	0.8	6	6	3	38.99	116.22	-0.81	13.62	9.53	5.67	6	15.31
4Dd3.1	9	0	0.8	1	4	2	29.6	72.55	3.22	9.64	9.64	6.39	3	12.05
4Dd4.1	21	0	0.75	9	7	6	74.44	161.98	3.38	14.92	8.44	5.92	15	28.04
4Dd4.2	22	0	0.77	10	7	6	79.25	161.98	3.48	15.64	8.44	5.97	13	29.85
4Dd4.3	23	0	0.69	11	8	6	79.86	179.05	3.18	13.63	8.44	3.61	12	29.85
4Dd4.m	14	0	0.75	5	5	4	51.05	112.65	2.07	12.05	9.52	5.8	7	18.76
5Aa1	35	13	0.4	10	10	3	127.12	140.62	0.99	10.11	10.11	2.4	5	48.58
5Aa2	33	13	0.4	5	7	0	125.87	91.37	-	-	-4.22	-	-	46.6
5Aa3	38	6	0.44	12	10	3	137.75	155.64	-1.16	15.1	9.51	5.61	6	53.72
5Aa4	38	6	0.44	12	10	3	137.75	155.64	-1.16	15.16	7.41	5.54	5	53.72
5Ba1	55	24	0.4	16	12	2	216.4	142.25	1.33	9.54	9.54	8.95	9	81.6
5Ba2	55	24	0.4	16	12	2	216.4	142.25	3.14	8.44	8.44	8.1	9	81.6
5Ca1	32	14	0.33	8	7	2	124.83	111.32	1.4	9.51	9.51	5.9	5	48.21
5Ca2	32	14	0.33	8	7	2	124.83	111.32	3.8	7.25	7.25	5.55	5	48.21
5Da1	30	6	0.62	6	9	3	107.52	131.55	1.79	13.78	9.72	9.3	6	41.44
5Da2	30	6	0.62	6	9	3	107.52	131.55	1.72	14.14	9.64	9.22	6	41.44
5Da3	39	6	0.62	12	13	4	136.15	200.94	1.4	9.94	9.94	9.15	11	52.94
5Ea1	30	6	0.48	6	8	3	108.3	131.32	1.72	13.53	9.51	11.5	4	40.68
5Ea2	30	6	0.48	6	8	3	108.3	131.32	1.66	13.73	9.51	11.6	4	40.68
5Ea3	39	6	0.5	12	12	4	136.94	200.71	1.34	9.81	9.81	9.51	7	52.18

Table S4. Water solubility.

Two topological methods for predicting aqueous solubility have been developed, one of them being implemented after ESOL (*Delaney JS. (2004))* [23] and the other being taken from *Ali J. et al. (2012)* [24]. The two methods differ in the general solubility equation, but both exclude the melting point and show a linear correlation between the predicted and the experimental values (R^2=0,69, respectively 0,81). Another method was developed by SILICOS-IT (fragmentary method calculated using FILTER-IT 1.0.2 program), whose correlation coefficient was corrected by the molecular weight and (R^2=0,75). All three methods calculate the decimal logarithm of molar water solubility (logS), but the results can be expressed in mol/l şi mg/ml [11].

No.	ID code	logS AquaSol	ESOL Log S	ESOL Solub. (mg/ml)	ESOL Solub. (mol/l)	ESOL Class	Ali Log S	Ali Sol (mg/ml)	Ali Sol (mol/l)	Ali Class	Silicos-IT LogSw	Silicos-IT Sol. (mg/ml)	Silicos-IT Sol. (mol/l)	Silicos-IT class
1	1Aa1	-0.19	1.04	1620	11	H	1.09	1800	12.3	H	0.6	580	3.94	S
2	1Aa2	-0.06	1.74	8120	55.2	H	2.24	25600	174	H	0.6	580	3.94	S
3	1Aa3	-0.37	0.79	1010	6.24	H	0.71	836	5.19	H	0.18	244	1.52	S
4	1Aa4	-0.25	1.49	4960	30.8	H	1.86	11600	71.8	H	0.18	244	1.52	S
5	1Aa5	-0.44	1.61	6530	40.5	H	2.05	18200	113	H	0.18	244	1.52	S
6	1Aa6	-0.31	1.61	6530	40.5	H	2.05	18200	113	H	0.18	244	1.52	S
7	1Aa7	-0.63	1.36	4010	22.9	H	1.68	8380	47.8	H	-0.23	103	0.59	S
8	1Aa8	-0.5	1.25	3090	17.6	H	1.49	5450	31.1	H	-0.23	103	0.59	S
9	1Ba1	-0.51	1.5	4650	31.8	H	1.48	4440	30.4	H	0.68	698	4.78	S
10	1Ba2	-0.75	0.48	440	3.03	H	-0.33	67.9	0.47	V	0.46	423	2.91	S
11	1Ba3	-0.51	2.25	26100	179	H	2.72	76300	522	H	0.68	698	4.78	S
12	1Ca1	-1.36	1.1	2110	12.7	H	1.57	6190	37.4	H	-0.31	81.1	0.49	S
13	1Ca2	-1.54	-1.18	12.1	0.07	V	-1.58	4.81	0.03	V	-1.51	5.7	0.03	S
14	1Ca3	-0.78	1.1	2110	12.7	H	1.57	6190	37.4	H	-0.31	81.1	0.49	S
15	1Da1	-0.89	1.63	6870	42.7	H	1.84	11200	69.2	H	1.04	1770	11	S
16	1Da2	-0.96	1.64	8230	43.5	H	1.92	15900	83.8	H	0.22	313	1.66	S
17	1Da3	-0.84	1.53	6340	33.5	H	1.74	10300	54.5	H	0.22	313	1.66	S
18	1Da4	-0.1	-0.07	111	0.86	V	-0.06	111	0.86	V	-0.16	89.7	0.7	S

No.	ID code	logS AquaSol	ESOL Log S	ESOL Solub. (mg/ml)	ESOL Solub. (mol/l)	ESOL Class	Ali Log S	Ali Sol (mg/ml)	Ali Sol (mol/l)	Ali Class	Silicos-IT LogSw	Silicos-IT Sol. (mg/ml)	Silicos-IT Sol. (mol/l)	Silicos-IT class
19	2Aa1	-0.9	0.53	641	3.39	H	0.16	273	1.44	H	0.12	252	1.33	S
20	2Ba1	-0.33	1.38	3870	24	H	1.74	8910	55.3	H	0.09	196	1.22	S
21	2Ba2	-0.35	1	1740	9.95	H	1.44	4820	27.5	H	0.41	446	2.55	S
22	2Ba3	-0.2	-0.54	55.1	0.29	V	-0.87	25.7	0.14	V	-0.7	37.5	0.2	S
23	2Ba4	-0.45	1.13	2350	13.4	H	1.36	4000	22.8	H	-0.33	82.7	0.47	S
24	2Ba5	-0.72	0.5	637	3.13	H	0.68	977	4.81	H	-0.41	78.7	0.39	S
25	2Ba6	-0.44	0.75	1060	5.62	H	1.07	2200	11.6	H	0	187	0.99	S
26	2Ba6a	-1.73	-0.08	225	0.83	V	0.22	44	1.64	H	-1.67	5.76	0.02	S
27	2Ca1	-0.05	0.12	194	1.31	H	-0.54	43.1	0.29	V	1.11	1900	12.9	S
28	3Aa1	-1.92	-1.59	5.91	0.03	V	-3.01	0.22	0	S	-0.96	25.3	0.11	S
29	3Aa2	-1.36	-1.17	14.8	0.07	V	-2.23	1.29	0.01	S	0.7	1080	4.99	S
30	3Aa3	-1.56	0.37	497	2.32	H	0.31	442	2.06	H	-1.12	16.1	0.08	S
31	3Aa4	-1.61	1.07	2540	11.8	H	1.33	4580	21.2	H	0.48	656	3.03	S
32	3Aa5	-1.52	0.37	497	2.32	H	0.31	442	2.06	H	-1.12	16.1	0.08	S
33	3Aa6	-1.6	1.07	2540	11.8	H	1.33	4580	21.2	H	0.48	656	3.03	S
34	3Aa7	-2.08	-1.55	5.58	0.03	V	-1.65	4.43	0.02	V	-1.8	3.11	0.02	S
35	3Aa8	-1.79	-1.33	9.27	0.05	V	-1.47	6.69	0.03	V	-2.29	1.02	0.01	S
36	3Aa9	-1.45	-1.52	6.02	0.03	V	-1.9	2.5	0.01	V	-0.37	85	0.43	S
37	3Ab10	-2.02	-196	2.32	0.01	V	-2.69	0.44	0	S	-2.03	1.99	0.01	S
38	3Ba1	-1.32	-1.11	16.8	0.08	V	-2.06	1.86	0.01	S	-0.9	26.8	0.13	S
39	3Ba2	-1.1	0.86	1420	7.17	H	1.28	3730	18.8	H	-1.06	17.1	0.09	S
40	3Ba3	-1.06	0.86	1420	7.17	H	1.28	3730	18.8	H	-1.06	17.1	0.09	S
41	3Ba4	-1.12	0.89	1810	7.74	H	1.14	3200	13.6	H	-1.04	21.2	0.09	S
42	3Ba5	-1.15	-0.71	35.7	0.2	V	-0.46	63	0.35	V	-0.31	90.1	0.49	S
43	3Ba6	-1.57	-1.06	15.8	0.09	V	-0.69	37.2	0.21	V	-1.74	3.29	0.02	S

No.	ID code	logS AquaSol	ESOL Log S	ESOL Solub. (mg/ml)	ESOL Solub. (mol/l)	ESOL Class	Ali Log S	Ali Sol (mg/ml)	Ali Sol (mol/l)	Ali Class	Silicos-IT LogSw	Silicos-IT Sol. (mg/ml)	Silicos-IT Sol. (mol/l)	Silicos-IT class
44	3Bb7	-1.36	-1.08	15.2	0.08	V	-1	18.1	0.1	V	0.01	185	1.01	S
45	3Bb8	-1.59	-1.48	6.52	0.03	V	-1.74	3.58	0.02	V	-1.97	2.1	0.01	S
46	4Aa1	-3.33	-3.17	0.24	0	S	-3.86	0.05	0	S	-4.15	0.02	0	M
47	4Aa2	-3.43	-2.05	2.97	0.01	S	-1.92	3.97	0.01	V	-4.83	0	0	M
48	4Aa3	-1.59	0.58	971	3.78	H	1.39	6250	24.3	H	-2.35	1.15	0	S
49	4Ab1	-1.98	-0.04	201	0.92	V	-1.56	6.08	0.03	V	1.63	9310	42.5	S
50	4Ab2	-1.42	2.14	23900	136	H	2.28	33300	190	H	0.3	347	1.98	S
51	4Ac1	-0.69	2.94	152000	861	H	4.09	2190000	12400	H	0.03	187	1.06	S
52	4Ad1	-1.15	-0.13	185	0,75	V	-0.87	33.3	0.14	V	-0.49	79.8	0.32	S
53	4Ad2	-0.98	0.16	355	1.43	H	-0.4	97.7	0.4	V	-0.49	79.8	0.32	S
54	4Ad3	-1.37	-0.33	128	0.47	V	-1.14	19.9	0.07	V	-1.29	14	0.05	S
55	4Ae1	-	-1.27	19.1	0.05	V	0.52	1180	3.28	H	-1.18	23.9	0.07	S
56	4Ae2	-	-1.42	13.6	0.04	V	0.26	644	1.8	H	-1.16	24.8	0.07	S
57	4Ba1	-0.97	1.52	5920	33.4	H	1.42	4640	26.2	H	1.27	3340	18.8	S
58	4Ba2	-0.93	1.44	5310	27.8	H	1.43	5130	26.8	H	0.88	1460	7.64	S
59	4Ba3	-0.2	0.09	180	1.22	H	-0.36	64	0.44	V	-0.07	124	0.84	S
60	4Ba4	-1.19	1.53	7000	34.1	H	1.5	6510	31.7	H	0.46	588	2.86	S
61	4Ba5	-1.25	1.46	6300	28.7	H	1.52	7290	33.3	H	0.07	257	1.17	S
62	4Ba6	-1.78	-0.65	48.3	0.23	V	-1.12	16.4	0.08	V	-0.17	144	0.67	S
63	4Ba7	-1.6	-0.39	86.1	0.4	V	-0.42	81.7	0.38	V	-0.82	32.3	0.15	S
64	4Ca1	-0.74	0.06	168	1.15	H	-0.62	35	0.24	V	0.36	332	2.28	S
65	4Ca2	-0.75	0.26	265	1.81	H	-0.4	57.9	0.4	V	-0.02	141	0.96	S
66	4Ca3	-0.57	0.11	190	1.29	H	-0.38	60.7	0.41	V	0.5	466	3.17	S
67	4Ca4	-0.6	0.31	299	2.04	H	-0.17	100	0.68	V	0.13	197	1.34	S
68	4Cb1	-0.66	0.24	250	1.74	H	-0.24	83.6	0.58	V	0.83	977	6.78	S

No.	ID code	logS AquaSol	ESOL Log S	ESOL Solub. (mg/ml)	ESOL Solub. (mol/l)	ESOL Class	Ali Log S	Ali Sol (mg/ml)	Ali Sol (mol/l)	Ali Class	Silicos-IT LogSw	Silicos-IT Sol. (mg/ml)	Silicos-IT Sol. (mol/l)	Silicos-IT class
69	4Cb2	-0.49	1.88	12000	75.6	H	2.07	18700	118	H	0.96	1450	9.1	S
70	4Cb3	-0.69	2.06	18500	116	H	2.35	35900	226	H	0.64	698	4.39	S
71	4Cb4	-0.83	1.66	7240	45.5	H	2.19	24700	155	H	1.37	3770	23.7	S
72	4Cb5	-0.82	-0.07	133	0.84	V	-0.61	38.8	0.25	V	0.54	550	3.48	S
73	4Cb6	-0.62	1.91	14100	81.6	H	2.27	32100	185	H	0.67	814	4.7	S
74	4Cb7	-0.76	1.73	8500	53.4	H	2.12	20800	130	H	0.77	931	5.85	S
75	4Da1	-0.86	1.8	11100	62.4	H	1.66	8220	46.1	H	0.14	245	1.37	S
76	4Da2	-0.74	1.01	1850	10.3	H	0.39	438	2.44	H	0.15	254	1.42	S
77	4Da3	-0.62	0.99	1750	9.74	H	0.35	398	2.22	H	0.15	254	1.42	S
78	4Da4	-0.88	1.35	4420	22.5	H	1.08	2380	12.1	H	0.34	432	2.2	S
79	4Da5	-0.85	0.72	1030	5.24	H	0.15	277	1.41	H	0.72	1020	5.21	S
80	4Da6	-0.75	0.72	1030	5.24	H	0.15	277	1.41	H	0.72	1020	5.21	S
81	4Da7	0.03	0.53	602	3.38	H	0.29	351	1.97	H	0.06	206	1.15	S
82	4Da8	-0.48	0.31	368	2.06	H	0.42	474	2.66	H	0.48	534	3	S
83	4Da9	0.06	0.49	552	3.1	H	0.32	370	2.08	H	-0.46	62.2	0.35	S
84	4Da10	-0.05	0.57	711	3.7	H	0.5	610	3.17	H	-.0.22	115	0.6	S
85	4Da11	-0.47	0.38	432	2.42	H	0.35	398	2.23	H	-0.13	132	0.74	S
86	4Db1	0.05	1.84	12400	69.2	H	2.09	22000	123	H	0.35	405	2.26	S
87	4Db2	0.41	1.78	10900	60.7	H	2.42	46600	260	H	0.69	873	4.88	S
88	4Db3	0.32	1.64	7790	43.5	H	2.47	52900	296	H	0.5	562	3.14	S
89	4Db4	0.16	1.82	12600	65.5	H	2.61	79200	410	H	0.4	490	2.54	S
90	4Db5	-0.01	1.61	7840	40.6	H	2.56	70800	366	H	0.21	315	1.63	S
91	4Db6	-0.28	1.3	4140	20	H	2.2	32900	159	H	-0.07	177	0.85	S
92	4Dc1	-0.37	2.43	66800	270	H	2.69	120000	488	H	1.07	2900	11.7	S
93	4Dc2	-0.3	2.72	130000	527	H	3.17	362000	1460	H	1.07	2900	11.7	S

No.	ID code	logS AquaSol	ESOL Log S	ESOL Solub. (mg/ml)	ESOL Solub. (mol/l)	ESOL Class	Ali Log S	Ali Sol (mg/ml)	Ali Sol (mol/l)	Ali Class	Silicos-IT LogSw	Silicos-IT Sol. (mg/ml)	Silicos-IT Sol. (mol/l)	Silicos-IT class
94	4Dc3	-0.28	2.72	130000	527	H	3.17	362000	1460	H	1.07	2900	11.7	S
95	4Dc4	-0.41	2.33	46500	216	H	3.27	399000	1850	H	-0.05	193	0.9	S
96	4Dc5	-0.27	2.62	89300	415	H	3.73	1170000	5430	H	-0.05	193	0.9	S
97	4Dc6	-0.17	4.3	5770000	19900	H	5.27	-	188000	H	2.69	141000	486	S
98	4Dc7	-0.66	4.15	4070000	14000	H	5.32	-	208000	H	2.5	90700	313	S
99	4Dc8	-0.14	3.81	1670000	6410	H	5.34	-	221000	H	1.35	5820	22.4	S
100	4Dd1.1	-0.32	2.34	32200	217	H	3	147000	993	H	0.39	360	2.43	S
101	4Dd1.2	0.3	1.67	6830	46.7	H	2.09	18200	124	H	0.66	663	4.54	S
102	4Dd1.3	0.17	1.16	2090	14.3	H	1.36	3330	22.8	H	0.78	887	6.07	S
103	4Dd1.4	0.03	1.81	9390	64.3	H	2.62	60400	413	H	0.47	427	2.92	S
104	4Dd2.1	-1.29	1.46	5120	28.9	H	1.31	3600	20.3	H	1.13	2400	13.5	S
105	4Dd3.1	0.29	1.15	1860	14.2	H	1.79	8070	61.6	H	0.75	743	5.66	S
106	4Dd4.1	-1.37	1.94	26500	87.4	H	2.1	38300	126	H	-0.24	175	0.58	S
107	4Dd4.2	-1.48	1.7	15900	50.1	H	1.74	17300	54.7	H	-0.64	73	0.23	S
108	4Dd4.3	-1.9	1.84	22800	68.7	H	1.64	14400	43.5	H	-0.55	93.8	0.28	S
109	4Dd4.m	-0.31	1.76	11500	57.1	H	2.23	34700	171	H	-0.35	90.7	0.45	S
110	5Aa1	-3.52	-1.58	12.9	0.03	V	-1.02	46.5	0.1	V	-5.84	0	0	M
111	5Aa2	-3.97	-3.44	0.16	0	S	-2.84	0.66	0	S	-6.72	0	0	P
112	5Aa3	-3.52	-1.64	12.3	0.02	V	-1.47	18	0.03	V	-4.21	0.03	0	M
113	5Aa4	-3.48	-1.64	12.3	0.02	V	-1.47	18	0.03	V	-4.21	0.03	0	M
114	5Ba1	-4.32	-5.99	0	0	M	-6.18	0	0	P	-10.69	0	0	I
115	5Ba2	-4.29	-5.99	0	0	M	-6.18	0	0	P	-10.69	0	0	I
116	5Ca1	-3.45	-2.97	0.48	0	S	-2.9	0.56	0	S	-5.73	0	0	M
117	5Ca2	-3.39	-2.97	0.48	0	S	-2.9	0.56	0	S	-5.73	0	0	M
118	5Da1	-2.67	-0.94	48.2	0.12	V	-0.26	230	0.55	V	-1.72	8.03	0.02	S

No.	ID code	logS AquaSol	ESOL Log S	ESOL Solub. (mg/ml)	ESOL Solub. (mol/l)	ESOL Class	Ali Log S	Ali Sol (mg/ml)	Ali Sol (mol/l)	Ali Class	Silicos-IT LogSw	Silicos-IT Sol. (mg/ml)	Silicos-IT Sol. (mol/l)	Silicos-IT class
119	5Da2	-2.57	-0.94	48.2	0.12	V	-0.26	230	0.55	V	-1.72	8.03	0.02	S
120	5Da3	-3.11	0.41	1410	2.57	H	1.12	7160	13.1	H	-1.71	10.6	0.02	S
121	5Ea1	-2.88	-0.16	289	0.7	V	1	4170	10	H	-2.18	2.73	0.01	S
122	5Ea2	-2.83	-0.16	289	0.7	V	1	4170	10	H	-2.18	2.73	0.01	S
123	5Ea3	-3.24	1.19	8470	15.5	H	2.38	130000	238	H	-2.18	3.59	0.01	S

Table S5. Lipophilicity - partition coefficients.

The n-octanol and water partition coefficient (LogP) is the classical descriptor of the lipophilicity of a compound (at a pH value at which all the molecules of the compound are in undissociated form). Most computational methods for estimating logP use multiple predictors, either to select the most accurate method, or to generate an „consensus" estimation [11]:

- **XLOGP3 –** an atomistic method (calculated with XLOGP program, version 3.2.2, CCBG, Shanghai Institute of Organic Chemistry), which includes correction factors and an information library [11, 25-27];
- **WLOGP** – a purely atomistic method based on the fragmentation system *Wildman şi Crippen (1999)* [28];
- **MLOGP** – a subtype of topologycal method (implemented by *Moriguchi I. et al. (1992), Moriguchi I. et al. (1994), Lipinski PA. et al. (2001)*) based on a linear relationship with 13 molecular descriptors [29,30];
- **SILICOS-IT** – a hybrid method based on 27 fragments and 7 topologycal descriptors (calculated with FILTER-IT, version 1.0.2, SILICOS-IT, *http: www.//silicos-it.com*);
- **iLOGP** is an „in-house physics-based" method implemented by *Daina A. et al. (2014)*, which is based on the solvation free energies in n-octanol and water, calculated according to the GB/SA model (*Generalized-Born and solvent accessible surface area*) [11];
- **Consensus logP** represents the arithmetic mean of the values calculated by the 5 proposed methods [11].

ID code	iLOGP	XLOGP	WLOGP	MLOGP	Silicos-IT Log P	Consensus Log P	logP	logD at pH 7,4	HLB
1Aa1	0.58	-2.43	-1.04	-3.18	-1.04	-1.42	-3.386	-3.36	18.92
1Aa2	0.62	-3.54	-1.04	-0.96	-1.04	-1.19	-1.22	-4.11	18.92
1Aa3	0.9	-2.07	-0.65	-2.8	-0.68	-1.06	-2.963	-3.62	17.96
1Aa4	0.83	-3.17	-0.65	-0.58	-0.68	-0.85	-0.797	-3.75	17.96
1Aa5	1.18	-3.36	-0.65	-2.8	-0.68	-1.26	-3.028	-3.7	18.01
1Aa6	0.95	-3.36	-0.65	-0.58	-0.68	-0.87	-0.862	-3.86	18.01
1Aa7	1.26	-3	-0.26	-2.45	-0.3	-0.95	-2.605	-3.36	17.15
1Aa8	1.4	-2.82	-0.26	-0.23	-0.3	-0.44	-0.439	-3.49	17.15
1Ba1	0.43	-3.15	-1.34	-3.58	-1.42	-1.81	-4.077	-4.59	22.35

ID code	iLOGP	XLOGP	WLOGP	MLOGP	Silicos-IT Log P	Consensus Log P	logP	logD at pH 7,4	HLB
1Ba2	-0.2	-1.52	-1.94	-1.77	-1.66	-1.42	-2.639	-3.52	26.72
1Ba3	0.26	-4.34	-1.34	-1.37	-1.42	-1.64	-1.911	-4.61	22.35
1Ca1	0.91	-2.71	-0.06	-2.78	-0.07	-0.94	-2.688	-3.26	15.18
1Ca2	1.06	0.74	0.62	-0.17	1.05	0.66	0.139	0.01	12.68
1Ca3	0.58	-2.71	-0.06	-0.57	-0.07	-0.56	-0.522	-3.68	15.18
1Da1	0.5	-3.5	-1.51	-3.27	-1.37	-1.83	-2.752	-4.67	20.66
1Da2	0.57	-3.58	-0.73	-2.54	-0.67	-1.39	-2.578	-3.95	19.02
1Da3	0.84	-3.4	-0.73	-0.33	-0.67	-0.86	-0.412	-4.14	19.02
1Da4	0.44	-0.91	-0.82	-0.52	0.12	-0.34	-0.483	-1.23	18.13
2Aa1	0.42	-1.82	-0.56	-0.73	-0.75	-0.69	-0.907	-7.62	17.4
2Ba1	0.85	-3	-0.48	-2.8	-0.76	-1.24	-0.793	-6.41	17.83
2Ba2	0.88	-2.53	-0.13	-0.23	-0.83	-0.57	-0.554	-6.18	17.65
2Ba3	-2.71	-0.24	0.01	-3.58	-0.76	-1.46	-0.745	-7.17	17.75
2Ba4	0.86	-2.63	-0.09	-2.45	-0.38	-0.94	-0.37	-5.99	16.97
2Ba5	1.4	-1.8	0.65	0.42	-0.03	0.13	0.292	-5.36	16.08
2Ba6	1.21	-2.17	0.26	0.1	-0.43	-0.21	-0.131	-5.77	16.83
2Ba6a	1.42	-1.35	1.08	1.02	1.15	0.66	0.976	-4.88	15.46
2Ca1	0.18	-0.97	-0.7	-0.96	-0.95	-0.68	-1.116	-7.54	13.49
3Aa1	0.88	0.75	0.68	-2.87	0.79	0.05	-0.377	-5.62	11.93
3Aa2	0.74	0.49	0.4	-0.39	-0.27	0.19	0.817	-5.71	18.4
3Aa3	1.05	-2.33	0.52	-3.55	1.29	-0.6	-3.165	-3.71	16.25
3Aa4	1.52	-3.05	-0.2	-3.01	-0.51	-1.05	-2.768	-3.34	22.77
3Aa5	0.8	-2.33	0.52	-1.33	1.29	-0.21	-0.999	-3.93	16.25
3Aa6	1.01	-3.05	-0.2	-0.79	-0.51	-0.71	-0.602	-3.36	22.77
3Aa7	1.36	0.43	0.81	0.58	2.09	1.05	-0.332	0.51	14.24
3Aa8	1.13	0.08	0.28	-0.1	2.25	0.73	-0.917	-0.04	14.28

ID code	iLOGP	XLOGP	WLOGP	MLOGP	Silicos-IT Log P	Consensus Log P	logP	logD at pH 7,4	HLB
3Aa9	1.17	0.81	0.32	0.44	0.77	0.7	1.088	0.58	18.66
3Ab10	0.94	1.07	0.6	0.15	1.77	0.91	0.323	0.54	12.17
3Ba1	0.57	0.14	0.21	-3.27	-0.46	-0.56	-0.627	-7.44	12.31
3Ba2	0.79	-2.95	0.05	-3.95	0.04	-1.2	-3.527	-4.56	16.61
3Ba3	0.74	-2.95	0.05	-1.74	0.04	-0.77	-1.361	-4.82	16.61
3Ba4	0.56	-3.33	0.33	-4.09	-0.9	-1.49	-3.781	-5.08	17.26
3Ba5	1.43	-0.31	-1.21	-0.39	-0.11	-0.12	0.272	-0.57	21.32
3Ba6	1.32	-0.19	0.34	0.15	0.84	0.49	-0.591	-0.17	14.61
3Bb7	1.19	0.27	-0.4	0.02	0.21	0.26	0.803	0.08	18.57
3Bb8	0.81	0.46	0.13	-0.25	0.52	0.33	0.073	0.08	12.58
4Aa1	2.1	2.58	3	2.58	2.98	2.65	1.951	-2.45	7.67
4Aa2	2.48	0.94	2.71	0.46	3.16	1.95	0.572	0.79	11.56
4Aa3	2.08	-2.25	0.96	-1.07	1.29	0.2	-1.148	-1.89	19.38
4Ab1	0.36	-1.11	-0.63	-1.11	-2.2	-0.94	-1.779	-7.73	18.48
4Ab2	0.87	-4.23	-1.02	-3.67	-1.22	-1.86	-3.447	-4.05	22.6
4Ac1	0.67	-5.51	-0.97	-2.8	-1.44	-2.01	-0.033	-6.56	23.59
4Ad1	0.99	-1.35	0.78	-0.73	-1.8	-0.42	-2.192	-1.35	24.12
4Ad2	1.33	-1.8	0.43	-1.14	-1.8	-0.6	-2.687	-2.99	24.57
4Ad3	1.94	-1.09	1.21	-0.41	-1.19	0.09	-1.971	-3.4	23.19
4Ae1	0	-1.26	0.82	-2.17	0.88	-0.35	0	-0.98	24.56
4Ae2	0	-1.01	0.36	-2.17	0.86	-0.39	0	-0.59	24.6
4Ba1	0.42	-3.28	-1.93	-3.67	-1.55	-2	-3.27	-4.48	21.41
4Ba2	0.4	-3.29	-1.54	-3.29	-1.38	-1.82	-3.034	-4.36	20.5
4Ba3	1.35	-0.81	-0.99	-0.55	-0.72	-0.35	-0.695	-1.16	19.05
4Ba4	1.1	-3.36	-1.15	-2.94	-0.88	-1.44	-3.096	-3.75	19.75
4Ba5	0.67	-3.38	-0.76	-2.6	-0.66	-1.35	-2.86	-3.64	18.98

ID code	iLOGP	XLOGP	WLOGP	MLOGP	Silicos-IT Log P	Consensus Log P	logP	logD at pH 7,4	HLB
4Ba6	1.19	-0.31	-0.59	-0.46	-0.23	-0.08	-0.497	-0.1	16.58
4Ba7	1.11	-0.8	-0.77	-0.16	0.39	-0.04	-0.897	-1.11	15.71
4Ca1	0.36	-0.86	0.05	-0.15	-0.99	-0.32	-0.291	-2.31	17.08
4Ca2	0.5	-1.07	0.2	-0.15	-0.82	-0.27	-0.226	-2.47	17.08
4Ca3	0.56	-0.95	-0.4	-0.55	-1.12	-0.49	-1.065	-4.05	17.1
4Ca4	0.14	-1.16	-0.25	-0.55	-0.95	-0.55	-1	-4.2	17.1
4Cb1	0.12	-1.23	-0.34	-0.54	-1.1	-0.62	-0.696	-2.64	17.16
4Cb2	0.04	-3.98	-1.26	-3.57	-1.76	-2.1	-3.876	-3.75	23.67
4Cb3	0.12	-4.17	-1.62	-3.57	-0.83	-2.01	-1.198	-3.35	24.32
4Cb4	0.14	-3.63	-1.67	-1.35	-2.05	-1.71	-1.051	-3.11	25.02
4Cb5	0.62	-0.87	0.05	-0.16	-0.85	-0.24	-0.338	-2.25	16.36
4Cb6	0.15	-4.17	-0.87	-3.19	-1.53	-1.92	-3.518	-4.79	22.84
4Cb7	0.61	-3.74	-1.62	-3.57	-1.65	-1.99	-0.503	-3.39	24.32
4Da1	0	-3.93	0.36	-3.36	-1.39	-1.66	-4.31	-3.46	17.96
4Da2	0	-2.7	0.55	-3.27	-1.47	-1.38	-1.04	-3.88	17.93
4Da3	0	-2.66	0.41	-3.27	-1.47	-1.4	-1.07	-3.92	17.98
4Da4	-0.27	-3.3	0.56	-3.27	-1.23	-1.5	-3.076	-6.56	19.34
4Da5	-0.78	-2.4	0.56	-3.27	-1.4	-1.46	-3.137	-6.47	19.3
4Da6	-0.24	-2.4	0.42	-3.27	-1.4	-1.38	-3.137	-6.51	19.34
4Da7	0.25	-2.13	0.08	-3.66	-0.79	-1.25	-2.727	-5.02	18.59
4Da8	0.5	-1.79	-0.13	-1.45	-1.08	-0.79	-0.601	-4.42	19.22
4Da9	0.37	-2.07	-0.08	-3.66	0.24	-1.04	-0.73	-4.59	18.55
4Da10	0.32	-2.33	0.47	-3.29	-0.57	-1.08	-2.798	-4.85	17.77
4Da11	0.26	-1.9	-0.08	-3.66	-0.68	-1.21	-0.482	-4.5	18.59
4Db1	-0.09	-4.01	-1.11	-3.57	1.33	-1.49	-1.566	-7.05	18.59
4Db2	0.01	-4.13	-0.56	-3.57	-1.47	-1.94	-3.212	-7.2	18.59

ID code	iLOGP	XLOGP	WLOGP	MLOGP	Silicos-IT Log P	Consensus Log P	logP	logD at pH 7,4	HLB
4Db3	0.03	-3.9	-0.72	-3.57	-1.36	-.1.9	-1.396	-6.26	18.59
4Db4	0.42	-4.32	-0.17	-3.19	-1.25	-1.7	-3.712	-7.23	17.77
4Db5	-0.17	-3.99	-0.68	-3.19	-1.15	-1.83	-0.343	-6.82	18.35
4Db6	0.37	-3.64	-0.29	-2.84	-0.92	-1.46	-0.843	-6.86	17.57
4Dc1	-0.93	-5.41	-0.2	-1.76	-2.63	-2.19	-2.867	-7.02	17.57
4Dc2	-0.88	-5.87	-0.55	-1.76	-2.63	-.2.34	-2.867	-6.94	18.03
4Dc3	-1.16	-5.87	-0.55	-1.76	-2.63	-2.39	-2.867	-7.31	18.03
4Dc4	-0.02	-5.15	0.46	-0.96	-1.66	-1.47	-1.593	-5.89	18.41
4Dc5	0.15	-5.6	0.11	-0.96	-1.66	-1.59	-1.593	-6.17	18.94
4Dc6	-0.84	-8.9	-2.23	-5.99	-4.48	-4.49	-7.065	-11.01	25.73
4Dc7	-1.42	-8.66	-2.39	-5.58	-4.42	-4.5	-4.488	-10.22	25.73
4Dc8	-1.23	-7.93	-1.46	-2.54	-3.37	-3.31	-3.615	-6.1	23.71
4Dd1.1	0.69	-4.39	-0.84	-3.07	-1.51	-1.82	-2.971	-4.01	23.23
4Dd1.2	0.48	-3.52	-1.23	-3.46	-1.51	-1.85	-3.62	-4.59	23.26
4Dd1.3	0.63	-2.81	-1.09	-3.46	-1.55	-1.66	-2.599	-4.27	23.21
4Dd1.4	0.68	-3.74	-1.74	-3.46	-1.38	-1.93	-1.109	-4.35	24.03
4Dd2.1	1.04	-3.18	-0.45	-3.98	-1.82	-1.68	-3.506	-3.83	23.47
4Dd3.1	0.52	-2.76	-0.81	-1.23	-0.59	-0.97	-0.657	-3.92	17.88
4Dd4.1	0.87	-4.87	-3.09	-1.6	-1.04	-1.95	-4.246	-5.99	28.59
4Dd4.2	1.21	-4.52	-2.7	-1.33	-0.65	-1.59	-3.888	-5.93	28.02
4Dd4.3	0.18	-4.77	-2.6	-1.37	-0.89	-1.89	-4.099	-8.87	28.52
4Dd4.m	0.55	-4	-1.95	-2.95	-0.52	-1.77	-3.272	-4.75	22.51
5Aa1	2.25	-1.43	2.32	-1.76	3.32	0.94	1.072	-4.83	16.05
5Aa2	3.57	1.32	2.57	1.2	4.18	2.57	1.395	1.43	13.09
5Aa3	3.08	-1.3	2.12	-1.94	2.13	0.82	-1.417	-3.19	20.58
5Aa4	3.04	-1.3	2.12	0.28	2.13	1.25	0.749	-3.39	20.58

ID code	iLOGP	XLOGP	WLOGP	MLOGP	Silicos-IT Log P	Consensus Log P	logP	logD at pH 7,4	HLB
5Ba1	4.63	3.51	4.74	0.51	6.37	3.95	-0.004	0.8	22.89
5Ba2	4.81	3.51	4.74	2.73	6.37	4.43	2.162	1.28	22.89
5Ca1	2.8	0.97	2.72	-0.45	3.28	1.86	-0.179	-0.27	14.42
5Ca2	2.65	0.97	2.72	1.76	3.28	2.28	1.987	-0.5	14.42
5Da1	2.12	-1.98	0.04	-1.62	0.36	-0.21	-2.708	-4.63	21.64
5Da2	2.45	-1.98	0.04	-1.62	0.36	-0.15	-2.708	-4.42	21.64
5Da3	2.07	-4.71	-0.22	-4.35	0.05	-1.43	-3.071	-7.7	28.17
5Ea1	-1.98	-3.19	-0.26	-1.78	-0.1	-1.46	-3.411	-4.81	20.12
5Ea2	-1.37	-3.19	-0.26	-1.78	-0.1	-1.34	-3.411	-4.81	20.12
5Ea3	-1.69	-5.92	-0.52	-4.5	-0.42	-2.61	-3.774	-7.65	30.7

Table S6. Toxicity (I): Cramer rules, Kroess and Verhaar scheme.

ID code	Cramer rules	Kroess TTC		Verhaar scheme
	Toxic hazard	Estimate	Explanation	
1Aa1	Low (Class I)	Substance would not be expected to be a safety concern	Verify structural alerts for potential genotoxic carcinogenicity	Class 5 (Not possible to classify according to these rules)
1Aa2	Low (Class I)	Substance would not be expected to be a safety concern	Verify structural alerts for potential genotoxic carcinogenicity	Class 5 (Not possible to classify according to these rules)
1Aa3	Low (Class I)	Substance would not be expected to be a safety concern	Verify structural alerts for potential genotoxic carcinogenicity	Class 5 (Not possible to classify according to these rules)
1Aa4	Low (Class I)	Substance would not be expected to be a safety concern	Verify structural alerts for potential genotoxic carcinogenicity	Class 5 (Not possible to classify according to these rules)
1Aa5	Low (Class I)	Substance would not be expected to be a safety concern	Verify structural alerts for potential genotoxic carcinogenicity	Class 5 (Not possible to classify according to these rules)
1Aa6	Low (Class I)	Substance would not be expected to be a safety concern	Verify structural alerts for potential genotoxic carcinogenicity	Class 5 (Not possible to classify according to these rules)
1Aa7	Low (Class I)	Substance would not be expected to be a safety concern	Verify structural alerts for potential genotoxic carcinogenicity	Class 5 (Not possible to classify according to these rules)
1Aa8	Low (Class I)	Substance would not be expected to be a safety concern	Verify structural alerts for potential genotoxic carcinogenicity	Class 5 (Not possible to classify according to these rules)
1Ba1	Intermediate (Class II)	Substance would not be expected to be a safety concern	Verify structural alerts for potential genotoxic carcinogenicity	Class 5 (Not possible to classify according to these rules)
1Ba2	High (Class III)	Substance would not be expected to be a safety concern	Verify structural alerts for potential genotoxic carcinogenicity	Class 5 (Not possible to classify according to these rules)
1Ba3	High (Class III)	Substance would not be expected to be a safety concern	Verify structural alerts for potential genotoxic carcinogenicity	Class 5 (Not possible to classify according to these rules)
1Ca1	High (Class III)	Negligible risk (low probability of a life-time cancer risk greater than 1 in 10^6)	Genotoxic potential. Acyl halides	Class 5 (Not possible to classify according to these rules)
1Ca2	High (Class III)	Negligible risk (low probability of a life-time cancer risk greater than 1 in 10^6)	Genotoxic potential. Acyl halides	Class 3 (unspecific reactivity)
1Ca3	High (Class III)	Negligible risk (low probability of a life-time cancer risk greater than 1 in 10^6)	Genotoxic potential. Acyl halides	Class 3 (unspecific reactivity)
1Da1	High (Class III)	Substance would not be expected to be a safety concern	Verify structural alerts for potential genotoxic carcinogenicity	Class 3 (unspecific reactivity)
1Da2	High (Class III)	Substance would not be expected to be a safety concern	Verify structural alerts for potential genotoxic carcinogenicity	Class 5 (Not possible to classify according to these rules)
1Da3	High (Class III)	Substance would not be expected to be a safety concern	Verify structural alerts for potential genotoxic carcinogenicity	Class 3 (unspecific reactivity)

ID code	Cramer rules	Kroess TTC		Verhaar scheme
	Toxic hazard	Estimate	Explanation	
1Da4	High (Class III)	Substance would not be expected to be a safety concern	Verify structural alerts for potential genotoxic carcinogenicity	Class 3 (unspecific reactivity)
2Aa1	High (Class III)	Substance would not be expected to be a safety concern	Verify structural alerts for potential genotoxic carcinogenicity	Class 5 (Not possible to classify according to these rules)
2Ba1	High (Class III)	Substance would not be expected to be a safety concern	Verify structural alerts for potential genotoxic carcinogenicity	Class 5 (Not possible to classify according to these rules)
2Ba2	Low (Class I)	Substance would not be expected to be a safety concern	Verify structural alerts for potential genotoxic carcinogenicity	Class 5 (Not possible to classify according to these rules)
2Ba3	High (Class III)	Substance would not be expected to be a safety concern	Verify structural alerts for potential genotoxic carcinogenicity	Class 5 (Not possible to classify according to these rules)
2Ba4	High (Class III)	Substance would not be expected to be a safety concern	Verify structural alerts for potential genotoxic carcinogenicity	Class 5 (Not possible to classify according to these rules)
2Ba5	Low (Class I)	Substance would not be expected to be a safety concern	Verify structural alerts for potential genotoxic carcinogenicity	Class 5 (Not possible to classify according to these rules)
2Ba6	Low (Class I)	Substance would not be expected to be a safety concern	Verify structural alerts for potential genotoxic carcinogenicity	Class 5 (Not possible to classify according to these rules)
2Ba6a	High (Class III)	Negligible risk (low probability of a life-time cancer risk greater than 1 in 10^6)	Genotoxic potential. Gen.S or N mustard	Class 3 (unspecific reactivity)
2Ca1	Intermediate (Class II)	Substance would not be expected to be a safety concern	Verify structural alerts for potential genotoxic carcinogenicity	Class 5 (Not possible to classify according to these rules)
3Aa1	High (Class III)	Substance would not be expected to be a safety concern	Verify structural alerts for potential genotoxic carcinogenicity	Class 5 (Not possible to classify according to these rules)
3Aa2	High (Class III)	Substance would not be expected to be a safety concern	Verify structural alerts for potential genotoxic carcinogenicity	Class 5 (Not possible to classify according to these rules)
3Aa3	High (Class III)	Substance would not be expected to be a safety concern	Verify structural alerts for potential genotoxic carcinogenicity	Class 5 (Not possible to classify according to these rules)
3Aa4	High (Class III)	Substance would not be expected to be a safety concern	Verify structural alerts for potential genotoxic carcinogenicity	Class 5 (Not possible to classify according to these rules)
3Aa5	High (Class III)	Substance would not be expected to be a safety concern	Verify structural alerts for potential genotoxic carcinogenicity	Class 5 (Not possible to classify according to these rules)
3Aa6	High (Class III)	Substance would not be expected to be a safety concern	Verify structural alerts for potential genotoxic carcinogenicity	Class 5 (Not possible to classify according to these rules)
3Aa7	High (Class III)	Substance would not be expected to be a safety concern	Verify structural alerts for potential genotoxic carcinogenicity	Class 5 (Not possible to classify according to these rules)
3Aa8	High (Class III)	Substance would not be expected to be a safety concern	Verify structural alerts for potential genotoxic carcinogenicity	Class 5 (Not possible to classify according to these rules)

ID code	Cramer rules	Kroess TTC		Verhaar scheme
	Toxic hazard	Estimate	Explanation	
3Aa9	High (Class III)	Substance would not be expected to be a safety concern	Verify structural alerts for potential genotoxic carcinogenicity	Class 3 (unspecific reactivity)
3Ab10	High (Class III)	Substance would not be expected to be a safety concern	Verify structural alerts for potential genotoxic carcinogenicity	Class 3 (unspecific reactivity)
3Ba1	High (Class III)	Substance would not be expected to be a safety concern	Verify structural alerts for potential genotoxic carcinogenicity	Class 5 (Not possible to classify according to these rules)
3Ba2	High (Class III)	Substance would not be expected to be a safety concern	Verify structural alerts for potential genotoxic carcinogenicity	Class 5 (Not possible to classify according to these rules)
3Ba3	High (Class III)	Substance would not be expected to be a safety concern	Verify structural alerts for potential genotoxic carcinogenicity	Class 5 (Not possible to classify according to these rules)
3Ba4	High (Class III)	Substance would not be expected to be a safety concern	Verify structural alerts for potential genotoxic carcinogenicity	Class 5 (Not possible to classify according to these rules)
3Ba5	High (Class III)	Substance would not be expected to be a safety concern	Verify structural alerts for potential genotoxic carcinogenicity	Class 5 (Not possible to classify according to these rules)
3Ba6	High (Class III)	Substance would not be expected to be a safety concern	Verify structural alerts for potential genotoxic carcinogenicity	Class 5 (Not possible to classify according to these rules)
3Bb7	High (Class III)	Substance would not be expected to be a safety concern	Verify structural alerts for potential genotoxic carcinogenicity	Class 3 (unspecific reactivity)
3Bb8	High (Class III)	Substance would not be expected to be a safety concern	Verify structural alerts for potential genotoxic carcinogenicity	Class 3 (unspecific reactivity)
4Aa1	High (Class III)	Negligible risk (low probability of a life-time cancer risk greater than 1 in 10^6)	Genotoxic potential. Gen.S or N mustard	Class 3 (unspecific reactivity)
4Aa2	High (Class III)	Negligible risk (low probability of a life-time cancer risk greater than 1 in 10^6)	Genotoxic potential. Gen.S or N mustard	Class 3 (unspecific reactivity)
4Aa3	High (Class III)	Negligible risk (low probability of a life-time cancer risk greater than 1 in 10^6)	Genotoxic potential. Gen.S or N mustard	Class 5 (Not possible to classify according to these rules)
4Ab1	High (Class III)	Risk assessment requires compound-specific toxicity data	Genotoxic potential. Aflatoxin-like, azoxy-, or N-nitroso compound	Class 5 (Not possible to classify according to these rules)
4Ab2	High (Class III)	Risk assessment requires compound-specific toxicity data	Genotoxic potential. Aflatoxin-like, azoxy-, or N-nitroso compound	Class 5 (Not possible to classify according to these rules)
4Ac1	High (Class III)	Negligible risk (low probability of a life-time cancer risk greater than 1 in 10^6)	Genotoxic potential. Hydrazine	Class 3 (unspecific reactivity)
4Ad1	Low (Class I)	Substance would not be expected to be a safety concern	Verify structural alerts for potential genotoxic carcinogenicity	Class 5 (Not possible to classify according to these rules)
4Ad2	Low (Class I)	Substance would not be expected to be a safety concern	Verify structural alerts for potential genotoxic carcinogenicity	Class 5 (Not possible to classify according to these rules)

ID code	Cramer rules	Kroess TTC		Verhaar scheme
	Toxic hazard	Estimate	Explanation	
4Ad3	Low (Class I)	Substance would not be expected to be a safety concern	Verify structural alerts for potential genotoxic carcinogenicity	Class 5 (Not possible to classify according to these rules)
4Ae1	High (Class III)	Substance would not be expected to be a safety concern	Verify structural alerts for potential genotoxic carcinogenicity	Class 5 (Not possible to classify according to these rules)
4Ae2	High (Class III)	Substance would not be expected to be a safety concern	Verify structural alerts for potential genotoxic carcinogenicity	Class 5 (Not possible to classify according to these rules)
4Ba1	Low (Class I)	Substance would not be expected to be a safety concern	Verify structural alerts for potential genotoxic carcinogenicity	Class 5 (Not possible to classify according to these rules)
4Ba2	Low (Class I)	Substance would not be expected to be a safety concern	Verify structural alerts for potential genotoxic carcinogenicity	Class 5 (Not possible to classify according to these rules)
4Ba3	Low (Class I)	Substance would not be expected to be a safety concern	Verify structural alerts for potential genotoxic carcinogenicity	Class 5 (Not possible to classify according to these rules)
4Ba4	Low (Class I)	Substance would not be expected to be a safety concern	Verify structural alerts for potential genotoxic carcinogenicity	Class 5 (Not possible to classify according to these rules)
4Ba5	Low (Class I)	Substance would not be expected to be a safety concern	Verify structural alerts for potential genotoxic carcinogenicity	Class 5 (Not possible to classify according to these rules)
4Ba6	High (Class III)	Substance would not be expected to be a safety concern	Verify structural alerts for potential genotoxic carcinogenicity	Class 3 (unspecific reactivity)
4Ba7	High (Class III)	Substance would not be expected to be a safety concern	Verify structural alerts for potential genotoxic carcinogenicity	Class 5 (Not possible to classify according to these rules)
4Ca1	High (Class III)	Substance would not be expected to be a safety concern	Verify structural alerts for potential genotoxic carcinogenicity	Class 3 (unspecific reactivity)
4Ca2	High (Class III)	Substance would not be expected to be a safety concern	Verify structural alerts for potential genotoxic carcinogenicity	Class 5 (Not possible to classify according to these rules)
4Ca3	High (Class III)	Substance would not be expected to be a safety concern	Verify structural alerts for potential genotoxic carcinogenicity	Class 5 (Not possible to classify according to these rules)
4Ca4	High (Class III)	Substance would not be expected to be a safety concern	Verify structural alerts for potential genotoxic carcinogenicity	Class 5 (Not possible to classify according to these rules)
4Cb1	High (Class III)	Substance would not be expected to be a safety concern	Verify structural alerts for potential genotoxic carcinogenicity	Class 5 (Not possible to classify according to these rules)
4Cb2	High (Class III)	Substance would not be expected to be a safety concern	Verify structural alerts for potential genotoxic carcinogenicity	Class 5 (Not possible to classify according to these rules)
4Cb3	High (Class III)	Negligible risk (low probability of a life-time cancer risk greater than 1 in 10^6)	Genotoxic potential. Epoxides and aziridines	Class 5 (Not possible to classify according to these rules)
4Cb4	High (Class III)	Negligible risk (low probability of a life-time cancer risk greater than 1 in 10^6)	Genotoxic potential. Epoxides and aziridines	Class 5 (Not possible to classify according to these rules)

ID code	Cramer rules	Kroess TTC		Verhaar scheme
	Toxic hazard	Estimate	Explanation	
4Cb5	High (Class III)	Substance would not be expected to be a safety concern	Verify structural alerts for potential genotoxic carcinogenicity	Class 5 (Not possible to classify according to these rules)
4Cb6	High (Class III)	Substance would not be expected to be a safety concern	Verify structural alerts for potential genotoxic carcinogenicity	Class 5 (Not possible to classify according to these rules)
4Cb7	High (Class III)	Substance would not be expected to be a safety concern	Verify structural alerts for potential genotoxic carcinogenicity	Class 5 (Not possible to classify according to these rules)
4Da1	High (Class III)	Substance would not be expected to be a safety concern	Verify structural alerts for potential genotoxic carcinogenicity	Class 5 (Not possible to classify according to these rules)
4Da2	High (Class III)	Substance would not be expected to be a safety concern	Verify structural alerts for potential genotoxic carcinogenicity	Class 5 (Not possible to classify according to these rules)
4Da3	High (Class III)	Substance would not be expected to be a safety concern	Verify structural alerts for potential genotoxic carcinogenicity	Class 5 (Not possible to classify according to these rules)
4Da4	High (Class III)	Substance would not be expected to be a safety concern	Verify structural alerts for potential genotoxic carcinogenicity	Class 5 (Not possible to classify according to these rules)
4Da5	High (Class III)	Substance would not be expected to be a safety concern	Verify structural alerts for potential genotoxic carcinogenicity	Class 5 (Not possible to classify according to these rules)
4Da6	High (Class III)	Substance would not be expected to be a safety concern	Verify structural alerts for potential genotoxic carcinogenicity	Class 5 (Not possible to classify according to these rules)
4Da7	High (Class III)	Substance would not be expected to be a safety concern	Verify structural alerts for potential genotoxic carcinogenicity	Class 5 (Not possible to classify according to these rules)
4Da8	High (Class III)	Negligible risk (low probability of a life-time cancer risk greater than 1 in 10^6)	Genotoxic potential. Epoxides and aziridines	Class 3 (unspecific reactivity)
4Da9	High (Class III)	Negligible risk (low probability of a life-time cancer risk greater than 1 in 10^6)	Genotoxic potential. Epoxides and aziridines	Class 3 (unspecific reactivity)
4Da10	High (Class III)	Substance would not be expected to be a safety concern	Verify structural alerts for potential genotoxic carcinogenicity	Class 5 (Not possible to classify according to these rules)
4Da11	High (Class III)	Substance would not be expected to be a safety concern	Verify structural alerts for potential genotoxic carcinogenicity	Class 5 (Not possible to classify according to these rules)
4Db1	High (Class III)	Risk assessment requires compound-specific toxicity data	Non-essential metal or metal-containing compound, or is it a polyhalogenated dibenzodioxin, -dibenzofuran, or -biphenyl	Class 5 (Not possible to classify according to these rules)
4Db2	High (Class III)	Risk assessment requires compound-specific toxicity data	Non-essential metal or metal-containing compound, or is it a polyhalogenated dibenzodioxin, -dibenzofuran, or -biphenyl	Class 5 (Not possible to classify according to these rules)
4Db3	High (Class III)	Risk assessment requires compound-specific toxicity data	Non-essential metal or metal-containing compound, or is it a polyhalogenated	Class 5 (Not possible to classify according to these rules)

ID code	Cramer rules	Kroess TTC		Verhaar scheme
	Toxic hazard	Estimate	Explanation	
			dibenzodioxin, -dibenzofuran, or -biphenyl	
4Db4	High (Class III)	Risk assessment requires compound-specific toxicity data	Non-essential metal or metal-containing compound, or is it a polyhalogenated dibenzodioxin, -dibenzofuran, or -biphenyl	Class 5 (Not possible to classify according to these rules)
4Db5	High (Class III)	Risk assessment requires compound-specific toxicity data	Non-essential metal or metal-containing compound, or is it a polyhalogenated dibenzodioxin, -dibenzofuran, or -biphenyl	Class 5 (Not possible to classify according to these rules)
4Db6	High (Class III)	Risk assessment requires compound-specific toxicity data	Non-essential metal or metal-containing compound, or is it a polyhalogenated dibenzodioxin, -dibenzofuran, or -biphenyl	Class 5 (Not possible to classify according to these rules)
4Dc1	High (Class III)	Risk assessment requires compound-specific toxicity data	Non-essential metal or metal-containing compound, or is it a polyhalogenated dibenzodioxin, -dibenzofuran, or -biphenyl	Class 5 (Not possible to classify according to these rules)
4Dc2	High (Class III)	Risk assessment requires compound-specific toxicity data	Non-essential metal or metal-containing compound, or is it a polyhalogenated dibenzodioxin, -dibenzofuran, or -biphenyl	Class 5 (Not possible to classify according to these rules)
4Dc3	High (Class III)	Risk assessment requires compound-specific toxicity data	Non-essential metal or metal-containing compound, or is it a polyhalogenated dibenzodioxin, -dibenzofuran, or -biphenyl	Class 5 (Not possible to classify according to these rules)
4Dc4	High (Class III)	Risk assessment requires compound-specific toxicity data	Non-essential metal or metal-containing compound, or is it a polyhalogenated dibenzodioxin, -dibenzofuran, or -biphenyl	Class 5 (Not possible to classify according to these rules)
4Dc5	High (Class III)	Risk assessment requires compound-specific toxicity data	Non-essential metal or metal-containing compound, or is it a polyhalogenated dibenzodioxin, -dibenzofuran, or -biphenyl	Class 5 (Not possible to classify according to these rules)
4Dc6	High (Class III)	Risk assessment requires compound-specific toxicity data	Non-essential metal or metal-containing compound, or is it a polyhalogenated dibenzodioxin, -dibenzofuran, or -biphenyl	Class 5 (Not possible to classify according to these rules)
4Dc7	High (Class III)	Risk assessment requires compound-specific toxicity data	Non-essential metal or metal-containing compound, or is it a polyhalogenated dibenzodioxin, -dibenzofuran, or -biphenyl	Class 5 (Not possible to classify according to these rules)
4Dc8	High (Class III)	Risk assessment requires compound-specific toxicity data	Non-essential metal or metal-containing compound, or is it a polyhalogenated dibenzodioxin, -dibenzofuran, or -biphenyl	Class 5 (Not possible to classify according to these rules)
4Dd1.1	High (Class III)	Substance would not be expected to be a safety concern	Verify structural alerts for potential genotoxic carcinogenicity	Class 5 (Not possible to classify according to these rules)

ID code	Cramer rules	Kroess TTC		Verhaar scheme
	Toxic hazard	Estimate	Explanation	
4Dd1.2	High (Class III)	Substance would not be expected to be a safety concern	Verify structural alerts for potential genotoxic carcinogenicity	Class 5 (Not possible to classify according to these rules)
4Dd1.3	High (Class III)	Substance would not be expected to be a safety concern	Verify structural alerts for potential genotoxic carcinogenicity	Class 5 (Not possible to classify according to these rules)
4Dd1.4	High (Class III)	Substance would not be expected to be a safety concern	Verify structural alerts for potential genotoxic carcinogenicity	Class 5 (Not possible to classify according to these rules)
4Dd2.1	High (Class III)	Risk assessment requires compound-specific toxicity data	Genotoxic potential. Aflatoxin-like, azoxy-, or N-nitroso compound. Alkyl and aryl N-nitroso groups	Class 5 (Not possible to classify according to these rules)
4Dd3.1	High (Class III)	Substance would not be expected to be a safety concern	Verify structural alerts for potential genotoxic carcinogenicity	Class 5 (Not possible to classify according to these rules)
4Dd4.1	High (Class III)	Substance would not be expected to be a safety concern	Verify structural alerts for potential genotoxic carcinogenicity	Class 5 (Not possible to classify according to these rules)
4Dd4.2	High (Class III)	Substance would not be expected to be a safety concern	Verify structural alerts for potential genotoxic carcinogenicity	Class 5 (Not possible to classify according to these rules)
4Dd4.3	High (Class III)	Substance would not be expected to be a safety concern	Verify structural alerts for potential genotoxic carcinogenicity	Class 5 (Not possible to classify according to these rules)
4Dd4.m	High (Class III)	Substance would not be expected to be a safety concern	Verify structural alerts for potential genotoxic carcinogenicity	Class 5 (Not possible to classify according to these rules)
5Aa1	Low (Class I)	Substance would not be expected to be a safety concern	Verify structural alerts for potential genotoxic carcinogenicity	Class 5 (Not possible to classify according to these rules)
5Aa2	High (Class III)	Substance would not be expected to be a safety concern	Verify structural alerts for potential genotoxic carcinogenicity	Class 5 (Not possible to classify according to these rules)
5Aa3	Low (Class I)	Substance would not be expected to be a safety concern	Verify structural alerts for potential genotoxic carcinogenicity	Class 5 (Not possible to classify according to these rules)
5Aa4	Low (Class I)	Substance would not be expected to be a safety concern	Verify structural alerts for potential genotoxic carcinogenicity	Class 5 (Not possible to classify according to these rules)
5Ba1	High (Class III)	Substance would not be expected to be a safety concern	Verify structural alerts for potential genotoxic carcinogenicity	Class 5 (Not possible to classify according to these rules)
5Ba2	High (Class III)	Substance would not be expected to be a safety concern	Verify structural alerts for potential genotoxic carcinogenicity	Class 5 (Not possible to classify according to these rules)
5Ca1	High (Class III)	Negligible risk (low probability of a life-time cancer risk greater than 1 in 10^6)	Genotoxic potential. Polycyclic Aromatic Hydrocarbons	Class 5 (Not possible to classify according to these rules)
5Ca2	High (Class III)	Negligible risk (low probability of a life-time cancer risk greater than 1 in 10^6)	Genotoxic potential. Polycyclic Aromatic Hydrocarbons	Class 5 (Not possible to classify according to these rules)

ID code	Cramer rules	Kroess TTC		Verhaar scheme
	Toxic hazard	Estimate	Explanation	
5Da1	High (Class III)	Substance would not be expected to be a safety concern	Verify structural alerts for potential genotoxic carcinogenicity	Class 5 (Not possible to classify according to these rules)
5Da2	High (Class III)	Substance would not be expected to be a safety concern	Verify structural alerts for potential genotoxic carcinogenicity	Class 5 (Not possible to classify according to these rules)
5Da3	High (Class III)	Substance would not be expected to be a safety concern	Verify structural alerts for potential genotoxic carcinogenicity	Class 5 (Not possible to classify according to these rules)
5Ea1	High (Class III)	Substance would not be expected to be a safety concern	Verify structural alerts for potential genotoxic carcinogenicity	Class 5 (Not possible to classify according to these rules)
5Ea2	High (Class III)	Substance would not be expected to be a safety concern	Verify structural alerts for potential genotoxic carcinogenicity	Class 5 (Not possible to classify according to these rules)
5Ea3	High (Class III)	Substance would not be expected to be a safety concern	Verify structural alerts for potential genotoxic carcinogenicity	Class 5 (Not possible to classify according to these rules)

Table S7. Toxicity (II). Carcinogenic (genotoxic and non-genotoxic) and mutagenic effects evaluated using two different apps (Toxtree and OSIRIS).

ID code	Carcinogenicity			Mutagenicity	
	**Genotoxic Toxtree**	**Non-genotoxic**	**Tumorigenesis OSIRIS**	**Toxtree (Ames test) *S. typhimurium***	**OSIRIS**
1Aa1	Negative	Negative	Negative	Negative	Negative
1Aa2	Negative	Negative	Negative	Negative	Negative
1Aa3	Negative	Negative	Negative	Negative	Negative
1Aa4	Negative	Negative	Negative	Negative	Negative
1Aa5	Negative	Negative	Negative	Negative	Negative
1Aa6	Negative	Negative	Negative	Negative	Negative
1Aa7	Negative	Negative	Negative	Negative	Negative
1Aa8	Negative	Negative	Negative	Negative	Negative
1Ba1	Negative	Negative	Negative	Negative	High risk
1Ba2	Negative	Negative	Negative	Negative	Negative
1Ba3	Negative	Negative	Negative	Negative	Negative
1Ca1	Structural Alert for genotoxic carcinogenicity	Negative	Negative	Structural alert for *S.typhimurium* mutagenicity	High risk
1Ca2	Structural Alert for genotoxic carcinogenicity	Negative	Negative	Structural alert for *S.typhimurium* mutagenicity	High risk
1Ca3	Structural Alert for genotoxic carcinogenicity	Negative	Negative	Structural alert for *S.typhimurium* mutagenicity	High risk
1Da1	Negative	Negative	Negative	Negative	Negative
1Da2	Negative	Negative	Negative	Negative	Negative
1Da3	Negative	Negative	Negative	Negative	Negative
1Da4	Negative	Negative	Negative	Negative	Negative
2Aa1	Negative	Negative	Negative	Negative	Negative
2Ba1	Negative	Negative	Negative	Negative	Negative
2Ba2	Negative	Negative	Negative	Negative	Negative
2Ba3	Negative	Negative	Negative	Negative	Negative

ID code	Carcinogenicity			Mutagenicity	
	Genotoxic Toxtree	Non-genotoxic	Tumorigenesis OSIRIS	Toxtree (Ames test) ***S. typhimurium***	OSIRIS
2Ba4	Negative	Negative	Negative	Negative	Negative
2Ba5	Negative	Negative	Negative	Negative	Negative
2Ba6	Negative	Negative	Negative	Negative	Negative
2Ba6a	Structural Alert for genotoxic carcinogenicity	Negative	High risk	Structural alert for *S.typhimurium* mutagenicity	High risk
2Ca1	Negative	Negative	Negative	Negative	Negative
3Aa1	Negative	Negative	Negative	Negative	Negative
3Aa2	Negative	Negative	Negative	Negative	Negative
3Aa3	Negative	Negative	Negative	Negative	Negative
3Aa4	Negative	Negative	Negative	Negative	Negative
3Aa5	Negative	Negative	Negative	Negative	Negative
3Aa6	Negative	Negative	Negative	Negative	Negative
3Aa7	Negative	Negative	Negative	Negative	Negative
3Aa8	Negative	Negative	Negative	Negative	Negative
3Aa9	Negative	Negative	Negative	Negative	Negative
3Ab10	Negative	Negative	Negative	Negative	Negative
3Ba1	Negative	Negative	Negative	Negative	Negative
3Ba2	Negative	Negative	Negative	Negative	Negative
3Ba3	Negative	Negative	Negative	Negative	Negative
3Ba4	Negative	Negative	Negative	Negative	Negative
3Ba5	Negative	Negative	Negative	Negative	Negative
3Ba6	Negative	Negative	Negative	Negative	Negative
3Bb7	Negative	Negative	Negative	Negative	Negative
3Bb8	Negative	Negative	Negative	Negative	Negative
4Aa1	Structural Alert for genotoxic carcinogenicity	Negative	High risk	Structural alert for *S.typhimurium* mutagenicity	High risk

ID code	Carcinogenicity			Mutagenicity	
	**Genotoxic Toxtree**	**Non-genotoxic**	**Tumorigenesis OSIRIS**	**Toxtree (Ames test) *S. typhimurium***	**OSIRIS**
4Aa2	Structural Alert for genotoxic carcinogenicity	Negative	High risk	Structural alert for *S.typhimurium* mutagenicity	High risk
4Aa3	Structural Alert for genotoxic carcinogenicity	Negative	High risk	Structural alert for *S.typhimurium* mutagenicity	High risk
4Ab1	Structural Alert for genotoxic carcinogenicity	Negative	High risk	Structural alert for *S.typhimurium* mutagenicity	High risk
4Ab2	Structural Alert for genotoxic carcinogenicity	Negative	High risk	Structural alert for *S.typhimurium* mutagenicity	High risk
4Ac1	Structural Alert for genotoxic carcinogenicity	Negative	Medium risk	Structural alert for *S.typhimurium* mutagenicity	High risk
4Ad1	Negative	Negative	High risk	Negative	High risk
4Ad2	Negative	Negative	High risk	Negative	High risk
4Ad3	Negative	Negative	High risk	Negative	High risk
4Ae1	Negative	Negative	Negative	Negative	High risk
4Ae2	Negative	Negative	Negative	Negative	High risk
4Ba1	Negative	Negative	Negative	Negative	Negative
4Ba2	Negative	Negative	Negative	Negative	Negative
4Ba3	Negative	Negative	Negative	Negative	Negative
4Ba4	Negative	Negative	Negative	Negative	Negative
4Ba5	Negative	Negative	Negative	Negative	Negative
4Ba6	Negative	Negative	Negative	Negative	Negative
4Ba7	Negative	Negative	Negative	Negative	Negative
4Ca1	Negative	Negative	Negative	Negative	Negative
4Ca2	Negative	Negative	Negative	Negative	Negative
4Ca3	Negative	Negative	Negative	Structural alert for *S.typhimurium* mutagenicity	Negative
4Ca4	Negative	Negative	Negative	Structural alert for *S.typhimurium* mutagenicity	Negative
4Cb1	Negative	Negative	Negative	Negative	Negative

ID code	Carcinogenicity			Mutagenicity	
	**Genotoxic Toxtree**	**Non-genotoxic**	**Tumorigenesis OSIRIS**	**Toxtree (Ames test) *S. typhimurium***	**OSIRIS**
4Cb2	Negative	Negative	Negative	Negative	Negative
4Cb3	Structural Alert for genotoxic carcinogenicity	Negative	Medium risk	Structural alert for *S.typhimurium* mutagenicity	Medium risk
4Cb4	Structural Alert for genotoxic carcinogenicity	Negative	Medium risk	Structural alert for *S.typhimurium* mutagenicity	High risk
4Cb5	Negative	Negative	Negative	Negative	Negative
4Cb6	Negative	Negative	Negative	Negative	Negative
4Cb7	Negative	Negative	Negative	Negative	Negative
4Da1	Negative	Negative	Negative	Negative	Negative
4Da2	Negative	Negative	Negative	Negative	Negative
4Da3	Negative	Negative	Negative	Negative	Negative
4Da4	Negative	Negative	Negative	Negative	Negative
4Da5	Negative	Negative	Negative	Negative	Negative
4Da6	Negative	Negative	Negative	Negative	Negative
4Da7	Negative	Negative	Negative	Negative	Negative
4Da8	Structural Alert for genotoxic carcinogenicity	Negative	Medium risk	Structural alert for *S.typhimurium* mutagenicity	Medium risk
4Da9	Structural Alert for genotoxic carcinogenicity	Negative	Medium risk	Structural alert for *S.typhimurium* mutagenicity	Medium risk
4Da10	Negative	Negative	Negative	Negative	Negative
4Da11	Negative	Negative	Negative	Negative	Negative
4Db1	Structural Alert for genotoxic carcinogenicity	Negative	Medium risk	Structural alert for *S.typhimurium* mutagenicity	Medium risk
4Db2	Negative	Negative	Negative	Negative	Negative
4Db3	Negative	Negative	Negative	Negative	Negative
4Db4	Negative	Negative	Negative	Negative	Negative
4Db5	Negative	Negative	Negative	Negative	Negative
4Db6	Negative	Negative	Negative	Negative	Negative

ID code	Carcinogenicity			Mutagenicity	
	**Genotoxic Toxtree**	**Non-genotoxic**	**Tumorigenesis OSIRIS**	**Toxtree (Ames test) *S. typhimurium***	**OSIRIS**
4Dc1	Negative	Negative	Negative	Negative	Negative
4Dc2	Negative	Negative	Negative	Negative	Negative
4Dc3	Negative	Negative	Negative	Negative	Negative
4Dc4	Negative	Negative	Negative	Negative	Negative
4Dc5	Negative	Negative	Negative	Negative	Negative
4Dc6	Negative	Negative	Negative	Negative	Negative
4Dc7	Negative	Negative	Negative	Negative	Negative
4Dc8	Negative	Negative	Negative	Negative	Negative
4Dd1.1	Negative	Negative	Negative	Negative	Negative
4Dd1.2	Negative	Negative	Negative	Negative	Negative
4Dd1.3	Negative	Negative	Negative	Negative	Negative
4Dd1.4	Negative	Negative	Negative	Negative	Negative
4Dd2.1	Structural Alert for genotoxic carcinogenicity	Negative	High risk	Structural alert for *S.typhimurium* mutagenicity	High risk
4Dd3.1	Negative	Negative	Negative	Negative	Negative
4Dd4.1	Negative	Negative	Negative	Negative	Negative
4Dd4.2	Negative	Negative	Negative	Negative	Negative
4Dd4.3	Negative	Negative	Negative	Negative	Negative
4Dd4.m	Negative	Negative	Negative	Negative	Negative
5Aa1	Negative	Negative	Negative	Negative	Negative
5Aa2	Negative	Negative	Negative	Negative	Negative
5Aa3	Negative	Negative	Negative	Negative	Negative
5Aa4	Negative	Negative	Negative	Negative	Negative
5Ba1	Negative	Negative	Negative	Negative	Negative
5Ba2	Negative	Negative	Negative	Negative	Negative

ID code	Carcinogenicity			Mutagenicity	
	**Genotoxic Toxtree**	**Non-genotoxic**	**Tumorigenesis OSIRIS**	**Toxtree (Ames test) *S. typhimurium***	**OSIRIS**
5Ca1	Structural Alert for genotoxic carcinogenicity	Structural Alert for nongenotoxic carcinogenicity	High risk	Structural alert for *S. typhimurium* mutagenicity	High risk
5Ca2	Structural Alert for genotoxic carcinogenicity	Structural Alert for nongenotoxic carcinogenicity	High risk	Structural alert for *S. typhimurium* mutagenicity	High risk
5Da1	Negative	Structural Alert for nongenotoxic carcinogenicity	Negative	Negative	Negative
5Da2	Negative	Structural Alert for nongenotoxic carcinogenicity	Negative	Negative	Negative
5Da3	Negative	Structural Alert for nongenotoxic carcinogenicity	Negative	Negative	Negative
5Ea1	Negative	Structural Alert for nongenotoxic carcinogenicity	Negative	Negative	Negative
5Ea2	Negative	Structural Alert for nongenotoxic carcinogenicity	Negative	Negative	Negative
5Ea3	Negative	Structural Alert for nongenotoxic carcinogenicity	Negative	Negative	Negative

Table S8. Toxicity (III). Irritant/corrosive effect on the skin and eyes, effect on the reproductive system, biodegradability, protein and DNA binding alerts, assessed using Toxtree and OSIRIS.

ID code	Irritation/corrosion			Effect on the reproductive system	Biodegradability	Protein binding Alerts	DNA binding Alerts
	Skin	Eye	OSIRIS	OSIRIS			
1Aa1	Not irritating or corrosive	Negative	Negative	Negative	Class1 (easily biodegradable chemical)	Negative	Negative
1Aa2	Unknown	Unknown	Negative	Negative	Class1 (easily biodegradable chemical)	Negative	Negative
1Aa3	Unknown	Unknown	Negative	Negative	Class1 (easily biodegradable chemical)	Negative	Negative
1Aa4	Unknown	Unknown	Negative	Negative	Class1 (easily biodegradable chemical)	Negative	Negative
1Aa5	Unknown	Unknown	Negative	Negative	Class1 (easily biodegradable chemical)	Negative	Negative
1Aa6	Unknown	Unknown	Negative	Negative	Class1 (easily biodegradable chemical)	Negative	Negative
1Aa7	Unknown	Unknown	Negative	Negative	Class1 (easily biodegradable chemical)	Negative	Negative
1Aa8	Unknown	Unknown	Negative	Negative	Class1 (easily biodegradable chemical)	Negative	Negative
1Ba1	Not irritating or corrosive	No skin corrosion	Negative	Negative	Class1 (easily biodegradable chemical)	Negative	Negative
1Ba2	Unknown	Unknown	Negative	Negative	Class1 (easily biodegradable chemical)	Negative	Negative
1Ba3	Unknown	Unknown	Negative	Negative	Class1 (easily biodegradable	Negative	Negative

ID code	Irritation/corrosion			Effect on the reproductive system	Biodegradability	Protein binding Alerts	DNA binding Alerts
	Skin	Eye	OSIRIS	OSIRIS			
					chemical)		
1Ca1	Unknown	Unknown	Medium risk	Negative	Class1 (easily biodegradable chemical)	Alert for acyl transfer agent identified	Alert for acyl transfer agent identified
1Ca2	Unknown	Unknown	Medium risk	Negative	Class1 (easily biodegradable chemical)	Alert for acyl transfer agent identified	Alert for acyl transfer agent identified
1Ca3	Unknown	Unknown	Medium risk	Negative	Class1 (easily biodegradable chemical)	Alert for acyl transfer agent identified	Alert for acyl transfer agent identified
1Da1	Unknown	Unknown	High risk	Negative	Class1 (easily biodegradable chemical)	Alert for acyl transfer agent identified	Negative
1Da2	Unknown	Unknown	High risk	Negative	Class1 (easily biodegradable chemical)	Alert for acyl transfer agent identified	Negative
1Da3	Unknown	Unknown	High risk	Negative	Class1 (easily biodegradable chemical)	Alert for acyl transfer agent identified	Negative
1Da4	Unknown	Unknown	High risk	Negative	Class1 (easily biodegradable chemical)	Alert for acyl transfer agent identified	Negative
2Aa1	Unknown	Unknown	Negative	Negative	Class1 (easily biodegradable chemical)	Negative	Negative
2Ba1	Unknown	Unknown	Negative	Negative	Class1 (easily biodegradable chemical)	Negative	Negative
2Ba2	Unknown	Unknown	Negative	Negative	Class2 (persistent chemical)	Negative	Alert for SN1 identified
2Ba3	Unknown	Unknown	Negative	Negative	Class1 (easily biodegradable chemical)	Negative	Negative
2Ba4	Unknown	Unknown	Negative	Negative	Class1 (easily	Negative	Negative

ID code	Irritation/corrosion			Effect on the reproductive system	Biodegradability	Protein binding Alerts	DNA binding Alerts
	Skin	Eye	OSIRIS	OSIRIS			
					biodegradable chemical)		
2Ba5	Unknown	Unknown	Negative	Negative	Class2 (persistent chemical)	Negative	Alert for SN1 identified
2Ba6	Unknown	Unknown	Negative	Negative	Class2 (persistent chemical)	Negative	Alert for SN1 identified
2Ba6a	Unknown	Unknown	Negative	High risk	Class2 (persistent chemical)	Alert for SN2 identified	Alert for SN1 and SN2 identified
2Ca1	Irritating to skin	Unknown	Negative	Negative	Class1 (easily biodegradable chemical)	Negative	Negative
3Aa1	Not irritating	Unknown	Negative	Negative	Class2 (persistent chemical)	Negative	Alert for SN1 identified. Alert for Schiff base formation identified.
3Aa2	Unknown	Unknown	Negative	Negative	Class1 (easily biodegradable chemical)	Negative	Alert for SN1 identified
3Aa3	Not irritating or corrosive	No skin corrosion	Negative	Negative	Class1 (easily biodegradable chemical)	Negative	Negative
3Aa4	Not irritating	No skin corrosion	Negative	Negative	Class1 (easily biodegradable chemical)	Alert for acyl transfer agent identified	Alert for SN1 identified
3Aa5	Not irritating	Serious lesions to the eye	Negative	Negative	Class1 (easily biodegradable chemical)	Negative	Negative
3Aa6	Not irritating	Unknown	Negative	Negative	Class1 (easily biodegradable chemical)	Alert for acyl transfer agent identified	Alert for SN1 identified
3Aa7	Not irritating	Unknown	Negative	Negative	Class2 (persistent chemical)	Alert for acyl transfer agent identified	Alert for Schiff base formation identified

ID code	Irritation/corrosion			Effect on the reproductive system	Biodegradability	Protein binding Alerts	DNA binding Alerts
	Skin	Eye	OSIRIS	OSIRIS			
3Aa8	Not irritating	Serious lesions to the eye	Negative	Negative	Class2 (persistent chemical)	Alert for SN2 identified Nucleophilic Aliphatic Substitution	Alert for Schiff base formation identified
3Aa9	Unknown	Unknown	High risk	Negative	Class2 (persistent chemical)	Alert for acyl transfer agent identified	Alert for SN1 identified
3Ab10	Not irritating	Unknown	High risk	Negative	Class2 (persistent chemical)	Alert for acyl transfer agent identified	Alert for SN1 identified. Alert for Schiff base formation identified.
3Ba1	Unknown	Unknown	Negative	Negative	Class2 (persistent chemical)	Negative	Alert for SN1 identified
3Ba2	Not irritating or corrosive	No skin corrosion	Negative	Negative	Class1 (easily biodegradable chemical)	Negative	Negative
3Ba3	Unknown	Unknown	Negative	Negative	Class1 (easily biodegradable chemical)	Negative	Negative
3Ba4	Not irritating or corrosive	No skin corrosion	Negative	Negative	Class1 (easily biodegradable chemical)	Negative	Negative
3Ba5	Irritating or corrosive	Unknown	Negative	Negative	Class2 (persistent chemical)	Negative	Alert for SN1 identified
3Ba6	Unknown	Unknown	Negative	Negative	Class2 (persistent chemical)	Alert for acyl transfer agent identified	Negative
3Bb7	Unknown	Unknown	High risk	Negative	Class2 (persistent chemical)	Alert for acyl transfer agent identified	Alert for SN1 identified
3Bb8	Unknown	Unknown	High risk	Negative	Class2 (persistent chemical)	Alert for acyl transfer agent identified	Alert for SN1 identified
4Aa1	Unknown	Unknown	Negative	High risk	Class2 (persistent chemical)	Alert for Michael Acceptor identified. Alert for SN2 identified. Nucleophilic Aliphatic Substitution	Alert for Michael Acceptor identified. Alert for SN2 identified. Nucleophilic Aliphatic

ID code	Irritation/corrosion			Effect on the reproductive system	Biodegradability	Protein binding Alerts	DNA binding Alerts
	Skin	Eye	OSIRIS	OSIRIS			
							Substitution
4Aa2	Unknown	Unknown	Negative	High risk	Class2 (persistent chemical)	Alert for Michael Acceptor identified. Alert for SN2 identified. Nucleophilic Aliphatic Substitution	Alert for Michael Acceptor identified. Alert for SN2 identified. Nucleophilic Aliphatic Substitution
4Aa3	Unknown	Unknown	Negative	High risk	Class2 (persistent chemical)	Alert for SN2 identified.-Nucleophilic Aliphatic Substitution	Alert for SN1 and SN2 identified
4Ab1	Unknown	Unknown	High risk	Medium risk	Class2 (persistent chemical)	Alert for SN2 identified.-Nucleophilic Aliphatic Substitution	Alert for SN2 identified.-Nucleophilic Aliphatic Substitution
4Ab2	Not irritating or corrosive	No skin corrosion	Medium risk	Medium risk	Class2 (persistent chemical)	Negative	Negative
4Ac1	Unknown	Unknown	Negative	Medium risk	Class1 (easily biodegradable chemical)	Negative	Negative
4Ad1	Not irritating	No skin corrosion	Negative	Medium risk	Class3 (Unknown biodegradability)	Alert for SN2 identified.-Nucleophilic Aliphatic Substitution	Alert for SN2 identified.-Nucleophilic Aliphatic Substitution
4Ad2	Not irritating	No skin corrosion	Negative	Negative	Class3 (Unknown biodegradability)	Alert for SN2 identified.-Nucleophilic Aliphatic Substitution	Alert for SN2 identified.-Nucleophilic Aliphatic Substitution
4Ad3	Not irritating	Unknown	Negative	Medium risk	Class3 (Unknown biodegradability)	Alert for SN2 identified.-Nucleophilic Aliphatic Substitution	Alert for SN2 identified.-Nucleophilic Aliphatic

ID code	Irritation/corrosion			Effect on the reproductive system	Biodegradability	Protein binding Alerts	DNA binding Alerts
	Skin	Eye	OSIRIS	OSIRIS			
							Substitution
4Ae1	Unknown	Unknown	Negative	Negative	Class3 (Unknown biodegradability)	Negative	Negative
4Ae2	Unknown	Unknown	Negative	Negative	Class2 (persistent chemical)	Negative	Negative
4Ba1	Not irritating or corrosive	No skin corrosion	Medium risk	Negative	Class1 (easily biodegradable chemical)	Negative	Negative
4Ba2	Unknown	Unknown	Negative	Negative	Class1 (easily biodegradable chemical)	Negative	Negative
4Ba3	Unknown	Unknown	Negative	Negative	Class1 (easily biodegradable chemical)	Negative	Negative
4Ba4	Unknown	Unknown	Medium risk	Negative	Class1 (easily biodegradable chemical)	Negative	Negative
4Ba5	Unknown	Unknown	Negative	Negative	Class1 (easily biodegradable chemical)	Negative	Negative
4Ba6	Unknown	Unknown	Medium risk	Negative	Class1 (easily biodegradable chemical)	Negative	Alert for SN1 identified
4Ba7	Unknown	Unknown	Negative	Negative	Class1 (easily biodegradable chemical)	Alert for SN2 identified.- Nucleophilic Aliphatic Substitution	Alert for SN1 identified
4Ca1	Unknown	Unknown	Negative	Negative	Class1 (easily biodegradable chemical)	Negative	Negative
4Ca2	Unknown	Unknown	Negative	Negative	Class1 (easily biodegradable chemical)	Negative	Negative
4Ca3	Unknown	Unknown	Negative	Negative	Class1 (easily	Negative	Negative

ID code	Irritation/corrosion			Effect on the reproductive system	Biodegradability	Protein binding Alerts	DNA binding Alerts
	Skin	Eye	OSIRIS	OSIRIS			
					biodegradable chemical)		
4Ca4	Unknown	Unknown	Negative	Negative	Class1 (easily biodegradable chemical)	Negative	Negative
4Cb1	Unknown	Unknown	Negative	Negative	Class1 (easily biodegradable chemical)	Negative	Negative
4Cb2	Not irritating or corrosive	No skin corrosion	Negative	Negative	Class1 (easily biodegradable chemical)	Negative	Negative
4Cb3	Unknown	Unknown	Negative	High risk	Class1 (easily biodegradable chemical)	Alert for SN2 identified.-Nucleophilic Aliphatic Substitution	Alert for SN2 identified.-Nucleophilic Aliphatic Substitution
4Cb4	Unknown	Unknown	Negative	High risk	Class1 (easily biodegradable chemical)	Alert for SN2 identified.-Nucleophilic Aliphatic Substitution	Alert for SN1 and SN2 identified
4Cb5	Unknown	Unknown	Negative	Negative	Class1 (easily biodegradable chemical)	Negative	Negative
4Cb6	Not irritating or corrosive	No skin corrosion	Negative	Negative	Class1 (easily biodegradable chemical)	Negative	Negative
4Cb7	Unknown	Unknown	Negative	Negative	Class1 (easily biodegradable chemical)	Negative	Negative
4Da1	Not irritating	No skin corrosion	Negative	Negative	Class1 (easily biodegradable chemical)	Negative	Negative
4Da2	Not irritating	No skin corrosion	Negative	Medium risk	Class1 (easily biodegradable chemical)	Alert for SN2 identified.-Nucleophilic Aliphatic Substitution	Negative

ID code	Irritation/corrosion			Effect on the reproductive system	Biodegradability	Protein binding Alerts	DNA binding Alerts
	Skin	Eye	OSIRIS	OSIRIS			
4Da3	Not irritating	No skin corrosion	Negative	Medium risk	Class1 (easily biodegradable chemical)	Alert for SN2 identified.- Nucleophilic Aliphatic Substitution	Negative
4Da4	Not irritating	No skin corrosion	Negative	Negative	Class1 (easily biodegradable chemical)	Negative	Negative
4Da5	Not irritating or corrosive	No skin corrosion	Negative	Medium risk	Class1 (easily biodegradable chemical)	Negative	Negative
4Da6	Not irritating or corrosive	No skin corrosion	Negative	Medium risk	Class1 (easily biodegradable chemical)	Negative	Negative
4Da7	Not irritating	No skin corrosion	Negative	Medium risk	Class1 (easily biodegradable chemical)	Negative	Negative
4Da8	Not irritating	Unknown	Negative	High risk	Class1 (easily biodegradable chemical)	Alert for SN2 identified.- Nucleophilic Aliphatic Substitution	Alert for SN1 and SN2 identified
4Da9	Not irritating	Unknown	Negative	High risk	Class1 (easily biodegradable chemical)	Alert for SN2 identified.- Nucleophilic Aliphatic Substitution	Alert for SN2 identified.- Nucleophilic Aliphatic Substitution
4Da10	Not irritating	No skin corrosion	Negative	Medium risk	Class1 (easily biodegradable chemical)	Negative	Negative
4Da11	Not irritating	Unknown	Negative	Medium risk	Class1 (easily biodegradable chemical)	Negative	Negative
4Db1	Unknown	Serious lesions to the eye	Negative	High risk	Class1 (easily biodegradable chemical)	Alert for SN2 identified.- Nucleophilic Aliphatic Substitution	Alert for SN2 identified.- Nucleophilic Aliphatic Substitution

ID code	Irritation/corrosion			Effect on the reproductive system	Biodegradability	Protein binding Alerts	DNA binding Alerts
	Skin	Eye	OSIRIS	OSIRIS			
4Db2	Not irritating or corrosive	No skin corrosion	Negative	Medium risk	Class1 (easily biodegradable chemical)	Negative	Negative
4Db3	Unknown	Unknown	Negative	Medium risk	Class1 (easily biodegradable chemical)	Negative	Negative
4Db4	Not irritating or corrosive	No skin corrosion	Negative	Medium risk	Class1 (easily biodegradable chemical)	Negative	Negative
4Db5	Unknown	Unknown	Negative	Medium risk	Class1 (easily biodegradable chemical)	Negative	Negative
4Db6	Unknown	Unknown	Negative	Medium risk	Class1 (easily biodegradable chemical)	Negative	Negative
4Dc1	Unknown	Unknown	Negative	Negative	Class3 (Unknown biodegradability)	Negative	Negative
4Dc2	Unknown	Unknown	Negative	Negative	Class3 (Unknown biodegradability)	Negative	Negative
4Dc3	Unknown	Unknown	Negative	Medium risk	Class3 (Unknown biodegradability)	Negative	Negative
4Dc4	Unknown	Serious lesions to the eye	Negative	Medium risk	Class3 (Unknown biodegradability)	Negative	Negative
4Dc5	Unknown	Serious lesions to the eye	Negative	Negative	Class3 (Unknown biodegradability)	Negative	Negative
4Dc6	Not irritating or corrosive	No skin corrosion	Negative	Negative	Class1 (easily biodegradable chemical)	Negative	Negative
4Dc7	Not irritating or corrosive	No skin corrosion	Negative	Negative	Class1 (easily biodegradable	Negative	Negative

ID code	Irritation/corrosion			Effect on the reproductive system	Biodegradability	Protein binding Alerts	DNA binding Alerts
	Skin	Eye	OSIRIS	OSIRIS			
					chemical)		
4Dc8	Not irritating or corrosive	No skin corrosion	Negative	Negative	Class3 (Unknown biodegradability)	Negative	Negative
4Dd1.1	Unknown	Unknown	Negative	Negative	Class1 (easily biodegradable chemical)	Negative	Negative
4Dd1.2	Not irritating or corrosive	No skin corrosion	Negative	Negative	Class1 (easily biodegradable chemical)	Negative	Negative
4Dd1.3	Unknown	Unknown	Negative	Negative	Class1 (easily biodegradable chemical)	Negative	Negative
4Dd1.4	Unknown	Unknown	Negative	Negative	Class1 (easily biodegradable chemical)	Negative	Negative
4Dd2.1	Not irritating or corrosive	No skin corrosion	High risk	Medium risk	Class2 (persistent chemical)	Negative	Negative
4Dd3.1	Unknown	Unknown	Negative	Negative	Class2 (persistent chemical)	Negative	Negative
4Dd4.1	Not irritating or corrosive	No skin corrosion	Negative	Negative	Class1 (easily biodegradable chemical)	Alert for acyl transfer agent identified	Negative
4Dd4.2	Not irritating or corrosive	No skin corrosion	Negative	Negative	Class1 (easily biodegradable chemical)	Alert for acyl transfer agent identified	Negative
4Dd4.3	Not irritating or corrosive	No skin corrosion	Negative	Negative	Class1 (easily biodegradable chemical)	Alert for acyl transfer agent identified	Negative
4Dd4.m	Not irritating or corrosive	No skin corrosion	Negative	Negative	Class1 (easily biodegradable chemical)	Alert for acyl transfer agent identified	Negative
5Aa1	Not corrosive	No skin corrosion	Negative	High risk	Class2 (persistent chemical)	Alert for Michael Acceptor identified. Alert for SN2 identified.	Alert for Michael Acceptor

ID code	Irritation/corrosion			Effect on the reproductive system	Biodegradability	Protein binding Alerts	DNA binding Alerts
	Skin	Eye	OSIRIS	OSIRIS			
						Nucleophilic Aliphatic Substitution	identified.
5Aa2	Not corrosive	No skin corrosion	Negative	High risk	Class2 (persistent chemical)	Alert for Michael Acceptor identified. Alert for SN2 identified. Nucleophilic Aliphatic Substitution	Alert for SN1 identified. Alert for Michael Acceptor identified.
5Aa3	Not corrosive	No skin corrosion	Negative	High risk	Class2 (persistent chemical)	Alert for Michael Acceptor identified. Alert for SN2 identified. Nucleophilic Aliphatic Substitution	Alert for Michael Acceptor identified
5Aa4	Not corrosive	No skin corrosion	Negative	High risk	Class2 (persistent chemical)	Alert for Michael Acceptor identified. Alert for SN2 identified. Nucleophilic Aliphatic Substitution	Alert for Michael Acceptor identified
5Ba1	Not corrosive	No eye irritation	Negative	Negative	Class2 (persistent chemical)	Alert for Michael Acceptor identified. Alert for SN2 identified. Nucleophilic Aliphatic Substitution. Alert for acyl transfer agent identified	Alert for SN1 identified. Alert for Michael Acceptor identified.
5Ba2	Not corrosive	No eye irritation	Negative	Negative	Class2 (persistent chemical)	Alert for Michael Acceptor identified. Alert for SN2 identified. Nucleophilic Aliphatic Substitution. Alert for acyl transfer agent identified	Alert for SN1 identified. Alert for Michael Acceptor identified.
5Ca1	Not corrosive	No skin corrosion	High risk	High risk	Class2 (persistent chemical)	Alert for Michael Acceptor identified. Alert for SN2 identified. Nucleophilic Aliphatic Substitution. Alert for acyl transfer agent identified	Alert for SN1 identified. Alert for Michael Acceptor identified.
5Ca2	Not corrosive	No skin corrosion	High risk	High risk	Class2 (persistent chemical)	Alert for Michael Acceptor identified. Alert for SN2 identified. Nucleophilic Aliphatic Substitution. Alert for acyl transfer agent identified	Alert for SN1 identified. Alert for Michael Acceptor identified.
5Da1	Not corrosive	No skin corrosion	Negative	Negative	Class2 (persistent chemical)	Alert for Michael Acceptor identified. Alert for SN2 identified.	Alert for SN1 identified. Alert

ID code	Irritation/corrosion			Effect on the reproductive system	Biodegradability	Protein binding Alerts	DNA binding Alerts
	Skin	Eye	OSIRIS	OSIRIS			
						Nucleophilic Aliphatic Substitution	for Michael Acceptor identified.
5Da2	Not corrosive	No skin corrosion	Negative	Negative	Class2 (persistent chemical)	Alert for Michael Acceptor identified. Alert for SN2 identified. Nucleophilic Aliphatic Substitution	Alert for SN1 identified. Alert for Michael Acceptor identified.
5Da3	Not corrosive	No skin corrosion	Negative	Negative	Class2 (persistent chemical)	Alert for Michael Acceptor identified. Alert for SN2 identified. Nucleophilic Aliphatic Substitution	Alert for SN1 identified. Alert for Michael Acceptor identified.
5Ea1	Not corrosive	No skin corrosion	Negative	Negative	Class2 (persistent chemical)	Alert for Michael Acceptor identified. Alert for SN2 identified. Nucleophilic Aliphatic Substitution	Alert for Michael Acceptor identified
5Ea2	Not corrosive	No skin corrosion	Negative	Negative	Class2 (persistent chemical)	Alert for Michael Acceptor identified. Alert for SN2 identified. Nucleophilic Aliphatic Substitution	Alert for Michael Acceptor identified
5Ea3	Not irritating or corrosive	No skin corrosion	Negative	Negative	Class2 (persistent chemical)	Alert for Michael Acceptor identified. Alert for SN2 identified. Nucleophilic Aliphatic Substitution	Alert for Michael Acceptor identified

Table S9. Permeability and interactions with P-gp. Enzyme inhibitory effect on isoforms of cytochrome P450.

ID code	GI absorption	BBB permeant	P-gp substrate	CYP1A2 inhibitor	CYP2C19 inhibitor	CYP2C9 inhibitor	CYP2D6 inhibitor	CYP3A4 inhibitor	log Kp (cm/s)
1Aa1	High	No	No	No	No	No	No	No	-8.92
1Aa2	High	No	No	No	No	No	No	No	-9.71
1Aa3	High	No	No	No	No	No	No	No	-8.75
1Aa4	High	No	No	No	No	No	No	No	-9.53
1Aa5	High	No	No	No	No	No	No	No	-9.67
1Aa6	High	No	No	No	No	No	No	No	-9.67
1Aa7	High	No	No	No	No	No	No	No	-9.5
1Aa8	High	No	No	No	No	No	No	No	-9.37
1Ba1	High	No	No	No	No	No	No	No	-9.43
1Ba2	High	No	No	No	No	No	No	No	-8.26
1Ba3	High	No	No	No	No	No	No	No	-10.27
1Ca1	High	No	No	No	No	No	No	No	-9.23
1Ca2	High	No	No	No	No	No	No	No	-6.9
1Ca3	High	No	No	No	No	No	No	No	-9.23
1Da1	High	No	No	No	No	No	No	No	-9.77
1Da2	High	No	No	No	No	No	No	No	-10
1Da3	High	No	No	No	No	No	No	No	-9.87
1Da4	High	No	No	No	No	No	No	No	-7.73
2Aa1	High	No	No	No	No	No	No	No	-8.75
2Ba1	High	No	No	No	No	No	No	No	-9.41
2Ba2	High	No	No	No	No	No	No	No	-9.16
2Ba3	High	No	No	No	No	No	No	No	-7.63
2Ba4	High	No	No	No	No	No	No	No	-9.24
2Ba5	High	No	No	No	No	No	No	No	-8.82
2Ba6	High	No	No	No	No	No	No	No	-8.99

ID code	GI absorption	BBB permeant	P-gp substrate	CYP1A2 inhibitor	CYP2C19 inhibitor	CYP2C9 inhibitor	CYP2D6 inhibitor	CYP3A4 inhibitor	log Kp (cm/s)
2Ba6a	High	No	No	No	No	No	No	No	-8.92
2Ca1	High	No	No	No	No	No	No	No	-7.89
3Aa1	High	No	No	No	No	No	No	No	-7.17
3Aa2	High	No	No	No	No	No	No	No	-7.28
3Aa3	High	No	No	No	No	No	No	No	-9.26
3Aa4	High	No	No	No	No	No	No	No	-9.78
3Aa5	High	No	No	No	No	No	No	No	-9.26
3Aa6	High	No	No	No	No	No	No	No	-9.78
3Aa7	High	No	No	No	No	No	No	No	-7.19
3Aa8	High	No	No	No	No	No	No	No	-7.44
3Aa9	High	No	No	No	No	No	No	No	-6.94
3Ab10	High	No	No	No	No	No	No	No	-6.83
3Ba1	High	No	No	No	No	No	No	No	-7.51
3Ba2	High	No	No	No	No	No	No	No	-9.6
3Ba3	High	No	No	No	No	No	No	No	-9.6
3Ba4	High	No	No	No	No	No	No	No	-10.09
3Ba5	High	No	No	No	No	No	No	No	-7.63
3Ba6	High	No	No	No	No	No	No	No	-7.53
3Bb7	High	No	No	No	No	No	No	No	-7.23
3Bb8	High	No	No	No	No	No	No	No	-7.17
4Aa1	High	Yes	No	No	No	No	No	No	-6.59
4Aa2	High	Yes	No	No	No	No	No	No	-7.67
4Aa3	High	No	No	No	No	No	No	No	-9.47
4Ab1	Low	No	No	No	No	No	No	No	-8.42
4Ab2	High	No	No	No	No	No	No	No	-10.37
4Ac1	High	No	No	No	No	No	No	No	-11.29

ID code	GI absorption	BBB permeant	P-gp substrate	CYP1A2 inhibitor	CYP2C19 inhibitor	CYP2C9 inhibitor	CYP2D6 inhibitor	CYP3A4 inhibitor	log Kp (cm/s)
4Ad1	High	No	No	No	No	No	No	No	-8.77
4Ad2	High	No	No	No	No	No	No	No	-9.09
4Ad3	High	No	No	No	No	No	No	No	-8.75
4Ae1	High	Yes	No	No	No	No	No	No	-9.39
4Ae2	High	No	No	No	No	No	No	No	-9.2
4Ba1	Low	No	No	No	No	No	No	No	-9.71
4Ba2	High	No	No	No	No	No	No	No	-9.8
4Ba3	High	No	No	No	No	No	No	No	-7.77
4Ba4	High	No	No	No	No	No	No	No	-9.94
4Ba5	High	No	No	No	No	No	No	No	-10.04
4Ba6	High	No	No	No	No	No	No	No	-7.83
4Ba7	High	No	No	No	No	No	No	No	-8.17
4Ca1	High	No	No	No	No	No	No	No	-7.8
4Ca2	High	No	No	No	No	No	No	No	-7.95
4Ca3	High	No	No	No	No	No	No	No	-7.87
4Ca4	High	No	No	No	No	No	No	No	-8.02
4Cb1	High	No	No	No	No	No	No	No	-8.05
4Cb2	High	No	No	No	No	No	No	No	-10.1
4Cb3	High	No	No	No	No	No	No	No	-10.23
4Cb4	High	No	No	No	No	No	No	No	-9.85
4Cb5	High	No	No	No	No	No	No	No	-7.88
4Cb6	High	No	No	No	No	No	No	No	-10.32
4Cb7	High	No	No	No	No	No	No	No	-9.93
4Da1	High	No	No	No	No	No	No	No	-10.18
4Da2	High	No	No	No	No	No	No	No	-9.31
4Da3	High	No	No	No	No	No	No	No	-9.28

ID code	GI absorption	BBB permeant	P-gp substrate	CYP1A2 inhibitor	CYP2C19 inhibitor	CYP2C9 inhibitor	CYP2D6 inhibitor	CYP3A4 inhibitor	log Kp (cm/s)
4Da4	High	No	No	No	No	No	No	No	-9.84
4Da5	High	No	No	No	No	No	No	No	-9.2
4Da6	High	No	No	No	No	No	No	No	-9.2
4Da7	High	No	No	No	No	No	No	No	-8.9
4Da8	High	No	No	No	No	No	No	No	-8.66
4Da9	High	No	No	No	No	No	No	No	-8.86
4Da10	High	No	No	No	No	No	No	No	-9.13
4Da11	High	No	No	No	No	No	No	No	-8.74
4Db1	High	No	No	No	No	No	No	No	-10.24
4Db2	High	No	No	No	No	No	No	No	-10.32
4Db3	High	No	No	No	No	No	No	No	-10.16
4Db4	High	No	No	No	No	No	No	No	-10.55
4Db5	High	No	No	No	No	No	No	No	-10.31
4Db6	High	No	No	No	No	No	No	No	-10.15
4Dc1	Low	No	No	No	No	No	No	No	-11.65
4Dc2	Low	No	No	No	No	No	No	No	-11.98
4Dc3	Low	No	No	No	No	No	No	No	-11.98
4Dc4	High	No	No	No	No	No	No	No	-11.27
4Dc5	High	No	No	No	No	No	No	No	-11.59
4Dc6	Low	No	Yes	No	No	No	No	No	-14.39
4Dc7	Low	No	Yes	No	No	No	No	No	-14.22
4Dc8	Low	No	Yes	No	No	No	No	No	-13.52
4Dd1.1	High	No	No	No	No	No	No	No	-10.32
4Dd1.2	High	No	No	No	No	No	No	No	-9.69
4Dd1.3	High	No	No	No	No	No	No	No	-9.19
4Dd1.4	High	No	No	No	No	No	No	No	-9.85

ID code	GI absorption	BBB permeant	P-gp substrate	CYP1A2 inhibitor	CYP2C19 inhibitor	CYP2C9 inhibitor	CYP2D6 inhibitor	CYP3A4 inhibitor	log Kp (cm/s)
4Dd2.1	High	No	No	No	No	No	No	No	-9.64
4Dd3.1	High	No	No	No	No	No	No	No	-9.06
4Dd4.1	Low	No	Yes	No	No	No	No	No	-11.61
4Dd4.2	Low	No	Yes	No	No	No	No	No	-11.44
4Dd4.3	Low	No	No	No	No	No	No	No	-11.71
4Dd4.m	High	No	No	No	No	No	No	No	-10.37
5Aa1	Low	No	Yes	No	No	No	No	No	-10.29
5Aa2	High	No	Yes	No	No	Yes	Yes	Yes	-8.13
5Aa3	Low	No	Yes	No	No	No	No	No	-10.46
5Aa4	Low	No	Yes	No	No	No	No	No	-10.46
5Ba1	Low	No	Yes	No	Yes	No	No	No	-8.41
5Ba2	Low	No	Yes	No	Yes	No	No	No	-8.41
5Ca1	High	No	Yes	No	No	Yes	No	No	-8.29
5Ca2	High	No	Yes	No	No	Yes	Yes	No	-8.29
5Da1	High	No	No	No	No	No	No	No	-10.26
5Da2	High	No	No	No	No	No	No	No	-10.26
5Da3	Low	No	No	No	No	No	No	No	-12.98
5Ea1	High	No	No	No	No	No	No	No	-11.1
5Ea2	High	No	No	No	No	No	No	No	-11.1
5Ea3	Low	No	No	No	No	No	No	No	-13.82

Table S10. The number of broken rules, according to Lipinski, Ghose, Veber, Egan and Muegge and the bioavailability score, the drug-likeness score, the lead-likeness score and the synthetic accessibility score.

ID code	Lipinski	Ghose	Veber	Egan	Muegge	BD Score	Drug-likeness	Overall drug-likeness score	CNS MPO	>=4	Lead-likeness	SA
1Aa1	0	4	0	0	2	0.55	-17.68	0.49	4.95	1	1	2.1
1Aa2	0	4	0	0	2	0.55	-17.52	0.49	5.25	1	1	2.1
1Aa3	0	2	0	0	2	0.55	-22.37	0.49	4.95	1	1	2.3
1Aa4	0	2	0	0	2	0.55	-20.24	0.49	5.25	1	1	2.3
1Aa5	0	2	0	0	2	0.55	-26.1	0.49	4.49	1	1	2
1Aa6	0	2	0	0	2	0.55	-22.18	0.49	5.25	1	1	2
1Aa7	0	0	0	0	2	0.55	-31.48	0.49	4.49	1	1	2.2
1Aa8	0	0	0	0	2	0.55	-24.86	0.49	5.25	1	1	2.2
1Ba1	0	3	0	0	2	0.55	-17.95	0.3	3.8	0	1	1.8
1Ba2	0	3	0	0	1	0.55	-7.17	0.49	4.14	1	1	1.7
1Ba3	0	3	0	0	2	0.55	-7.32	0.49	4.22	1	1	1.8
1Ca1	0	2	0	0	2	0.55	-33.22	0.24	4.69	1	1	2.1
1Ca2	0	2	0	0	1	0.55	-23.39	0.24	5.5	1	1	2.3
1Ca3	0	2	0	0	2	0.55	-17.54	0.24	5.25	1	1	2
1Da1	0	3	0	0	2	0.55	-30.73	0.3	4.69	1	1	2.2
1Da2	0	1	0	0	2	0.55	-31.63	0.29	3.94	0	1	2.3
1Da3	0	1	0	0	2	0.55	-31.19	0.29	4.69	1	1	2.3
1Da4	0	4	0	0	1	0.55	-36.21	0.3	5.5	1	1	2
2Aa1	0	1	0	0	1	0.56	-14.6	0.49	4.79	1	1	2
2Ba1	0	2	0	0	2	0.56	-1.7	0.57	4.25	1	1	1.9
2Ba2	0	0	0	0	2	0.56	-0.78	0.65	4.5	1	1	2
2Ba3	0	0	0	0	1	0.85	-0.78	0.65	5.5	1	1	2.2
2Ba4	0	0	0	0	2	0.56	-1.92	0.55	4.25	1	1	2
2Ba5	0	0	0	0	0	0.56	-0.59	0.66	4.5	1	1	2.2

ID code	Lipinski	Ghose	Veber	Egan	Muegge	BD Score	Drug-likeness	Overall drug-likeness score	CNS MPO	>=4	Lead-likeness	SA
2Ba6	0	0	0	0	2	0.56	-0.5	0.68	4.5	1	1	2.1
2Ba6a	0	0	0	0	0	0.56	0.91	0.18	5.5	1	1	2.6
2Ca1	0	4	0	0	1	0.56	-3.26	0.51	5.09	1	1	2.3
3Aa1	0	0	0	0	0	0.56	-1.22	0.59	4.93	1	1	3
3Aa2	0	0	0	0	0	0.56	-1.06	0.61	5.5	1	1	3.5
3Aa3	0	0	0	0	1	0.55	-16.32	0.49	4.58	1	1	2.9
3Aa4	0	0	0	0	1	0.55	-15.66	0.48	4.59	1	1	3.3
3Aa5	0	0	0	0	1	0.55	-6.07	0.49	5.14	1	1	2.9
3Aa6	0	0	0	0	1	0.55	-5.03	0.49	5.03	1	1	3.4
3Aa7	0	0	0	0	1	0.55	-3.12	0.49	6	1	1	2.1
3Aa8	0	0	0	0	1	0.55	3.86	0.96	5.75	1	1	2.8
3Aa9	0	0	0	0	1	0.55	-14.51	0.29	6	1	1	3.3
3Ab10	0	0	0	0	0	0.55	-14.67	0.29	5.75	1	1	3
3Ba1	0	0	0	0	0	0.56	-1.92	0.54	4.49	1	1	2.9
3Ba2	0	0	0	0	2	0.55	-17.27	0.48	4.15	1	1	2.8
3Ba3	0	0	0	0	2	0.55	-7.02	0.48	4.7	1	1	2.8
3Ba4	0	0	0	1	1	0.55	-15.57	0.48	3.91	0	1	3.4
3Ba5	0	1	0	0	1	0.55	3.8	0.96	5.75	1	1	3.2
3Ba6	0	0	0	0	1	0.55	-3.85	0.48	6	1	1	2.1
3Bb7	0	1	0	0	1	0.55	-15.32	0.29	6	1	1	3.2
3Bb8	0	0	0	0	1	0.55	-15.57	0.29	5.75	1	1	2.8
4Aa1	0	0	0	0	0	0.56	2.91	0.17	5.42	1	1	2.6
4Aa2	0	0	0	0	0	0.55	-13.53	0.09	4.49	1	1	2.5
4Aa3	0	0	0	0	1	0.55	-9.27	0.1	4.49	1	1	2.3
4Ab1	0	1	1	1	1	0.11	-1.56	0.1	4	0	1	2.6
4Ab2	0	2	0	0	2	0.55	-17.31	0.11	3.35	0	1	2.2

ID code	Lipinski	Ghose	Veber	Egan	Muegge	BD Score	Drug-likeness	Overall drug-likeness score	CNS MPO	>=4	Lead-likeness	SA
4Ac1	0	1	0	0	2	0.56	-1.58	0.22	4.18	1	1	2.7
4Ad1	0	0	0	0	0	0.55	0.07	0.21	4.74	1	1	3.7
4Ad2	0	0	0	0	0	0.55	0.17	0.27	4.49	1	1	3.1
4Ad3	0	0	0	0	0	0.55	-1.76	0.16	4.01	1	1	3.4
4Ae1	0	1	0	0	0	0.55	-11.15	0.28	4.51	1	1	2.4
4Ae2	0	1	0	0	0	0.55	-7.44	0.28	4.51	1	1	3
4Ba1	0	3	0	0	2	0.55	-19.43	0.39	4.39	1	1	2.9
4Ba2	0	2	0	0	2	0.55	-19.25	0.49	4.39	1	1	3
4Ba3	0	4	0	0	1	0.55	-0.96	0.63	5.5	1	1	2.2
4Ba4	0	1	0	0	1	0.55	-20.61	0.39	3.63	0	2	2.8
4Ba5	0	1	0	0	1	0.55	-20.39	0.49	3.63	0	2	2.9
4Ba6	0	1	0	0	0	0.55	-9.1	0.38	6	1	1	2
4Ba7	0	1	0	0	0	0.55	-3.07	0.51	6	1	1	1.6
4Ca1	0	2	0	0	1	0.55	-2.34	0.54	3.89	0	1	2.4
4Ca2	0	2	0	0	1	0.55	-4.65	0.5	3.89	0	1	1.7
4Ca3	0	3	0	0	1	0.56	-1.1	0.61	5.25	1	1	2.2
4Ca4	0	3	0	0	1	0.56	-5.74	0.49	5.25	1	1	1.6
4Cb1	0	3	0	0	1	0.55	0.01	0.74	3.93	0	1	2.6
4Cb2	0	3	0	0	2	0.55	-2.98	0.52	3.26	0	1	2.9
4Cb3	0	3	0	0	2	0.55	1.73	0.35	3.33	0	1	2.8
4Cb4	0	3	0	0	2	0.55	2.74	0.28	4.71	1	1	3.1
4Cb5	0	2	0	0	1	0.55	-1.81	0.56	3.9	0	1	2.6
4Cb6	0	1	0	0	2	0.55	-4.79	0.5	3.04	0	1	2.8
4Cb7	0	3	0	0	2	0.55	-1	0.63	4.21	1	1	2.9
4Da1	0	0	0	1	2	0.55	-13.79	0.49	3.79	0	1	3.3
4Da2	0	0	0	1	2	0.55	-9.5	0.39	3.87	0	1	3.7

ID code	Lipinski	Ghose	Veber	Egan	Muegge	BD Score	Drug-likeness	Overall drug-likeness score	CNS MPO	>=4	Lead-likeness	SA
4Da3	0	0	0	1	2	0.55	-12.19	0.39	3.83	0	1	3.8
4Da4	0	0	0	1	2	0.55	-15.06	0.49	3.24	0	1	3.6
4Da5	0	0	0	1	2	0.55	-9.43	0.39	3.25	0	1	4.1
4Da6	0	0	0	1	2	0.55	-12.12	0.39	3.21	0	1	4.3
4Da7	0	0	0	0	2	0.55	-2.39	0.43	4.53	1	1	4.1
4Da8	0	0	0	0	1	0.55	2.91	0.37	5.5	1	1	4.1
4Da9	0	0	0	0	2	0.55	2.39	0.36	4.91	1	1	4.2
4Da10	0	0	0	0	2	0.55	-3.98	0.4	3.97	0	1	3.9
4Da11	0	0	0	0	1	0.55	-0.26	0.56	5.24	1	1	4.1
4Db1	0	1	0	0	2	0.55	-10.57	0.19	5.03	1	1	3.6
4Db2	0	2	0	0	2	0.55	-15.66	0.4	3.85	0	1	4.2
4Db3	0	1	0	0	2	0.55	-13.1	0.4	4.63	1	1	4
4Db4	0	0	0	0	2	0.55	-17.53	0.39	3.86	0	1	4
4Db5	0	1	0	0	2	0.55	-12.17	0.39	4.25	1	1	3.8
4Db6	0	0	0	0	1	0.55	-12.33	0.39	4.25	1	1	3.8
4Dc1	0	0	1	1	2	0.11	-19.52	0.49	3	0	1	4
4Dc2	0	1	1	1	2	0.11	-14.83	0.49	3	0	1	4
4Dc3	0	1	1	1	2	0.11	-13.59	0.39	3	0	1	3.9
4Dc4	0	0	0	0	1	0.55	-15.89	0.39	3.68	0	1	4.9
4Dc5	0	0	0	0	1	0.55	-15.48	0.49	3.65	0	1	4.3
4Dc6	1	1	1	1	3	0.11	-13.01	0.48	3.59	0	0	3.9
4Dc7	1	1	1	1	3	0.11	-11.31	0.48	4	0	0	3.9
4Dc8	1	1	1	1	3	0.11	-11.68	0.49	3.38	0	0	3.9
4Dd1.1	0	3	0	0	2	0.55	-12.63	0.49	4.05	1	1	2.1
4Dd1.2	0	3	0	0	2	0.55	-2.8	0.52	4.44	1	1	2.5
4Dd1.3	0	3	0	0	2	0.55	-4.53	0.5	4.58	1	1	2.7

ID code	Lipinski	Ghose	Veber	Egan	Muegge	BD Score	Drug-likeness	Overall drug-likeness score	CNS MPO	>=4	Lead-likeness	SA
4Dd1.4	0	3	0	0	2	0.55	1.89	0.92	4.02	1	1	2.4
4Dd2.1	0	2	0	0	2	0.55	-12.96	0.09	3.36	0	1	2.5
4Dd3.1	0	4	0	0	2	0.55	-0.88	0.64	4.43	1	1	2.5
4Dd4.1	1	1	1	1	3	0.55	-11.98	0.48	3.78	0	1	3.4
4Dd4.2	1	1	1	1	3	0.55	-17.92	0.47	3.78	0	1	3.6
4Dd4.3	1	1	2	1	3	0.11	-14.91	0.47	3.78	0	1	3.4
4Dd4.m	0	1	0	0	1	0.55	-13.43	0.49	3.48	0	1	2.3
5Aa1	0	1	1	1	0	0.56	-0.44	0.3	2.34	0	2	4.6
5Aa2	0	0	0	0	0	0.55	-2.07	0.25	5.29	1	1	4.1
5Aa3	2	3	2	1	1	0.17	-18.6	0.19	2.24	0	2	5.7
5Aa4	2	3	2	1	1	0.17	-17.94	0.19	3	0	2	5.7
5Ba1	2	3	2	1	3	0.17	-14.01	0.15	2.26	0	3	6.2
5Ba2	2	3	2	1	3	0.17	-13.35	0.15	2.81	0	3	6.2
5Ca1	0	0	0	0	0	0.55	-16.76	0.04	3.22	0	2	3.8
5Ca2	0	0	0	0	0	0.55	-16.07	0.04	3.98	0	2	3.8
5Da1	0	0	0	0	0	0.55	-17.14	0.4	2.72	0	1	4.8
5Da2	0	0	0	0	0	0.55	-16.85	0.4	2.76	0	1	4.8
5Da3	2	3	2	1	3	0.17	-17.3	0.3	2.03	0	2	5.6
5Ea1	0	0	0	0	1	0.55	-16.7	0.4	0	0	1	5.2
5Ea2	0	0	0	0	1	0.55	-16.7	0.4	0	0	1	5.2
5Ea3	2	3	2	1	3	0.17	-16.85	0.31	2.09	0	2	5.9

Figure S1. Homododecameric structure of the bacterial GS enzyme and D subunit, which was tested during the molecular docking study [31]. Two manganese ions and an AMP residue can be seen in the active site of subunit D. The structures were generated using UCSF Chimera software based on the PDB code of GS taken from the Protein Data Bank [31,32].

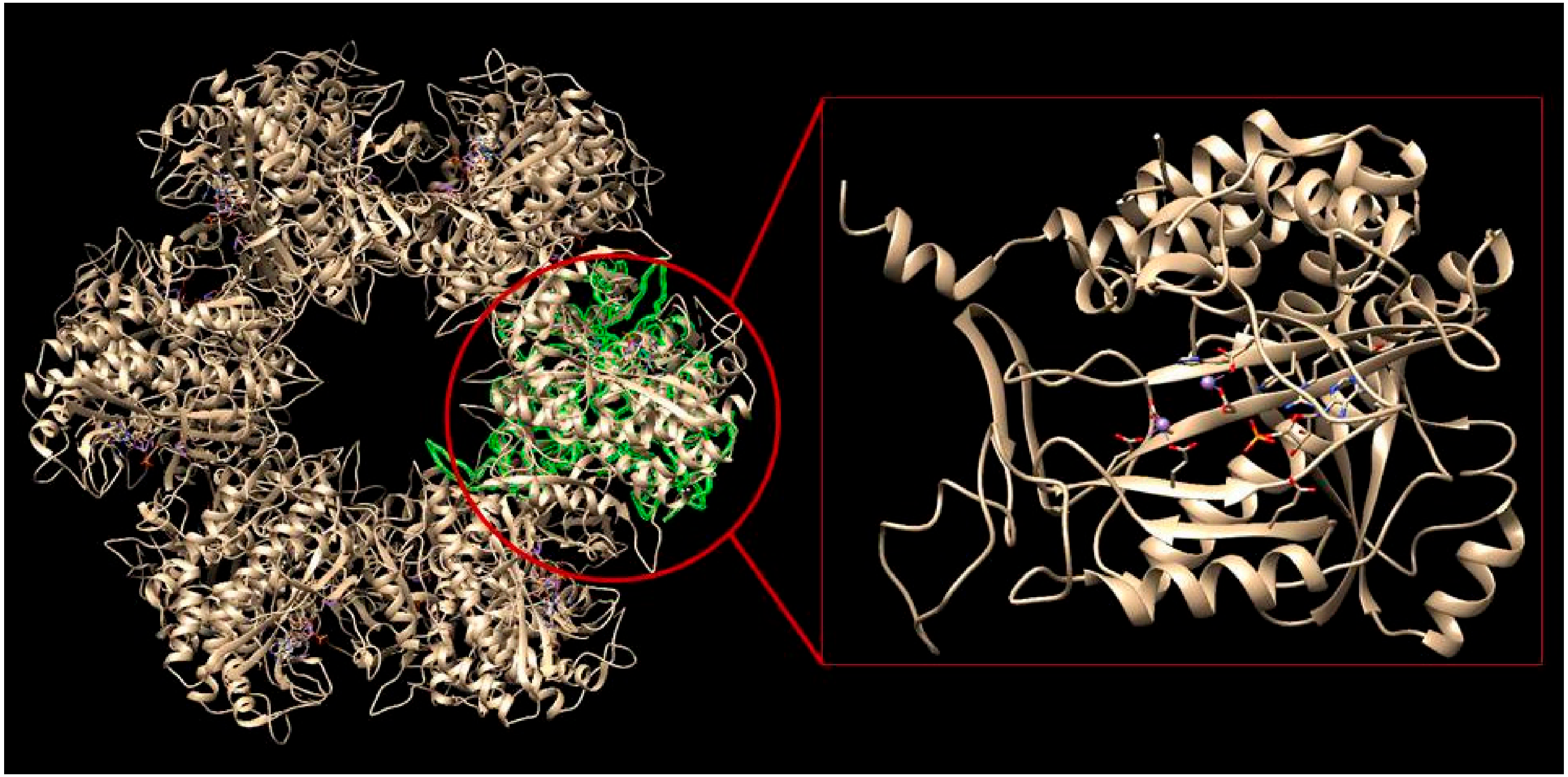

Figure S2. Molecular docking results visualized using UCSF Chimera and AutoDock Vina (Webina) [31,33,34]

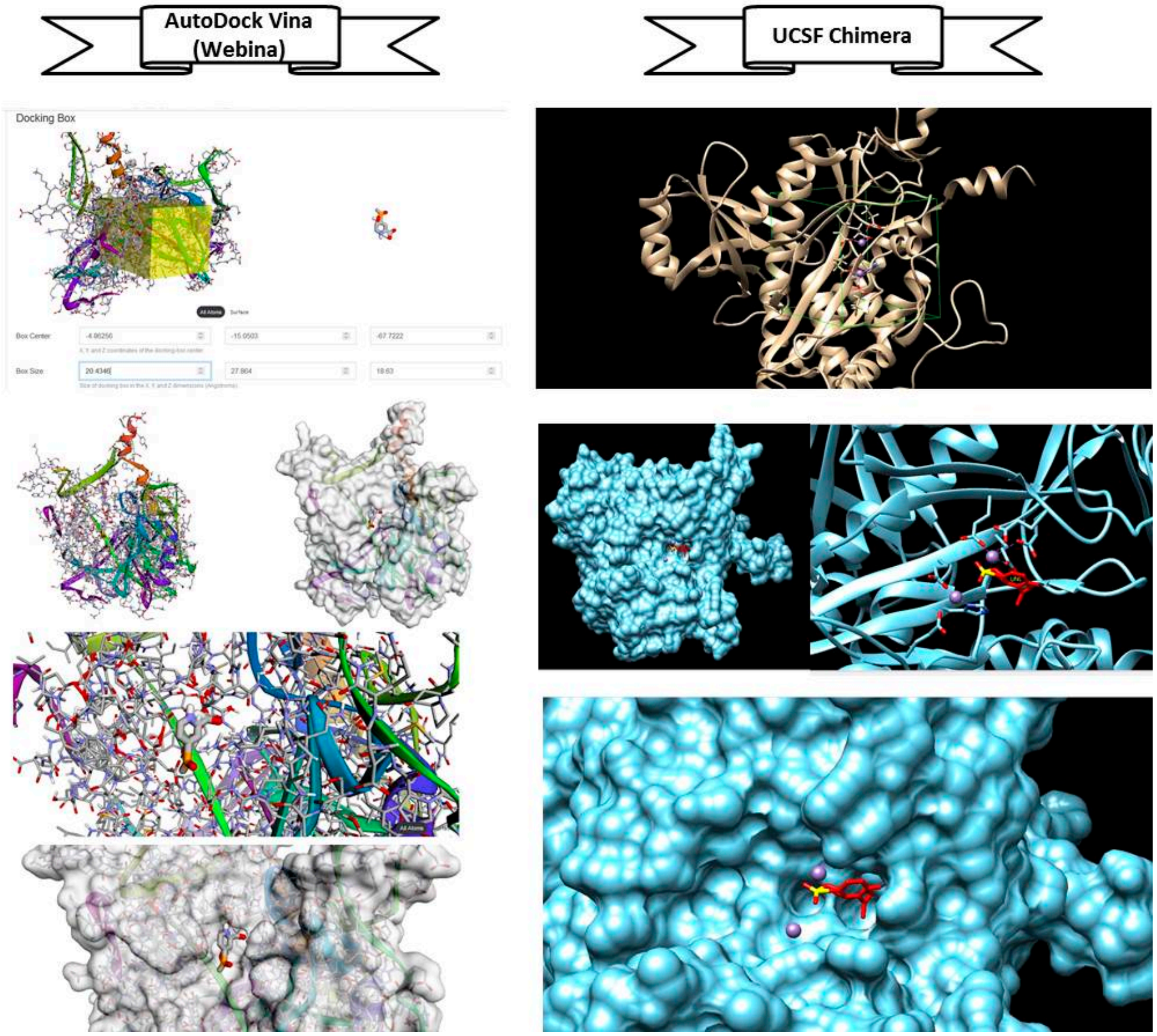

Table S11. Chemical structures of colchicine, neferine, 7-hydroxynuciferine, lycorine, 5,6-dehydrolycorine [35].

**Colchicine**	**Neferine**	**7-hydroxynuciferine**
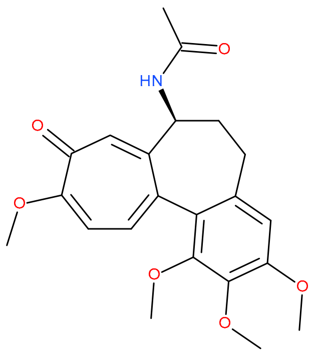	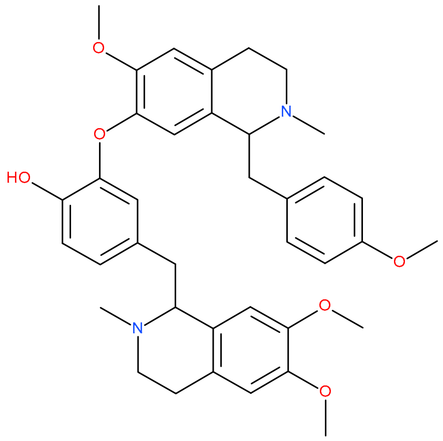	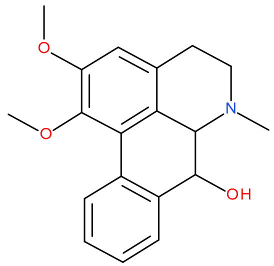
**Lycorine**	**5,6-dehiydrolycorine**	
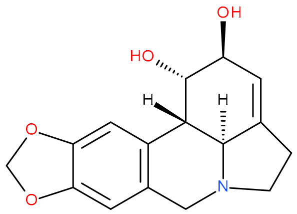	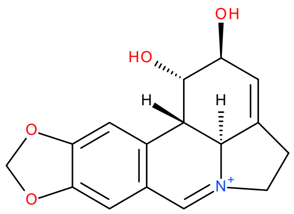	

Table S12. Toxicity comparison of vegetal compounds and their complexes with glutamic acid [1,6,36].

Table S12 (A). Toxicity (I) Cramer rules, Kroess and Verhaar scheme.

**ID code/ name**	**Cramer**	**Kroess TTC**		**Verhaar scheme**
	**Toxic Hazard**	**Estimate**	**Explanation**	
**5Aa1**	Class I	Substance would not be expected to be a safety concern	Verify structural alerts for potential genotoxic carcinogenicity	Class 5
**5Aa2**	Class III	Substance would not be expected to be a safety concern	Verify structural alerts for potential genotoxic carcinogenicity	Class 5
**5Aa3**	Class I	Substance would not be expected to be a safety concern	Verify structural alerts for potential genotoxic carcinogenicity	Class 5
**5Aa4**	Class I	Substance would not be expected to be a safety concern	Verify structural alerts for potential genotoxic carcinogenicity	Class 5
**5Ba1**	Class III	Substance would not be expected to be a safety concern	Verify structural alerts for potential genotoxic carcinogenicity	Class 5
**5Ba2**	Class III	Substance would not be expected to be a safety concern	Verify structural alerts for potential genotoxic carcinogenicity	Class 5
**5Ca1**	Class III	Negligible risk (low probability of a life-time cancer risk greater than 1 in 10^6)	Genotoxic potential. Polycyclic Aromatic Hydrocarbons	Class 5
**5Ca2**	Class III	Negligible risk (low probability of a life-time cancer risk greater than 1 in 10^6)	Genotoxic potential. Polycyclic Aromatic Hydrocarbons	Class 5
**5Da1**	Class III	Substance would not be expected to be a safety concern	Verify structural alerts for potential genotoxic carcinogenicity	Class 5
**5Da2**	Class III	Substance would not be expected to be a safety concern	Verify structural alerts for potential genotoxic carcinogenicity	Class 5
**5Da3**	Class III	Substance would not be expected to be a safety concern	Verify structural alerts for potential genotoxic carcinogenicity	Class 5
**5Ea1**	Class III	Substance would not be expected to be a safety concern	Verify structural alerts for potential genotoxic carcinogenicity	Class 5
**5Ea2**	Class III	Substance would not be expected to be a safety concern	Verify structural alerts for potential genotoxic carcinogenicity	Class 5

ID code/ name	Cramer	Kroess TTC		Verhaar scheme
	Toxic Hazard	Estimate	Explanation	
**5Ea3**	Class III	Substance would not be expected to be a safety concern	Verify structural alerts for potential genotoxic carcinogenicity	Class 5
**Colchicine**	Class I	Substance would not be expected to be a safety concern	Verify structural alerts for potential genotoxic carcinogenicity	Class 5
**Neferine**	Class III	Substance would not be expected to be a safety concern	Verify structural alerts for potential genotoxic carcinogenicity	Class 5
**7-hydroxynuciferine**	Class III	Substance would not be expected to be a safety concern	Verify structural alerts for potential genotoxic carcinogenicity	Class 3
**Lycorine**	Class III	Substance would not be expected to be a safety concern	Verify structural alerts for potential genotoxic carcinogenicity	Class 3
**5,6-dehydrolycorine**	Class III	Substance would not be expected to be a safety concern	Verify structural alerts for potential genotoxic carcinogenicity	Class 3

Table S12 (B). Toxicity (II) Carcinogenic (genotoxic and non-genotoxic) and mutagenic effects evaluated using two different apps (Toxtree and OSIRIS).

ID code/name	Carcinogenicity			Mutagenicity	
	Genotox. Toxtree	Non-genotox.	Tumorigen. OSIRIS	Toxtree (Ames test) *S. typhimurium*	OSIRIS
**5Aa1**	Negative	Negative	Negative	Negative	Negative
**5Aa2**	Negative	Negative	Negative	Negative	Negative
**5Aa3**	Negative	Negative	Negative	Negative	Negative
**5Aa4**	Negative	Negative	Negative	Negative	Negative
**5Ba1**	Negative	Negative	Negative	Negative	Negative
**5Ba2**	Negative	Negative	Negative	Negative	Negative

ID code/name	Carcinogenicity			Mutagenicity	
	**Genotox. Toxtree**	**Non-genotox.**	**Tumorigen. OSIRIS**	**Toxtree (Ames test) *S. typhimurium***	**OSIRIS**
**5Ca1**	Structural Alert for genotoxic carcinogenicity	Structural Alert for nongenotoxic carcinogenicity	High risk	Structural alert for *S. typhimurium* mutagenicity	High risk
**5Ca2**	Structural Alert for genotoxic carcinogenicity	Structural Alert for nongenotoxic carcinogenicity	High risk	Structural alert for *S. typhimurium* mutagenicity	High risk
**5Da1**	Negative	Structural Alert for nongenotoxic carcinogenicity	Negative	Negative	Negative
**5Da2**	Negative	Structural Alert for nongenotoxic carcinogenicity	Negative	Negative	Negative
**5Da3**	Negative	Structural Alert for nongenotoxic carcinogenicity	Negative	Negative	Negative
**5Ea1**	Negative	Structural Alert for nongenotoxic carcinogenicity	Negative	Negative	Negative
**5Ea2**	Negative	Structural Alert for nongenotoxic carcinogenicity	Negative	Negative	Negative
**5Ea3**	Negative	Structural Alert for nongenotoxic carcinogenicity	Negative	Negative	Negative
**Colchicine**	Negative	Negative	Negative	Negative	Negative
**Neferine**	Negative	Negative	Negative	Negative	Negative
**7-hydroxynuciferine**	Negative	Structural Alert for nongenotoxic carcinogenicity	Negative	Structural alert for *S. typhimurium* mutagenicity	Negative
**Lycorine**	Negative	Structural Alert for nongenotoxic carcinogenicity	Negative	Negative	Negative
**5,6-dehydrolycorine**	Negative	Structural Alert for nongenotoxic carcinogenicity	Unknown	Negative	Unknown

Table S12 (C). Toxicity (III) Irritant/corrosive effect on the skin and eyes, effect on the reproductive system.

ID code/name	Irritation/corrosion			Effect on the reproductive system
	**Skin**	**Eye**	**OSIRIS**	**OSIRIS**
**5Aa1**	Not corrosive	No skin corrosion	Negative	High risk
**5Aa2**	Not corrosive	No skin corrosion	Negative	High risk

ID code/name	Irritation/corrosion			Effect on the reproductive system
	Skin	Eye	OSIRIS	OSIRIS
**5Aa3**	Not corrosive	No skin corrosion	Negative	High risk
**5Aa4**	Not corrosive	No skin corrosion	Negative	High risk
**5Ba1**	Not corrosive	No eye irritation	Negative	Negative
**5Ba2**	Not corrosive	No eye irritation	Negative	Negative
**5Ca1**	Not corrosive	No skin corrosion	High risk	High risk
**5Ca2**	Not corrosive	No skin corrosion	High risk	High risk
**5Da1**	Not corrosive	No skin corrosion	Negative	Negative
**5Da2**	Not corrosive	No skin corrosion	Negative	Negative
**5Da3**	Not corrosive	No skin corrosion	Negative	Negative
**5Ea1**	Not corrosive	No skin corrosion	Negative	Negative
**5Ea2**	Not corrosive	No skin corrosion	Negative	Negative
**5Ea3**	Not irritating or corrosive	No skin corrosion	Negative	Negative
**Colchicine**	Not corrosive	No skin corrosion	Negative	High risk
**Neferine**	Not corrosive	No skin corrosion	Negative	Negative
**7-hydroxynuciferine**	Not corrosive	No skin corrosion	Negative	Negative
**Lycorine**	Irritating	Unknown	Negative	Negative
**5,6-dehydrolycorine**	Irritating	Unknown	Unknown	Unknown

Table S13. Characterization of phosphinothricin, methionine sulfoximine, glutamic acid and ampicillin.

Table S13 (A). Chemical structures and computational descriptors of phosphinothricin, methionine sulfoximine, glutamic acid and ampicillin, obtained with BioviaDraw.

Compound	Structural formula	SMILES	Molecular weight	Molecular formula	IUPAC name	InChI String	InChI Key
**Phosphinothricin**	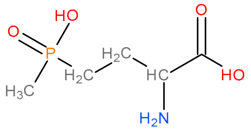	CP(=O)(O)CCC(N)C(=O)O	181.13	$C_5H_{12}NO_4P$	2-amino-4-[hydroxy(methyl)phosphoryl]butanoic acid	InChI=1S/C5H12NO4P/c1-11(9,10)3-2-4(6)5(7)8/h4H,2-3,6H2,1H3,(H,7,8)(H,9,10)	IAJOBQBIJHVGMQ-UHFFFAOYSA-N
**Methionine sulfoximine**	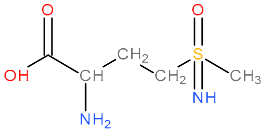	CS(=N)(=O)CCC(N)C(=O)O	180.23	C5H12N2O3S	2-amino-4-(methylsulfonimidoyl)butanoic acid	InChI=1S/C5H12N2O3S/c1-11(7,10)3-2-4(6)5(8)9/h4,7H,2-3,6H2,1H3,(H,8,9)	SXTAYKAGBXMACB-UHFFFAOYSA-N
**Glutamic acid**	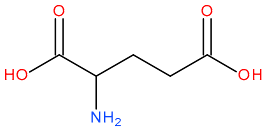	NC(CCC(=O)O)C(=O)O	147.13	$C_5H_9NO_4$	2-aminopentanedioic acid	InChI=1S/C5H9NO4/c6-3(5(9)10)1-2-4(7)8/h3H,1-2,6H2,(H,7,8)(H,9,10)	WHUUTDBJXJRKMK-UHFFFAOYSA-N
**Ampicillin**	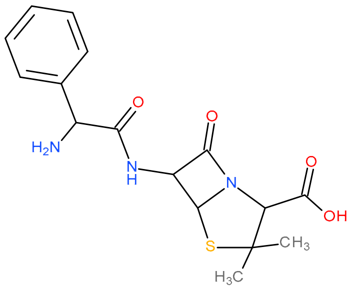	CC1(C)SC2C(NC(=O)C(N)c3ccccc3)C(=O)N2C1C(=O)O	349.40	$C_{16}H_{19}N_3O_4S$	6-[(2-amino-2-phenyl-acetyl)amino]-3,3-dimethyl-7-oxo-4-thia-1-azabicyclo[3.2.0]heptane-2-carboxylic acid	InChI=1S/C16H19N3O4S/c1-16(2)11(15(22)23)19-13(21)10(14(19)24-16)18-12(20)9(17)8-6-4-3-5-7-8/h3-7,9-11,14H,17H2,1-2H3,(H,18,20)(H,22,23)	AVKUERGKIZMTKX-UHFFFAOYSA-N

Table S13 (B). Structural and physicochemical properties. Protonation and electric charge.

Compound	Structural and physicochemical properties								Protonation					Electric charge
	HA	HAA	Fraction Csp3	RB	H-bond acceptors	H-bond donors	MR	TPSA	Acidic pKa	Basic pKa	pKa score	pI	Microspecies	Molar polarizability
**Phosphinothricin**	11	0	0.8	4	5	3	40.45	110.43	3.19	9.53	9.53	3.57	5	15.91
**Methionine sulfoximine**	11	0	0.8	4	5	3	42.30	112.62	2.22	9.09	9.09	6.10	5	16.92
**Glutamic acid**	10	0	0.6	4	5	3	32.40	100.62	2.58	9.54	9.54	3.52	5	12.69
**Ampicillin**	24	6	0.44	5	5	3	92.56	138.03	2.04	7.23	7.23	5.13	9	34.87

Table S13 (C). Water solubility.

Compound	logS AquaSol	ESOL Log S	ESOL Solub. (mg/ml)	ESOL Solub. (mol/l)	ESOL Class	Ali Log S	Ali Sol (mg/ml)	Ali Sol (mol/l)	Ali Class	Silicos-IT LogSw	Silicos-IT Sol. (mg/ml)	Silicos-IT Sol. (mol/l)	Silicos-IT class
**Phosphinothricin**	-0.28	2.48	54200	299	H	3.36	415000	2290	H	0.42	473	2.61	S
**Methionine sulfoximine**	-0.51	0.45	504	2.80	H	-0.04	165	0.916	V	-0.21	111	0.618	S
**Glutamic acid**	-0.27	1.84	10100	68.6	H	2.16	21500	146	H	0.89	1150	7.83	S
**Ampicillin**	-2.68	-1.15	24.8	0.0709	V	-1.28	18.5	0.0528	V	-2.21	2.15	0.00615	S

Table S13 (D). Lipophilicity - partition coefficients.

Compound	iLOGP	XLOGP	WLOGP	MLOGP	Silicos-IT Log P	Consensus Log P	logP	logD at pH 7.4	HLB
**Phosphinothricin**	-0.09	-5.04	-0.31	-3.18	-1.42	-2.01	-2.06	-6.44	18.60
**Methionine sulfoximine**	0.37	-1.81	0.33	-3.27	-0.74	-1.03	-2.18	-4.45	18.60
**Glutamic acid**	0.41	-3.69	-0.74	-3.18	-1.19	-1.68	-3.35	-5.59	17.98
**Ampicillin**	1.14	-1.13	-0.39	0.75	0.01	0.08	0.15	-2.68	25.70

Table S13 (E). Toxicity (I): Cramer rules, Kroess and Verhaar scheme.

Compound	Cramer rules	Kroess TTC		Verhaar scheme
	**Toxic hazard**	**Estimate**	**Explanation**	
**Phosphinothricin**	High-Class III	Risk assessment requires compound-specific toxicity data	Non-essential metal or metal-containing compound, or is it a polyhalogenated dibenzodioxin, -dibenzofuran, or -biphenyl compound	Class 5 (Not possible to classify according to these rules)
**Methionine sulfoximine**	High-Class III	Substance would not be expected to be a safety concern	Verify structural alerts for potential genotoxic carcinogenicity	Class 5 (Not possible to classify according to these rules)
**Glutamic acid**	Low (Class I)	Substance would not be expected to be a safety concern	Verify structural alerts for potential genotoxic carcinogenicity	Class 5 (Not possible to classify according to these rules)
**Ampicillin**	High-Class III	Substance would not be expected to be a safety concern	Verify structural alerts for potential genotoxic carcinogenicity	Class 5 (Not possible to classify according to these rules)

Table S13 (F). Toxicity (II). Carcinogenic (genotoxic and non-genotoxic) and mutagenic effects evaluated using two different apps (Toxtree and OSIRIS).

Compound	Carcinogenicity			Mutagenicity	
	**Genotoxic Toxtree**	**Non-genotoxic**	**Tumorigenesis OSIRIS**	**Toxtree (Ames test) *S. typhimurium***	**OSIRIS**
**Phosphinothricin**	Negative	Negative	Negative	Negative	Negative
**Methionine sulfoximine**	Negative	Negative	Negative	Negative	Negative
**Glutamic acid**	Negative	Negative	Negative	Negative	Negative

**Ampicillin**	Negative	Negative	Negative	Negative	Negative

Table S13 (G). Toxicity (III). Irritant/corrosive effect on the skin and eyes, effect on the reproductive system, biodegradability, protein and DNA binding alerts, assessed using Toxtree and OSIRIS.

**Compound**	**Irritation/corrosion**			**Effect on the reproductive system**	**Biodegradability**	**Protein binding Alerts**	**DNA binding Alerts**
	**Skin**	**Eye**	**OSIRIS**	**OSIRIS**			
**Phosphinothricin**	Not irritating or corrosive	Negative	Negative	Medium risk	Class1 (easily biodegradable chemical)	Negative	Negative
**Methionine sulfoximine**	Not irritating	Negative	Negative	Medium risk	Class1 (easily biodegradable chemical)	Negative	Negative
**Glutamic acid**	Not irritating or corrosive	Negative	Negative	Negative	Class1 (easily biodegradable chemical)	Negative	Negative
**Ampicillin**	Not irritating	Serious lesions to the eye	Negative	Negative	Class2 (persistent chemical)	Alert for Michael Acceptor, Acyl transfer agent, SN2 identified.	Alert for SN1, Michael Acceptor, Acyl transfer agent identified.

Table S13 (H). Permeability and interactions with P-gp. Enzyme inhibitory effect on isoforms of cytochrome P450.

**Compound**	**GI absorption**	**BBB permeant**	**P-gp substrate**	**CYP1A2 inhibitor**	**CYP2C19 inhibitor**	**CYP2C9 inhibitor**	**CYP2D6 inhibitor**	**CYP3A4 inhibitor**	**log Kp (cm/s)**
**Phosphinothricin**	High	No	No	No	No	No	No	No	-10.98
**Methionine sulfoximine**	High	No	No	No	No	No	No	No	-8.68
**Glutamic acid**	High	No	No	No	No	No	No	No	-9.82
**Ampicillin**	Low	No	No	No	No	No	No	No	-9.23

Table S13 (I). The number of broken rules, according to Lipinski, Ghose, Veber, Egan and Muegge and the bioavailability score, the drug-likeness score, the lead-likeness score and the synthetic accessibility score.

Compound	Lipinski	Ghose	Veber	Egan	Muegge	BD Score	Drug-likeness	Overall drug-likeness score	CNS MPO	>=4	Lead-likeness	SA
**Phosphinothricin**	0	0	0	0	2	0.55	-24.95	0.4	3.88	0	1	4.09
**Methionine sulfoximine**	0	0	0	0	1	0.55	-14.3	0.39	3.98	0	1	3.71
**Glutamic acid**	0	4	0	0	2	0.56	-18.65	0.49	3.88	0	1	1.81
**Ampicillin**	0	0	0	1	0	0.55	10.72	0.91	4.24	1	0	4.16

Table S13 (J). The geometrical and isomer-conformation properties of the compounds.

Compound	Geometric isomers		Isomers		Conformations
	Asymmetric atoms	Chiral centres	Tautomers	Stereoisomers	Emin (kcal/mol)
**Glutamic acid**	1	1	16	2	7.74
**Phosphinothricin**	1	1	21	2	54.07
**Methionine sulfoximine**	2	2	56	4	43.03
**Ampicillin**	4	4	36	16	107.24

Table S13 (K). Compound metabolism assessed using Toxtree.

Compound	Primary sites of metabolism	Secondary sites of metabolism	Tertiary sites of metabolism	Quaternary sites of metabolism
**Phosphinothricin**	N-dealkylation	Amine hydroxylation	Aliphatic hydroxylation	Aliphatic hydroxylation
**Methionine sulfoximine**	N-dealkylation	Amine hydroxylation	Aliphatic hydroxylation	Aliphatic hydroxylation
**Glutamic acid**	N-dealkylation	Amine hydroxylation	Aliphatic hydroxylation	Aliphatic hydroxylation
**Ampicillin**	N-dealkylation	S-oxidation	Amine hydroxylation	Aliphatic hydroxylation

Table S13 (L). Compound metabolism assessed using SmartCyp and SOMP.

Compound	3A4		2D6		2C9	
	The most reactive atom	Score	The most reactive atom	Score	The most reactive atom	Score
**Phosphinothricin**	C7	35.4	C7	101.0	N8	75.8
**Methionine sulfoximine**	C7	35.4	N3	72.3	N3	64
**Glutamic acid**	C2	35.4	C2	101.0	N1	75.8
**Ampicillin**	C13	35.4	S6	76.6	C13	64.4

Table S13 (M). Bioactivity assessed using Molinspiration.

Compound	GPCR Ligand	Ion channel modulator	Kinase inhibitor	Nuclear receptor ligand	Protease inhibitor	Enzyme inhibitor
**Phosphinothricin**	0.57	1.51	-0.43	-1.35	1.34	1.52
**Methionine sulfoximine**	-0.47	0.1	-1.3	-0.91	-0.15	0.71
**Glutamic acid**	-0.29	0.25	-1.07	-0.96	-0.16	0.23
**Ampicillin**	0.04	-0.47	-0.71	-0.61	0.87	0.25

Table S13 (N). Bioactivity assessed using the SWISSTarget predictor (most probable molecular targets and their identification data).

Compound	Target	Common name	Uniprot ID	Target Class	Probability
**Phosphinothricin**	Metabotropic glutamate receptor 4	GRM4	Q14833	Family C G protein-coupled receptor	0.130293
**Methionine sulfoximine**	Tyrosine 3-hydroxylase	TH	P07101	Oxidoreductase	0.063025
**Methionine sulfoximine**	Kynureninase	KYNU	Q16719	Enzyme	0.063025
**Methionine sulfoximine**	Tyrosine-protein kinase FYN	FYN	P06241	Kinase	0.063025
**Methionine sulfoximine**	Epidermal growth factor receptor erbB1	EGFR	P00533	Kinase	0.063025

Compound	Target	Common name	Uniprot ID	Target Class	Probability
**Methionine sulfoximine**	Tyrosine-protein kinase LCK	LCK	P06239	Kinase	0.063025
**Glutamic acid**	-	-	-	-	-
**Ampicillin**	Integrin alpha-4/ beta-1	ITGB1 ITGA4	P05556 P13612	Membrane receptor	0.112748

Table S13 (O). Anticarcinogenic effect: most probable cell lines for which compounds exhibit cytotoxicity (Pa>0.5).

Compound	Pa	Pi	Cell line	Cell-line full name	Tissue	Tumour type
**Phosphinothricin**	0.722	0.008	HCT-116	Colon carcinoma	Colon	Carcinoma
**Phosphinothricin**	0.512	0.005	MDA-MB-453	Breast adenocarcinoma	Breast	Adenocarcinoma
**Methionine sulfoximine**	0.721	0.003	NCI-H1299	Non-small cell lung carcinoma	Lung	Carcinoma
**Methionine sulfoximine**	0.514	0.005	MDA-MB-453	Breast adenocarcinoma	Breast	Adenocarcinoma
**Methionine sulfoximine**	0.507	0.004	BXPC-3	Pancreatic adenocarcinoma	Pancreas	Adenocarcinoma
**Methionine sulfoximine**	0.510	0.037	Kasumi 1	Childhood acute myeloid leukaemia with maturation	Haematopoietic and lymphoid tissue	Leukaemia
**Glutamic acid**	0.773	0.003	NCI-H1299	Non-small cell lung carcinoma	Lung	Carcinoma
**Glutamic acid**	0.554	0.004	BXPC-3	Pancreatic adenocarcinoma	Pancreas	Adenocarcinoma
**Glutamic acid**	0.525	0.016	IGROV-1	Ovarian adenocarcinoma	Ovarium	Adenocarcinoma
**Glutamic acid**	0.511	0.034	DMS-114	Lung carcinoma	Lung	Carcinoma
**Ampicillin**	-	-	-	-	-	-

Table S13 (P). Mechanisms of action and adverse/toxic effects (Pa>Pi).

Compound	Mechanism of action			Toxic effects		
	Pa	Pi	Activity	Pa	Pi	Activity
**Phosphinothricin**	0.928	0.004	Glutamate-5-semialdehyde dehydrogenase inhibitor	0.924	0.004	Pure red cell aplasia
	0.883	0.000	Glutamate (mGluR6) agonist	0.725	0.033	Ocular toxicity
	0.876	0.000	Glutamate (mGluR group III)	0.692	0.030	Respiratory failure

Compound	Mechanism of action			Toxic effects		
	Pa	Pi	Activity	Pa	Pi	Activity
			agonist			
	0.859	0.000	Glutamate (mGluR4) agonist	0.666	0.023	Bronchoconstrictor
	0.833	0.014	Methylenetetrahydrofolate reductase (NADPH) inhibitor	0.661	0.036	Nephrotoxic
**Methionine sulfoximine**	0.927	0.004	Angiogenesis inhibitor	0.725	0.020	Teratogen
	0.921	0.004	Antiinflammatory	0.700	0.022	Embryotoxic
	0.892	0.002	Phenylalanine(histidine) transaminase inhibitor	0.624	0.014	Anemia, sideroblastic
	0.886	0.005	Acylcarnitine hydrolase inhibitor	0.608	0.011	Bullous pemphigoid
	0.877	0.001	CDK1/cyclin B inhibitor	0.636	0.053	Pure red cell aplasia
**Glutamic acid**	0.971	0.001	Acylcarnitine hydrolase inhibitor	0.973	0.006	Toxic, respiration
	0.963	0.002	Methylenetetrahydrofolate reductase (NADPH) inhibitor	0.958	0.004	Euphoria
	0.960	0.001	Glutamine-phenylpyruvate transaminase inhibitor	0.938	0.004	Pure red cell aplasia
	0.957	0.001	Dimethylargininase inhibitor	0.904	0.004	Ulcer, aphthous
	0.956	0.002	Protein-disulfide reductase (glutathione) inhibitor	0.897	0.002	Anemia, sideroblastic
**Ampicillin**	0.984	0.001	Muramoyltetrapeptide carboxypeptidase inhibitor	0.983	0.002	Hyperthermic
	0.892	0.002	Acylaminoacyl-peptidase inhibitor	0.980	0.001	Hematuria
	0.876	0.002	Penicillin amidase inhibitor	0.978	0.003	Diarrhea
	0.852	0.001	Beta lactamase inhibitor	0.977	0.003	Hepatitis
	0.824	0.001	Deacetoxycephalosporin-C synthase inhibito	0.974	0.003	Leukopenia

Table S13 (Q). Acute toxicity in rodents when administered intraperitoneally, intravenously, orally and subcutaneously: LD50 in mg/kg.

Compound	Rat IP LD50 (mg/kg)	Rat IV LD50 (mg/kg)	Rat Oral LD50 (mg/kg)	Rat SC LD50 (mg/kg)
**Phosphinothricin**	99.610 in AD	164.200 in AD	2390.000 in AD	158.300 in AD
**Methionine sulfoximine**	169.000 in AD	461.000 in AD	2121.000 in AD	275.300 in AD
**Glutamic acid**	2591.000 in AD	2306.000 in AD	8750.000 in AD	1789.000 in AD
**Ampicillin**	3882.000 in AD	3274.000 in AD	7122.000 in AD	4185.000 in AD

Table S13 (R). Acute toxicity in rodents. Classification of Chemicals by OECD Project.

Compound	Rat IP LD50 Classification	Rat IV LD50 Classification	Rat Oral LD50 Classification	Rat SC LD50 Classification
**Phosphinothricin**	Class 4 in AD	Class 4 in AD	Class 5 in AD	Class 4 in AD
**Methionine sulfoximine**	Class 4 in AD	Class 5 in AD	Class 5 in AD	Class 4 in AD
**Glutamic acid**	Non-Toxic in AD	Non-Toxic in AD	Non-Toxic in AD	Class 5 in AD
**Ampicillin**	Non-Toxic in AD	Non-Toxic in AD	Non-Toxic in AD	Non-Toxic in AD

Table S14. Molecular dynamics simulation results for phosphinothricin, methionine sulfoximine, glutamic acid and ampicillin.

Table S14 (A). Molecular dynamics simulation results for phosphinothricin.

Step	Time	Potential energy (J)	Kinetic energy (J)
0	0.0	70984050134.665192	17649630034.095348
100	0.1	59617762094.141769	29018144699.701870
200	0.2	33624709116.687840	54736887714.538490
300	0.3	37645445501.530022	50757945118.525673
400	0.4	39797975676.871201	48497400955.978470
500	0.5	22356241679.548798	65788436802.400925
600	0.6	26819690545.799023	61534101740.961647
700	0.7	27707783530.155140	60349372013.520531
800	0.8	26228140325.677742	62112767850.083595
900	0.9	34586331635.642586	53759494273.691803
1000	1	60848576900.851852	27579401287.603355

Table S14 (B). Molecular dynamics simulation results for methionine sulfoximine.

Step	Time	Potential energy (J)	Kinetic energy (J)
0	0,0	-7.246277	112.850105
100	0,1	12.799410	92.907652
200	0,2	41.244191	64.689609
300	0,3	4.077037	101.295037
400	0,4	10.099424	95.784242
500	0,5	11.476127	94.100435
600	0,6	43.183180	62.758407
700	0,7	8.595850	96.926621
800	0,8	16.903825	89.079292
900	0,9	17.561657	88.057453
1000	1	12.904022	93.316199

Table S14 (C). Molecular dynamics simulation results for glutamic acid.

Step	Time	Potential energy (J)	Kinetic energy (J)
0	0,0	29.898415	71.027669
100	0,1	48.948327	52.053117
200	0,2	24.728076	75.854390
300	0,3	29.036512	71.779454
400	0,4	27.661968	73.274734
500	0,5	29.280999	71.495538
600	0,6	37.225955	64.040151
700	0,7	40.725388	59.927943
800	0,8	40.613214	60.750139
900	0,9	38.849046	62.104697
1000	1	26.331205	74.756608

Table S14 (D). Molecular dynamics simulation results for ampicillin.

Step	Time	Potential energy (J)	Kinetic energy (J)
0	0,0	273.742527	149.966886
100	0,1	267.842493	155.898570
200	0,2	251.843917	171.523523
300	0,3	286.427591	136.824938
400	0,4	258.661823	164.364377
500	0,5	274.136545	149.045394
600	0,6	265.744426	157.635224
700	0,7	271.681095	151.851149
800	0,8	284.590500	139.364032
900	0,9	273.402343	150.206466
1000	1	295.206664	128.533068

Table S15. Molecular docking results for phosphinothricin, methionine sulfoximine, glutamic acid and ampicillin [37].

Table S15 (A). Molecular docking results for phosphinothricin.

Nr	Score	Interface area
1	6200	1123.03
2	3000	640.87
3	1700	444.33
4	800	329.91
5	700	386.49
6	600	454.29
7	400	367.2
8	0	372.75
9	0	157.72
10	0	3.18

Table S15 (B). Molecular docking results for methionine sulfoximine.

Nr	Score	Interface area
1	8000	1123.03
2	7900	372.75
3	6900	157.72
4	6300	640.87
5	6300	454.29
6	5200	318.53
7	4900	329.91
8	4600	386.49
9	3800	444.33
10	3500	367.2

Table S15 (C). Molecular docking results for glutamic acid.

Nr	Score	Interface area
1	8000	1123.03
2	6300	640.87
3	3800	444.33

Nr	Score	Interface area
4	4900	329.91
5	4600	386.49
6	6300	454.29
7	3500	367.2
8	2900	226.54
9	1700	287.79
10	1600	211.55

Table S15 (D). Molecular docking results for ampicillin.

Nr	Score	Interface area
1	0.8	1123.03
2	0.79	372.75
3	0.69	157.72
4	0.63	640.87
5	0.63	454.29
6	0.52	318.53
7	0.49	329.91
8	0.46	386.49
9	0.38	444.33
10	0.35	367.2

Figure S3. Molecular docking results. Interaction with glutamine synthetase of (a) phosphinotricin, (b) methionine sulfoximine, (c) glutamic acid and (d) ampicillin [37].

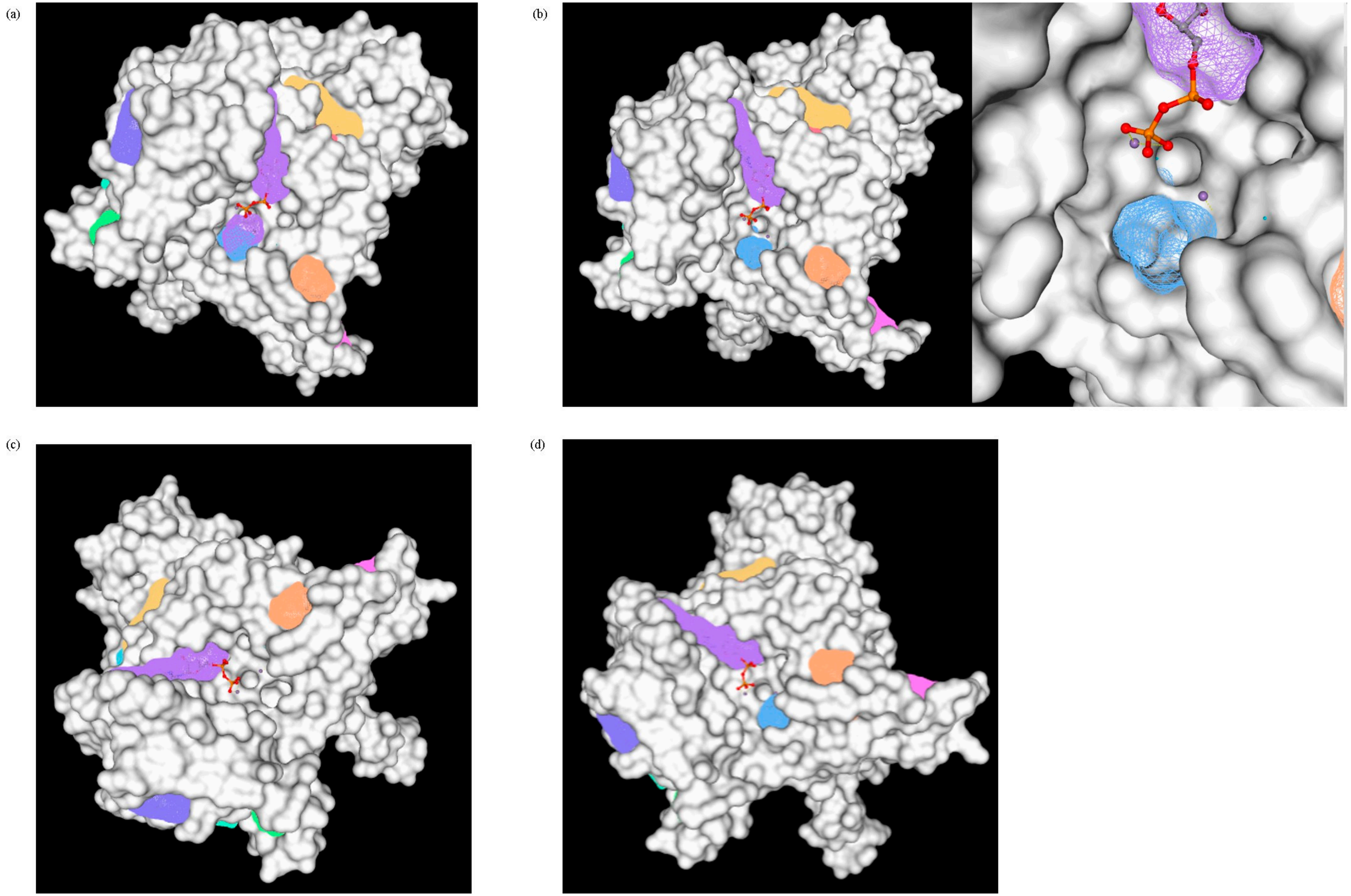

Table S16. Grid sizes used in Swissdock and energetic values of the most probable ligand-receptor complexes for phosphinothricin, methionine sulfoximine, glutamic acid and ampicillin

Compound	Conformations	Size of grid			Center			Cluster	ΔG (kcal/mol)	FullFitness (kcal/mol)	Ki
		Width	Length	Height	x	y	z				
**Glutamic acid**	257	15.72	15.44	15.5	-85.9047	13.5037	-68.0569	2	-8.6	-2188.8	45.437×10^{-8}
**Phosphinothricin**	257	14.06	15.63	15.00	-90.9519	13.8641	-82.4948	27	-8.8	-2183.2	31.6478×10^{-8}
**Methionine sulfoximine**	257	13.00	12.53	12.43	-91.3579	12.8023	-86.285	2	-8.0	-2179.1	13.575×10^{-7}
**Ampicillin**	257	14.6	17.45	15.87	-80.8418	4.5479	-57.6088	2	-9.3	-2028.4	14.7347×10^{-8}

Table S17. Molecular docking results obtained using AutoDock Vina for phosphinothricin, methionine sulfoximine, glutamic acid and ampicillin.

Table S17 (A). Molecular docking results obtained using AutoDock Vina for phosphinothricin. Run time: 28.3 seconds.

Mode	Affinity (kcal/mol)	Dist from RMSD L. B	Dist from RMSD U. B
1	-1.1	0	0
2	-1.1	6.947	6.947
3	-1.0	15.171	15.171
4	-0.9	12.825	12.825
5	-0.9	19.823	19.823
6	-0.9	13.155	13.155
7	-0.8	16.552	16.552
8	-0.8	16.683	16.683
9	-0.8	16.589	16.589

Table S17 (B). Molecular docking results obtained using AutoDock Vina for methionine sulfoximine. Run time: 28.3 seconds.

Mode	Affinity (kcal/mol)	Dist from RMSD L. B	Dist from RMSD U. B
1	-6.3	0	0
2	-5.7	1.607	4.178

Mode	Affinity (kcal/mol)	Dist from RMSD L. B	Dist from RMSD U. B
3	-5.6	1.371	1.576
4	-5.3	1.835	4.564
5	-4.9	4.021	6.687
6	-4.9	2.205	2.9
7	-4.7	2.974	5.147
8	-4.7	5.749	6.525
9	-4.5	3.78	6.519

Table S17 (C). Molecular docking results obtained using AutoDock Vina for glutamic acid. Run time: 28.3 seconds.

Mode	Affinity (kcal/mol)	Dist from RMSD L. B	Dist from RMSD U. B
1	-1.1	0	0
2	-1.0	9.244	9.244
3	-0.9	7.399	7.399
4	-0.9	11.768	11.768
5	-0.9	7.325	7.325
6	-0.9	14.969	14.969
7	-0.8	14.634	14.634
8	-0.8	12.111	12.111
9	-0.8	10.39	10.39

Table S17 (D). Molecular docking results obtained using AutoDock Vina for ampicillin. Run time: 28.3 seconds.

Mode	Affinity (kcal/mol)	Dist from RMSD L. B	Dist from RMSD U. B
1	-7.3	0	0
2	-7.1	2.509	6.605
3	-6.7	1.462	2.226

Mode	Affinity (kcal/mol)	Dist from RMSD L. B	Dist from RMSD U. B
4	-6.4	2.628	6.805
5	-6.4	2.462	6.322
6	-6.3	2.01	6.305
7	-6.3	3.51	6.753
8	-6.1	3.221	6.501
9	-6.0	4.104	6.77

Table S18. Grid sizes used in AutoDock Vina for phosphinothricin, methionine sulfoximine, glutamic acid and ampicillin.

Compound	Size of grid			Center		
	Width	Length	Height	x	y	z
**Phosphinothricin**	19.1024	18.23	17.529	-4.1966	-16.3175	-71.1498
**Methionine sulfoximine**	13.43	15.59	19.19	-5.8989	-16.1835	-66.3292
**Glutamic acid**	25.56	19.33	12.445	-2.7569	-17.2695	-68.7373
**Ampicillin**	18.603	19.828	19.28	-4.7314	-17.317	-69.2087

REFERENCES

1. Systèmes, D. BIOVIA Draw for Academics (Version 21.1.0.2363). Available online: https://discover.3ds.com/biovia-draw-academic (accessed on 12 January 2023).
2. Marvinsketch (Version 5.0). Available online: https://chemaxon.com/marvin (accessed on 19 January 2023).
3. AquaSol: Predict Aqueous Solubility of Small Molecules Using UG-RNN Ensembles (Version 2.0). Available online: http://cdb.ics.uci.edu/cgibin/tools/AquaSolWeb.py (accessed on 21 January 2023).
4. Chemicalize - Instant Cheminformatics Solutions. Available online: https://chemicalize.com (accessed on 20 January 2023).
5. Jeliazkova, N.; Martinov, M.; Tcheremenskaia, O.; JK; Networks, M.; Rydberg, P.; Avramova, S.; Kochev, N.; Jeliazkov, V.; et al. Toxtree – Toxtree - Toxic Hazard Estimation by Decision Tree Approach (Version 3.1.0.1851). Available online: https://toxtree.sourceforge.net/ (accessed on 26 January 2023).
6. Molecular Properties Prediction - Osiris Property Explorer. Available online: https://www.organic-chemistry.org/prog/peo/ (accessed on 13 February 2023).
7. Drug Likeness Tool (DruLiTo 1) (Version 1.0.). Available online: http://www.niper.gov.in/pi_dev_tools/DruLiToWeb/DruLiTo_index.html (accessed on 01 February 2023).
8. SMARTCyp (Version 3.0). Available online: https://smartcyp.sund.ku.dk/mol_to_som (accessed on 03 February 2023).
9. Calculation of Molecular Properties and Bioactivity Score. Available online: https://www.molinspiration.com/cgi-bin/properties (accessed on 05 February 2023).
10. SwissADME. Available online: http://www.swissadme.ch/ (accessed on 17 January 2023).
11. Daina, A.; Michielin, O.; Zoete, V. SwissADME: A Free Web Tool to Evaluate Pharmacokinetics, Drug-Likeness and Medicinal Chemistry Friendliness of Small Molecules. *Sci Rep* **2017**, *7*, 42717, doi:10.1038/srep42717.
12. SwissTargetPrediction. Available online: http://www.swisstargetprediction.ch/ (accessed on 08 February 2023).
13. SwissDock - The Online Docking Web Server of the Swiss Institute of Bioinformatics – TargetProteins. Available online: http://www.swissdock.ch/target_proteins (accessed on 11 February 2023).
14. Lagunin, A.A.; Dubovskaja, V.I.; Rudik, A.V.; Pogodin, P.V.; Druzhilovskiy, D.S.; Gloriozova, T.A.; Filimonov, D.A.; Sastry, N.G.; Poroikov, V.V. CLC-Pred: A Freely Available Web-Service for in Silico Prediction of Human Cell Line Cytotoxicity for Drug-like Compounds. *PLoS ONE* **2018**, *13*, e0191838, doi:10.1371/journal.pone.0191838.
15. Gusar – Create QSAR/QSPR Models on the Basis of the Appropriate Training Sets. Available online: http://www.way2drug.com/gusar/acutoxpredict.html (accessed on 29 January 2023).
16. Prediction of Site of Metabolism (Version 2.0). Available online: http://www.way2drug.com/SOMP/ (accessed on 04 February 2023).
17. Way2Drug – Main (Version 2.0). Available online: http://www.way2drug.com/PASSOnline/index.php (accessed on 10 February 2023).
18. AutoDock Vina (Version 1.1.2). Available online: https://vina.scripps.edu/ (accessed on 12 February 2023).
19. Download UCSF Chimera (Version 1.15). Available online: https://www.cgl.ucsf.edu/chimera/download.html (accessed on 12 February 2023).
20. PatchDock Server: An Automatic Server for Molecular Docking (Version Beta 1.3). Available online: https://bioinfo3d.cs.tau.ac.il/PatchDock/php.php (accessed on 11 February 2023).
21. Schneidman-Duhovny, D.; Inbar, Y.; Polak, V.; Shatsky, M.; Halperin, I.; Benyamini, H.; Barzilai, A.; Dror, O.; Haspel, N.; Nussinov, R.; et al. Taking Geometry to Its Edge: Fast Unbound Rigid (and Hinge-Bent) Docking. *Proteins* **2003**, *52*, 107–112, doi:10.1002/prot.10397.
22. SwissSimilarity. Available online: http://www.swisssimilarity.ch/ (accessed on 13 February 2023).
23. Delaney, J.S. ESOL: Estimating Aqueous Solubility Directly from Molecular Structure. *J. Chem. Inf. Comput. Sci.* **2004**, *44*, 1000–1005, doi:10.1021/ci034243x.
24. Ali, J.; Camilleri, P.; Brown, M.B.; Hutt, A.J.; Kirton, S.B. Revisiting the General Solubility Equation: *In Silico* Prediction of Aqueous Solubility Incorporating the Effect of Topographical Polar Surface Area. *J. Chem. Inf. Model.* **2012**, *52*, 420–428, doi:10.1021/ci200387c.
25. Arnott, J.A.; Planey, S.L. The Influence of Lipophilicity in Drug Discovery and Design. *Expert Opinion on Drug Discovery* **2012**, *7*, 863–875, doi:10.1517/17460441.2012.714363.
26. Mannhold, R.; Poda, G.I.; Ostermann, C.; Tetko, I.V. Calculation of Molecular Lipophilicity: State-of-the-Art and Comparison of LogP Methods on More than 96,000 Compounds. *Journal of Pharmaceutical Sciences* **2009**, *98*, 861–893, doi:10.1002/jps.21494.
27. Cheng, T.; Zhao, Y.; Li, X.; Lin, F.; Xu, Y.; Zhang, X.; Li, Y.; Wang, R.; Lai, L. Computation of Octanol−Water Partition Coefficients by Guiding an Additive Model with Knowledge. *J. Chem. Inf. Model.* **2007**, *47*, 2140–2148, doi:10.1021/ci700257y.

28. Wildman, S.A.; Crippen, G.M. Prediction of Physicochemical Parameters by Atomic Contributions. *J. Chem. Inf. Comput. Sci.* **1999**, *39*, 868–873, doi:10.1021/ci990307l.
29. Moriguchi, I.; Hirono, S.; Liu, Q.; Nakagome, I.; Matsushita, Y. Simple Method of Calculating Octanol/Water Partition Coefficient. *Chem. Pharm. Bull.* **1992**, *40*, 127–130, doi:10.1248/cpb.40.127.
30. Moriguchi, I.; Hirono, S.; Nakagome, I.; Hirano, H. Comparison of Reliability of Log P Values for Drugs Calculated by Several Methods. *Chem. Pharm. Bull.* **1994**, *42*, 976–978, doi:10.1248/cpb.42.976.
31. Pettersen, E.F.; Goddard, T.D.; Huang, C.C.; Couch, G.S.; Greenblatt, D.M.; Meng, E.C.; Ferrin, T.E. UCSF Chimera?A Visualization System for Exploratory Research and Analysis. *J. Comput. Chem.* **2004**, *25*, 1605–1612, doi:10.1002/jcc.20084.
32. Berman, H.M.; Battistuz, T.; Bhat, T.N.; Bluhm, W.F.; Bourne, P.E.; Burkhardt, K.; Feng, Z.; Gilliland, G.L.; Iype, L.; Jain, S.; et al. The Protein Data Bank. *Acta Crystallogr D Biol Crystallogr* **2002**, *58*, 899–907, doi:10.1107/S0907444902003451.
33. Meng, E.C.; Pettersen, E.F.; Couch, G.S.; Huang, C.C.; Ferrin, T.E. Tools for Integrated Sequence-Structure Analysis with UCSF Chimera. *BMC Bioinformatics* **2006**, *7*, 339, doi:10.1186/1471-2105-7-339.
34. Trott, O.; Olson, A.J. AutoDock Vina: Improving the Speed and Accuracy of Docking with a New Scoring Function, Efficient Optimization, and Multithreading. *J. Comput. Chem.* **2009**, NA-NA, doi:10.1002/jcc.21334.
35. PubChem. Available online: https://pubchem.ncbi.nlm.nih.gov/ (accessed on 17 January 2023).
36. Patlewicz, G.; Jeliazkova, N.; Safford, R.J.; Worth, A.P.; Aleksiev, B. An Evaluation of the Implementation of the Cramer Classification Scheme in the Toxtree Software. *SAR and QSAR in Environmental Research* **2008**, *19*, 495–524, doi:10.1080/10629360802083871.
37. ProteinsPlus. Available online: https://proteins.plus/ (accessed on 20 April 2023).
